# Synergistic and Antagonistic Interplay between Myostatin Gene Expression and Physical Activity Levels on Gene Expression Patterns in Triceps Brachii Muscles of C57/BL6 Mice

**DOI:** 10.1371/journal.pone.0116828

**Published:** 2015-02-24

**Authors:** Kelsey Caetano-Anollés, Sanjibita Mishra, Sandra L. Rodriguez-Zas

**Affiliations:** 1 Department of Animal Sciences, University of Illinois at Urbana-Champaign, Urbana, Illinois, United States of America; 2 Khorana Scholars Program, Indo-US Science and Technology Forum, New Delhi, India; 3 National Institute of Technology, Rourkel, India; 4 Department of Statistics, University of Illinois at Urbana-Champaign, Urbana, Illinois, United States of America; 5 Institute for Genomic Biology, University of Illinois at Urbana-Champaign, Urbana, Illinois, United States of America; Rutgers University -New Jersey Medical School, UNITED STATES

## Abstract

Levels of myostatin expression and physical activity have both been associated with transcriptome dysregulation and skeletal muscle hypertrophy. The transcriptome of triceps brachii muscles from male C57/BL6 mice corresponding to two genotypes (wild-type and myostatin-reduced) under two conditions (high and low physical activity) was characterized using RNA-Seq. Synergistic and antagonistic interaction and ortholog modes of action of myostatin genotype and activity level on genes and gene pathways in this skeletal muscle were uncovered; 1,836, 238, and 399 genes exhibited significant (FDR-adjusted P-value < 0.005) activity-by-genotype interaction, genotype and activity effects, respectively. The most common differentially expressed profiles were (i) inactive myostatin-reduced relative to active and inactive wild-type, (ii) inactive myostatin-reduced and active wild-type, and (iii) inactive myostatin-reduced and inactive wild-type. Several remarkable genes and gene pathways were identified. The expression profile of nascent polypeptide-associated complex alpha subunit (Naca) supports a synergistic interaction between activity level and myostatin genotype, while Gremlin 2 (Grem2) displayed an antagonistic interaction. Comparison between activity levels revealed expression changes in genes encoding for structural proteins important for muscle function (including troponin, tropomyosin and myoglobin) and for fatty acid metabolism (some linked to diabetes and obesity, DNA-repair, stem cell renewal, and various forms of cancer). Conversely, comparison between genotype groups revealed changes in genes associated with G1-to-S-phase transition of the cell cycle of myoblasts and the expression of Grem2 proteins that modulate the cleavage of the myostatin propeptide. A number of myostatin-feedback regulated gene products that are primarily regulatory were uncovered, including microRNA impacting central functions and Piezo proteins that make cationic current-controlling mechanosensitive ion channels. These important findings extend hypotheses of myostatin and physical activity master regulation of genes and gene pathways, impacting medical practices and therapies associated with muscle atrophy in humans and companion animal species and genome-enabled selection practices applied to food-production animal species.

## Introduction

Genetic and non-genetic conditions impact the molecular pathways and physiology of the skeletal muscle. The myostatin (Mstn) gene encodes a growth and differentiating factor and hormonal protein responsible for inhibition of muscle growth and proliferation in vertebrates. Myostatin negatively regulates muscle fiber number during skeletal muscle development [1–2] and inhibits myogenic differentiation by reducing mRNA levels of the muscle regulatory factors [3–4]. Conversely, myostatin deficiency caused by alterations at the DNA or RNA level is linked to increased numbers and size of muscle fibers, a phenomenon often referred to as ‘double muscling [5]. Likewise, myostatin-deficient Cre/loxP mice show hyperplasia (increased number of muscle fibers) and hypertrophy in the skeletal muscle [6].

Physical activity influences muscle fiber in manners akin to the effect of myostatin deficiency [7]. Contractile activity in muscle promotes changes in the synthesis and degradation of contractile and metabolic proteins that allow muscles to optimize, adapt, and endure activity [8–10]. Activity causes an immediate sarcolemmal disruption that damages the cytoskeletal network. Muscle inflammation caused by physical activity is accompanied by an increase of nitric oxide (NO) production, and skeletal muscle derived NO regulates contraction and metabolism as well as modulates muscle glucose uptake during activity [11].

While targeted genetic and non-genetic studies have associated skeletal muscle hypertrophy to dysregulation of the IGF1-Akt-mTOR and myostatin-Smad2/3 signaling pathways, muscle atrophy has been associated to dysregulation of the autophagic-lysosomal and proteasomal pathways [12]. Studies of the effect of myostatin deficiency using transgenic null myostatin (-/-) mice and quantitative real time PCR (qRT-PCR) identified over-expression of genes involved in myogenesis, protein degradation, extracellular matrix components and mitochondrial ATP synthesis [13]. Likewise, studies of the impact of physical activity on gene expression in the skeletal muscle using qRT-PCR demonstrated over-expression of myostatin and follistatin and under-expression of the myostatin receptor Activin IIB (ActRIIB) in murine limb muscles after acute physical activity [14].

Few studies have evaluated the simultaneous effects of physical activity and myostatin genotype on the transcripts in the skeletal muscle of mice [4, 15]. Results from one study demonstrated that the area of hypertrophic myofibres (extensor digitorum longus, gastrocnemius and rectus femoris muscles) in myostatin-depleted mice decreased towards wild-type levels meanwhile BCL2/Adenovirus E1B 19kDa Interacting Protein 3 (Bnip3), a key autophagy-related gene, was over-expressed in response to endurance exercise [4]. Also, qRT-PCR profiling confirmed that activity increased the expression of the Uncoupling Protein 3 Mitochondrial, Proton Carrier (Ucp3), Carnitine Palmitoyltransferase 1A (Cpt1a), Pyruvate Dehydrogenase Kinase Isozyme 4 (Pdk4), and estrogen-related receptor-γ (Errγ) genes [4]. Investigations focusing on the expression of a myostatin target gene, Mighty, using qRT-PCR suggested that acute resistance exercise decreased myostatin signaling in the skeletal muscle (soleus, plantaris and tibialis anterior muscles) of rats through the activation of the TGFβ inhibitor Notch. The activation of this inhibitor resulted in lower myostatin transcriptional activity that correlated with muscle hypertrophy [14]. A report of the effect of myostatin knockdown and exercise (swimming) in gastrocnemius muscle demonstrated the over-expression of Pax-7 on knockdown mice without exercise relative to all other groups and over-expression of Myo-D on all knockdown mice irrespective of exercise level [15]. These previous studies have offered insights into the relationship between myostatin, physical activity and gene expression. However, studies that simultaneously consider genetic and non-genetic factors using high-throughput sequencing-based techniques could allow for a more comprehensive understanding of molecular networks of the skeletal muscle.

This study characterizes the complete transcriptome of triceps brachii muscles from C57/BL6 mice representing one of two genotype transcript levels (wild-type or myostatin typical and myostatin-reduced) and one of two physical activity levels (high and low) using massive parallel next-generation RNA sequencing. Synergistic, antagonistic and ortholog modes of action of the factors myostatin genotype and activity on genes and gene pathway profiles were investigated. This study is supported by: (a) mapping RNA sequencing reads to the mouse genome, identification of differentially expressed genes, and testing for differential expression among activity-genotype combination groups; (b) identification and interpretation of gene profiles revealing significant interaction between genotype and activity; (c) identification and interpretation of gene profiles revealing significant genotype (or activity) effect irrespective of activity (or genotype); and (d) functional analysis in support of the identification and interpretation of biological processes and pathways associated with genotype and activity levels. Our findings provide a basis to understand multifactorial gene regulation and dysregulation in triceps brachii and other skeletal muscles of mice.

## Materials and Methods

### Experiment

Profiling information stems from an experiment comparing the transcriptome of a skeletal muscles, triceps brachii muscle of adult (6 months of age) male C57/BL6 mice. Difference in gene expression associated with two factors were studied. The factor termed genotype encompasses two levels: wild-type mice exhibiting baseline expression of the myostatin gene and myostatin-reduced mice exhibiting lower expression of the myostatin gene. The factor termed activity encompasses two levels: inactive and active. Four physical activity-by-genotype combination groups of mice were compared (n = 3/group): (1) active and myostatin-reduced, (2) inactive and wild-type (control genotype); (3) inactive and myostatin-reduced; and (4) active and wild-type. Prior to the trial, mice were housed in standard cages in groups of 2 or 3, given *ad libitum* access to food and water and kept in a 12-hour dark cycle. Myostatin-reduced mice were developed from C57/BL6 mice with floxed myostatin that was activated using Cre recombinase. At 4 months of age, all mice were fed chow with 0.025% tamoxifen content for 6 weeks to activate the Cre Recombinase enzyme and deplete myostatin only in mice with floxed myostatin genes. Myostatin was under-expressed (approximately 85%) in the myostatin-reduced mice. One week after the end of the tamoxifen feeding (approximately 6 months of age), mice in the active group were moved to be housed individually and given free access to running wheels during the last 12 weeks of the study. Physical activity was monitored and the sum over 1-hour periods was recorded. At the end of the wheel-running period, all mice were euthanized and samples taken from their triceps brachii muscles were frozen in melting isopentane and stored at −70°C. Muscles were sampled from myostatin-reduced and control mice that were matched for running behavior; this was done to ensure that the amount of physical activity performed was not a contributor to differences in gene expression between control and myostatin-reduced mice. Polyadenylated RNA was extracted as directed by Invitrogen using a Trizol reagent, converted to cDNA, and amplified with an Illumina TruSeq RNA library preparation kit following manufacturer instructions.

Triceps brachii muscle transcriptome was studied using Illumina Genome Analyzer IIx (Illumina, Inc. San Diego, CA) producing 65-base long single-end reads. Data processing was performed using CASAVA software. The 65-base sequence reads were mapped to the mouse genome (mm9) using default settings and reads mapping to exons in the Refseq database (http://www.ncbi.nlm.nih.gov/refseq/) of transcripts were counted. Exon-level data was consolidated to gene-level read counts and summarized. Data normalization was performed so trimmed column means (excluding the highest and lowest 5th percentiles) were equal for all samples. In total, 13 protein-coding mtDNA transcripts were mapped to NC_005089.1, counted separately, and normalized to the trimmed mean of the non-mitochondrial transcripts. The transcriptome data, additional experimental details and preliminary analysis are available in the National Center for Biotechnology Information, Gene Expression Omnibus database, accession number GSE31839 [16]. Results from these preliminary analysis uncovered a main effect of activity on genes associated with oxidative energy metabolism and no interaction between activity and myostatin levels. Results from advanced modeling and functional analysis of the experiment are presented.

### Analysis

The 65-base, single-end sequence reads from FastQ files were mapped to the mouse mm10 genome assembly accessed from the UCSC Genome Browser database (http://genome.ucsc.edu). Prior to mapping, FastqGroomer was used to convert file format to FastqSanger [17] and FastQC was used for quality control of the reads [18]. Using Fastq Quality Trimmer, 3′ end positions that exhibited Phred quality values < 20 were removed. Data was normalized so trimmed transcript count means, excluding values in the upper and lower 5 percentiles, were the same for all samples. The 13 protein-coding mtDNA transcripts were mapped to NC_005089.1 (*Mus musculus* mitochondrion, complete genome), counted separately, and normalized to the trimmed mean of non-mitochondrial transcript counts. Sequence reads were mapped using Tophat (v1.4.0), transcript isoforms were identified, quantified in number of fragments per kilobase of exon per million mapped reads (FPKM) [16], and differential transcript abundance was tested using Cufflinks routines including Cuffmerge, and Cuffdiff (v2.1.1) with default settings [19]. Differential expression was tested between activity-genotype groups, between activity groups and between genotype groups to determine the statistical significance of interaction and main effects on individual transcript isoforms [20–21]. False discovery rate (FDR) adjusted P-values were used to account for multiple test adjustment across transcripts [22–24]. The vast majority of the differentially expressed genes were represented by one transcript and thus, results are discussed on a gene basis. The routine workflow was implemented in Galaxy [25–27]. Comparisons of lists of differentially expressed genes among contrasts between pairs of activity-genotype combinations were visualized with Venn diagrams, created using VENNY, an online interactive tool for comparing lists [28].

Enrichment of functional categories and pathways among the differentially expressed genes was explored using the web service Database for Analysis, Validation, and Integrated Discovery (DAVID; http://david.abcc.ncifcrf.gov). The Gene Ontology (GO) functional categories investigated included biological process (BP) and molecular function (MF), the biological objective to which the gene or its product contributes or the biochemical activity of a gene product, respectively [29], and pathways were those defined by the Kyoto Encyclopedia of Genes and Genomes (KEGG) [30]. Minimization of redundancy within BP, MF and KEGG terms was attained by using GO Functional Annotation Tool (FAT) categories, which comprise more general BP and MF terms while filtering broad and less informative terms [31]. Statistical significance of the individual FAT or KEGG categories was based on the EASE Scores (modified Fisher Exact) available in DAVID. Further minimization of redundancy between FAT and KEGG categories was achieved through the use of clusters of categories that share genes. The statistical significance of these clusters was assessed using the enrichment score that corresponds to the geometric mean of the EASE Scores of the functional categories in the cluster [32]. Evaluation of clusters of categories enabled the discovery of molecular processes that respond to variation in physical activity and myostatin genotype levels.

Beyond the identification of differentially expressed genes exhibiting significant physical activity-by-genotype interaction, this study aimed at uncovering the synergistic or antagonistic interplay between these factors. Six pairwise contrasts were used to profile the expression patterns: active wild-type vs inactive myostatin-reduced [AW-IM], active myostatin-reduced vs active wild-type [AM-AW], active myostatin-reduced vs inactive myostatin-reduced [AM-IM], inactive wild-type vs active wild-type [IW-AW], inactive wild-type vs active myostatin-reduced [IW-AM], and inactive wild-type vs inactive myostatin-reduced [IW-IM]. Among the genes that exhibited significant (FDR-adjusted P-value < 0.001) activity-by-genotype interaction, alternative profiles of over- and under-expression or non-significant (raw P-value < 0.00005 or FDR-adjusted P-value < 0.05) differential expression in each of the six contrasts between pairs of activity-genotype combinations were identified. The concept of synergism and antagonism has been used previously in studies on expression and regulation [33]. While positively correlated gene expression patterns indicate synergism, anti-correlated or uncorrelated expression patterns indicate antagonism between genes. In this study, a synergistic interaction was detected when the effect of a particular combination of genotype and activity levels on gene expression was more than the sum of the effects of genotype and activity levels considered independently. In other words, synergistic effects were identified when the expression of a gene under a combination of genotype and activity levels was more extreme than the average expression under each level separately. Likewise, an antagonistic interaction was detected when the effect of a particular combination of genotype and activity levels on gene expression was less than the sum of the effects of genotype and activity levels considered independently. In other words, antagonistic effects were detected when the expression of a gene under a combination of genotype and activity levels was less extreme than the average expression under each level separately. Distinct profiles including a minimum of 50 genes were further evaluated and functional categories enriched within cluster were investigated in DAVID.

**Presentation of findings.** Findings are presented and discussed in a sequence starting with genes exhibiting significant activity-by-genotype interaction, followed by genes exhibiting significant main effects of activity or genotype. Genes corresponding to transcripts exhibiting significant (FDR-adjusted P-value < 0.005; log2(fold change) > |1.3|) differential expression are identified in the Results section and their profiles are discussed in the Discussion sections. Broader lists of genes reaching a lower significance threshold are presented in the supplementary materials (in S1 File). Discussion focuses on gene expression patterns previously unreported in the context of the conditions studied and on patterns previously reported in similar or comparable studies.

## Results

### RNA-Seq analysis and organization of findings

Considering that myostatin inhibition and physical activity are being explored as treatment options for muscle degeneration and other disorders, it is important to understand the impact of these factors at the gene co-regulation level. The RNA-Seq profile analyses revealed changes in the transcriptome of a skeletal muscle, the triceps brachii muscle, between C57/BL6 wild-type and myostatin-reduced mice under two physical activity conditions. First, the quality and quantity of the sequence reads was evaluated across samples. The average size of the RNA-Seq FastQ file was 1.3 G bases/sample. The average quality score Phred of the reads along the 65 positions across all samples was 30. The number of reads and quality scores along the reads were comparable across samples from all four activity-by-genotype groups. Likewise, the percentage of reads mapped to the mouse genome was similar across samples and was on average 84.6% (17,486,782 of 20,675,801 total reads mapped). Of these, 5,013,631 (28.7%) had multiple alignments (12,183 had >20 alignments).

### Activity-by-genotype interaction effect on gene expression in triceps brachii muscles

Overall, 1,836 genes exhibited significant (FDR-adjusted P-value < 0.005) activity-by-genotype interaction. Table 1 lists genes exhibiting significant (FDR-adjusted P-value < 2XE-9) activity-by-genotype interaction effects due to space limitations. Twenty five of these genes had the maximum log2(fold change) > |1.3|. An extended list of differentially expressed genes with FDR-adjusted P-value <.01 is presented in Table A in S1 File). The most extreme average fold change among genes exhibiting significant interaction was observed for the contrasts IM-AW, followed by IW-IM and AM-IM. This result indicates that the expression of genes in the inactive myostatin-reduced mice tended to be most different than the other three activity-genotype groups and this profile is a driving factor on the identification of genes expression exhibiting significant interaction. Conversely, on average the less extreme fold change among genes exhibiting significant interaction was observed in the contrast IW-AM. This finding reveals that these two conditions do not exhibit a synergistic effect among the genes presenting significant interaction.

**Table 1:** Differentially expressed genes (FDR-adjusted P-value < 2XE-9) across activity-genotype contrasts.

Gene Name*	Log2Fold^1^						FDR-adjusted P-Value
	AM-AW^3^	AM-IM^4^	AW-IM^2^	IW-IM^7^	IW-AW^5^	IW-AM^6^	
*Mettl21e*	*2*.*72*	*0*.*57*	*3*.*29*	*4*.*48*	*-1*.*19*	*-3*.*91*	*1*.*55E-13*
Dusp18	-0.78	-0.73	-1.51	-0.64	-0.87	-0.09	1.55E-13
Per1	1.22	0.40	1.62	0.82	0.79	-0.42	1.55E-13
Atp1b2	0.62	0.34	0.96	0.31	0.65	0.023	1.55E-13
Tnnc1	-0.89	-4.14	-5.03	-3.67	-1.36	-0.47	1.55E-13
Zmynd17	-1.25	0.47	-0.78	-1.63	0.85	2.09	1.55E-13
Myh7	-0.72	-4.87	-5.58	-4.37	-1.20	-0.49	1.55E-13
Tpm3	-0.75	-2.22	-2.96	-1.76	-1.20	-0.46	1.55E-13
*Ddah1*	*-1*.*64*	*0*.*08*	*-1*.*57*	*-2*.*15*	*0*.*59*	*2*.*23*	*1*.*55E-13*
Myl2	-1.15	-3.74	-4.89	-3.49	-1.39	-0.25	1.55E-13
Atp2a2	-0.89	-2.15	-3.05	-2.31	-0.74	0.16	1.55E-13
*Pak1*	*0*.*56*	*-0*.*09*	*0*.*46*	*1*.*07*	*-0*.*60*	*-1*.*16*	*1*.*55E-13*
Tnnt1	-0.92	-4.07	-4.99	-3.66	-1.33	-0.41	1.55E-13
Csrp3	-0.91	-1.92	-2.83	-2.01	-0.82	0.09	1.55E-13
Fxyd6	-0.76	-2.05	-2.81	-1.96	-0.85	-0.08	1.55E-13
Myoz2	-0.59	-2.43	-3.03	-2.12	-0.91	-0.31	6.84E-13
Myl3	-0.62	-3.15	-3.77	-2.76	-1.01	-0.39	6.84E-13
Naca	0.87	0.03	0.91	0.43	0.48	-0.39	1.89E-12
Ak3	0.55	-1.53	-0.99	0.41	-1.40	-1.95	1.89E-12
Cyp1a1	2.04283	-0.84607	1.19676	0.267273	0.929775	-1.11319	3.16E-12
*Grem2*	*1*.*44986*	*0*.*213147*	*1*.*66277*	*2*.*52511*	*-0*.*86228*	*-2*.*31217*	*3*.*16E-12*
*Fos*	*-0*.*78073*	*0*.*039794*	*-0*.*74082*	*-1*.*52664*	*0*.*786004*	*1*.*56659*	*8*.*48E-12*
Gnb2l1	0.739797	-0.36408	0.375364	-0.32355	0.699348	-0.04009	8.56E-12
Wnk2	1.10552	0.056718	1.16275	0.760786	0.402344	-0.70346	2.45E-11
*Tmem100*	*-0*.*76578*	*0*.*050259*	*-0*.*71569*	*-1*.*11695*	*0*.*401146*	*1*.*16691*	*3*.*02E-11*
Ncor2	0.556591	-0.14625	0.41082	-0.02854	0.439626	-0.11714	6.96E-11
Acta2	0.972604	-0.62395	0.348594	-0.39031	0.738874	-0.23374	2.01E-10
*Pmepa1*	*-0*.*67603*	*-0*.*0258*	*-0*.*70168*	*-1*.*02573*	*0*.*324245*	*1*.*00005*	*2*.*01E-10*
*Sln*	*1*.*5134*	*0*.*156663*	*1*.*67004*	*2*.*36704*	*-0*.*69664*	*-2*.*21015*	*2*.*90E-10*
Dhcr24	0.771441	-0.05624	0.715401	-0.12054	0.83638	0.064728	3.20E-10
Dusp23	0.468437	0.024808	0.493519	0.026371	0.467286	-0.00141	1.01E-09
Ramp1	-0.66605	-0.05061	-0.71666	-0.92948	0.213069	0.87899	1.07E-09
Atrnl1	-0.67904	-0.04955	-0.72883	-0.49233	-0.2365	0.442585	1.32E-09
*Lancl1*	*-1*.*22412*	*0*.*08892*	*-1*.*13543*	*-1*.*33056*	*0*.*195232*	*1*.*41926*	*1*.*43E-09*

* Genes exhibiting significant synergistic and antagonist activity-by-genotype interaction effects are displayed in italics. Ddha1, Lancl1, Fos, Tmem1, and Pmepa1 follow a synergistic pattern; Sln, Grem2, Mettl2, and Pak1 follow an antagonistic pattern.

1. When considering two values, A and B, Log2Fold Change = Log2 (B/A). For example, Log2Fold of the contrast AW-IM = log2(IM/AW).

2. AW-IM refers to the active wild-type vs. inactive myostatin-reduced contrast group

3. AM-AW refers to the active myostatin-reduced vs. active wild-type contrast group

4. AM-IM refers to the active myostatin-reduced vs. inactive myostatin-reduced contrast group

5. IW-AW refers to the inactive wild-type vs. active wild-type contrast group

6. IW-AM refers to the inactive wild-type vs. active myostatin-reduced contrast group

7. IW-IM refers to the jnactive wild-type vs. inactive myostatin-reduced contrast group

*Expanded gene names, listed in alphabetical order: Acta2 = actin, alpha 2, smooth muscle, aorta; Ak3 = adenylate kinase 3; Atp1b2 = ATPase, Na+/K+ transporting, beta 2 polypeptide; Atp2a2 = ATPase, Ca++ transporting, cardiac muscle, slow twitch 2; Atrnl1 = Attractin-Like 1; Csrp3 = cysteine and glycine-rich protein 3; Cyp1a1 = Cytochrome P450, Family 1, Subfamily A, Polypeptide 1; Ddah1 = dimethylarginine dimethylaminohydrolase 1; Dhcr24 = 24-Dehydrocholesterol Reductase; Dusp18 = dual specificity phosphatase 18; Dusp23 = Dual Specificity Phosphatase 23; Fos = FBJ Murine Osteosarcoma Viral Oncogene Homolog; Fxyd6 = FXYD domain-containing ion transport regulator 6; Gnb2l1 = Guanine Nucleotide Binding Protein (G Protein), Beta Polypeptide 2-Like; Grem2 = Gremlin 2, DAN Family BMP Antagonist; Lancl1 = LanC Lantibiotic Synthetase Component C-Like 1; Mettl21e = methyltransferase like 21E; Myh7 = myosin, heavy chain 7, cardiac muscle, beta; Myl2 = myosin, light polypeptide 2, regulatory, cardiac, slow; Myl3 = myosin, light polypeptide 3; Myoz2 = myozenin 2; Naca = nascent polypeptide-associated complex alpha polypeptide; Ncor2 = Nuclear Receptor Corepressor 2; Pak1 = p21 protein (Cdc42/Rac)-activated kinase 1

Per1 = period circadian clock 1; Pmepa1 = Prostate Transmembrane Protein, Androgen Induced 1; Ramp1 = Receptor (G Protein-Coupled) Activity Modifying Protein 1; Sln = Sarcolipin; Tmem100 = Transmembrane Protein 100; Tnnc1 = troponin C, cardiac/slow skeletal; Tnnt1 = troponin T1, skeletal, slow; Tpm3 = tropomyosin 3, gamma; Wnk2 = WNK Lysine Deficient Protein Kinase 2; Zmynd17 = zinc finger, MYND-type containing 17

**Patterns of differential gene expression across activity-genotype contrasts.** The number of differentially expressed genes (P-value < 0.05) for AW-IM, AM-AW, AM-IM, IW-AW, IW-AM, and IW-IM was 1,051, 86, 711, 119, 238 and 390, respectively. Several interpretations can be made from the progression of number of differentially expressed genes starting with the highest number in AM-IM followed by AM-IM, followed by IW-IM followed by AM-AW. Firstly, activity level was associated with more differentially expressed genes in myostatin-reduced mice than in the wild-type mice. Also, genotype was associated with more differentially expressed genes in active relative to inactive mice. Among all activity-genotype combination groups, inactive myostatin-reduced mice exhibited the most number of differentially expressed genes relative to other activity-genotype combinations, including the highest number of over-expressed genes at FDR-adjusted P-value < 0.01**.** On the other hand, active wild-type mice exhibited the fewest number of differentially expressed genes relative to all other activity-genotype combinations, and these genes were overexpressed at FDR-adjusted P-value < 0.01.

Among active mice, the genotype difference was associated with the fewest number of differentially expressed genes among all pairwise contrasts. Likewise, among wild-type mice, activity level was associated with the second lowest number of differentially expressed genes among all pairwise contrasts. In contrast, changes in activity level elicited more differentially expressed genes in myostatin-reduced mice than in wild-type mice.

**Fig 1:**
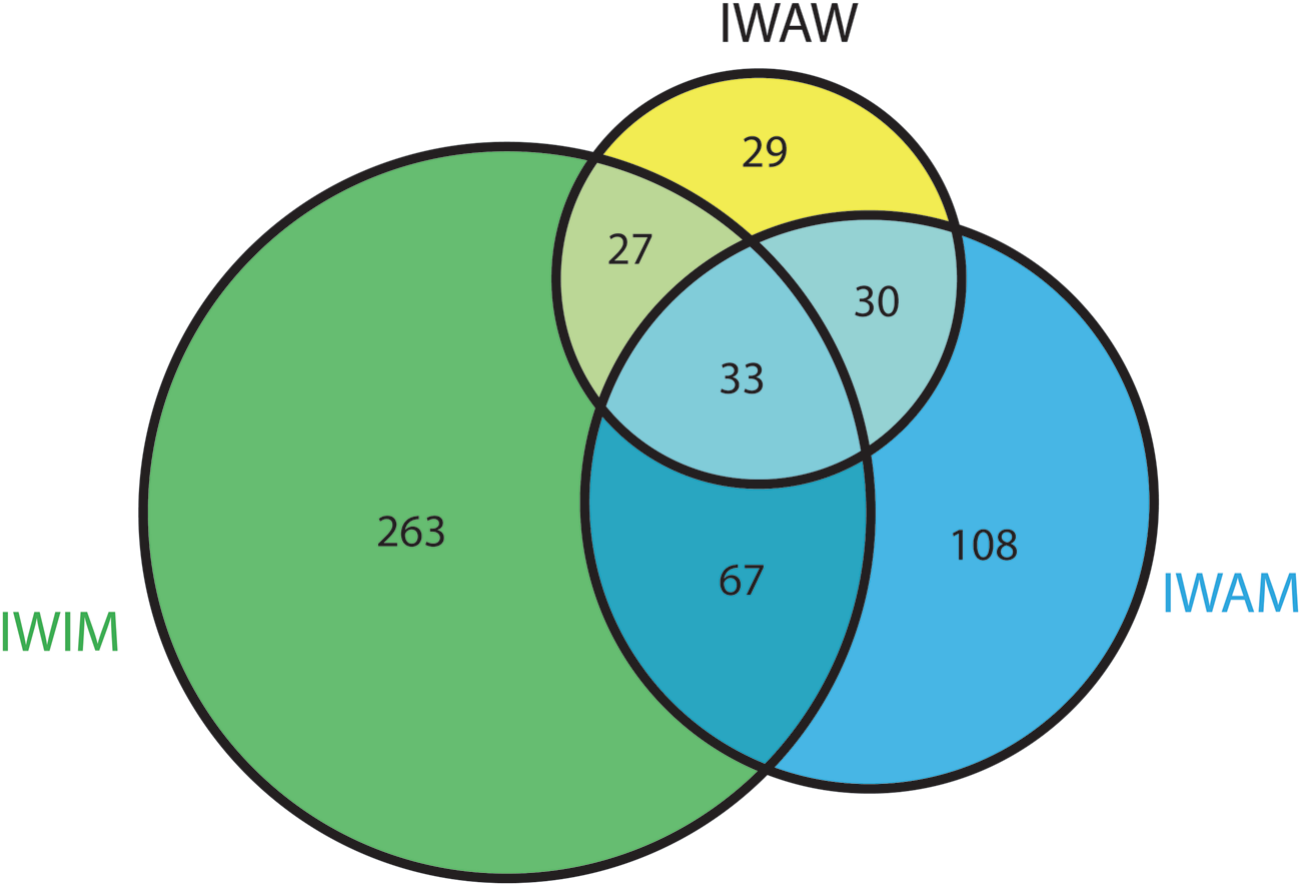
Number of differentially expressed genes that overlap between inactive wild-type (IW) and remainder activity-genotype groups: inactive myostatin-reduced (IM), active wild-type (AW), and active myostatin-reduced (AM).

Fig. 1 presents a Venn diagram illustrating shared differentially expressed genes between one set of three orthogonal contrasts including the IW baseline group. Of these genes, 33 genes were shared between all three contrasts including the IW baseline group (Table 2). These genes are highlighted because their expression in IW (inactive wild type) was significant different from all other three groups. These genes are of interest because either one or both conditions (genotype and activity) resulted in a departure from baseline conditions. The majority of the genes differentially expressed between IW-AM were shared with the contrasts IW-AW and IW-IM (130 out of 238). This same pattern was evident in the contrast of IW-AW to IW-AM and IW-IM (90 out of 119). In contrast, the majority of the genes differentially expressed in the contrast IW-IM were unique to this contrast (263).

**Table 2:** Shared differentially expressed genes (FDR-adjusted P-value <.01) between three orthogonal contrasts including the Inactive-Wild Type (IW) baseline group.

Gene Name*	Log2(Fold)^1^		
	IW-AW^2^	IW-AM^3^	IW-IM^4^
M6prbp1	-0.55	-0.92	0.47
Pak1	-0.60	-1.16	1.07
Casq2	-0.87	-1.25	-0.75
Fos	0.79	1.57	-1.53
Dhrs4	-0.63	-0.64	-0.60
Gm5514	-1.44	-1.21	-1.52
Ddah1	0.59	2.23	-2.15
Fabp3	-0.75	-0.63	-0.93
Got1	-0.62	-0.55	-0.44
2310076L09Rik	-0.87	-0.72	-1.04
Mafb	-0.78	-1.07	0.58
EG225594	-1.64	-1.55	2.22
4832428D23Rik	-1.20	-3.91	4.48
BDH1	-1.83	-1.47	-2.24
ZMYND11	0.85	2.10	-1.63
Gck	1.17	1.48	-0.70
Esrrb	-1.26	-1.28	-1.28
Acaa2	-0.59	-0.53	-0.69
Ankrd2	-1.18	-0.92	-1.59
Actn2	-0.62	-0.78	-0.87
Egr1	1.56	1.65	-1.48
Myom3	-1.13	-1.08	-1.93
9830123M21Rik	0.69	2.52	-0.49
Rn45s	-0.80	-1.94	1.16
NNT	-0.79	-0.72	-0.70
Dgat2	-0.83	-0.70	-0.97
H19	-0.68	-0.89	0.43
Tbc1d1	0.72	0.98	-0.91
IL15	-1.05	-1.39	1.06
Myh2	-1.08	-0.85	-1.78
COL22A1	0.63	0.74	-0.48
ORF63	0.85	0.93	-0.75
IDH2	-0.85	-0.67	-0.92
M6prbp1	-0.55	-0.92	0.47

1. When considering two values, A and B, Log2Fold Change = Log2 (B/A). For example, Log2Fold of the contrast AW-IM = log2(IM/AW).

5. IW-AW refers to the inactive wild-type vs. active wild-type contrast group

6. IW-AM refers to the inactive wild-type vs. active myostatin-reduced contrast group

7. IW-IM refers to the jnactive wild-type vs. inactive myostatin-reduced contrast group

*Expanded gene names, listed in alphabetical order: 2310076L09Rik = RIKEN cDNA 2310076L09 gene; 4832428D23Rik = RIKEN cDNA 4832428D23 gene; 9830123M21Rik = RIKEN cDNA 9830123M21 gene; Acaa2 = acetyl-Coenzyme A acyltransferase 2 (mitochondrial 3-oxoacyl-Coenzyme A thiolase); Actn2 = actinin alpha 2; Ankrd2 = ankyrin repeat domain 2 (stretch responsive muscle); BDH1 = 3-hydroxybutyrate dehydrogenase, type 1; Casq2 = calsequestrin 2; COL22A1 = collagen, type XXII, alpha 1; Ddah1 = dimethylarginine dimethylaminohydrolase 1; Dgat2 = diacylglycerol O-acyltransferase 2; Dhrs4 = dehydrogenase/reductase (SDR family) member 4; EG225594 = predicted gene 4841; Egr1 = early growth response 1; Esrrb = estrogen related receptor, beta; Fabp3 = fatty acid binding protein 3, muscle and heart; similar to mammary-derived growth inhibitor; Fos = FBJ osteosarcoma oncogene; Gck = glucokinase;Gm5514 = lactate dehydrogenase B; predicted gene 5514; Got1 = similar to Aspartate aminotransferase, cytoplasmic (Transaminase A) (Glutamate oxaloacetate transaminase 1); glutamate oxaloacetate transaminase 1, soluble; H19 = H19 fetal liver mRNA; IDH2 = Isocitrate Dehydrogenase 2 (NADP+), Mitochondrial; IL15 = interleukin 15; M6prbp1 = mannose-6-phosphate receptor binding protein 1; Mafb = v-maf musculoaponeurotic fibrosarcoma oncogene family, protein B (avian); Myh2 = myosin, heavy polypeptide 2, skeletal muscle, adult, myosin, heavy polypeptide 1, skeletal muscle, adult; Myom3 = myomesin family, member 3; Nnt = nicotinamide nucleotide transhydrogenase; ORF63 = open reading frame 63; Pak1 = p21 protein (Cdc42/Rac)-activated kinase 1; Rn45s = RNA, 45S Pre-Ribosomal 5; Tbc1d1 = TBC1 domain family, member 1; similar to TBC1 domain family member 1; ZMYND11 = zinc finger, MYND domain containing 11

**Functional enrichment analysis of activity-genotype contrasts.** Functional enrichment analysis was performed on genes exhibiting differential expression between pairs of activity-genotype combination groups (FDR-adjusted P-value < 0.01) for each of the six contrasts individually. Clusters of categories exhibiting enrichment scores > 3.0 (corresponding to average across categories within a cluster P-value < 0.01) were considered for discussion. These functional enrichment results can be found in Tables B to F in S1 File. The contrasts AM-IM (Table B in S1 File) and IW-AW (Table C in S1 File) shared the cardiac muscle contraction (mmu04260) KEGG pathway, indicating that changes in activity level are associated with differential expression of genes linked to the muscle contraction network, regardless of genotype. The differentially expressed genes in these contrasts also exhibit enrichment of the tricarboxylic acid cycle TCA cycle (mmu00020) KEGG pathway and a number of GO MF terms linked to metabolism of cofactors and vitamins that are also linked to these metabolic pathways.

Notably, some functional categories enriched among the genes differentially expressed in the IW-IM contrast (Table D in S1 File) were also enriched among genes differentially expressed in the AM-AW (Table E in S1 File). Amid these, the BP categories of muscle cell differentiation (GO:0042692), various muscle development terms, and the KEGG pathways hypertrophic and dilated cardiomyopathy (mmu05410 and mmu05414, respectively). Also, the BP vasculature development (GO:0001944) was enriched among the genes differentially expressed in the AM-IM (Table B in S1 File) and IW-IM (Table D in S1 File) contrasts.

**Genes exhibiting significant activity-by-genotype interaction.** Overall, 2,074 genes exhibited a significant activity-by-genotype interaction association with expression (FDR-adjusted P-value < 0.01) The genes exhibiting the most significant interaction effect including the log2 fold change between pairs of activity-by-genotype groups and the overall FDR-adjusted P-value are presented in Table 1. A more extensive list of genes is provided in Table A in S1 File). Several genes that exhibited a significant interaction also had one or more significant individual pairwise contrasts between activity-genotype combinations. Examples of these genes included: methyltransferase like 21E gene (Mettl21e); Tropomyosin 3 (Tpm3); Troponin T1 slow skeletal muscle (Tnnt1); nascent polypeptide-associated complex alpha polypeptide (Naca); dual specificity phosphatase 18 (Dusp18) and ATPase, Na+/K+ transporting, beta 2 polypeptide (Atp1b2), and the neuropeptide gene Proenkephalin-A (Penk; S1 File). The interaction pattern for Penk is revealed in the similar expression among inactive mice (regardless of genotype) yet over-expression in active relative to inactive myostatin-reduced mice and under-expression in active myostatin-reduced mice relative to inactive-wild type.

Other genes that exhibited a significant interaction did not reach high significance in particular contrasts; however, the integration of consistent borderline significant contrasts resulted in a significant overall interaction effect (Table 1 and Table A in S1 File). Examples of these genes included: ATPase, Na+/K+ Transporting, Beta 2 Polypeptide (Atp1b2), p21 protein (Cdc42/Rac)-activated kinase 1 (PAK1), and Nascent Polypeptide-Associated Complex Alpha Subunit (Naca). The interaction pattern of PAK1 is characterized by highest expression in active myostatin-reduced, followed by inactive-wild type, followed by inactive myostatin-reduced mice.

The interaction pattern of cysteine and glycine-rich protein 3 (CSRP3) and Myozenin 2 (Myoz2) were characterized by highest expression in inactive myostatin-reduced mice relative to all other activity level-genotype groups. The consistent interaction patterns of Myosin, light polypeptide 2 (Myl2) and myosin, light polypeptide 3 (Myl3) can be summarized by over-expression in inactive myostatin-reduced mice relative to all other activity level-genotype groups. The pathways of these Myosin genes could result in this consistent profile. Myosin light chains (Myls) modulate muscle contraction and may be involved in myogenesis or muscle regeneration [34–37].

**Clusters of expression profiles among genes exhibiting significant activity-by-genotype interaction.** Within the genes that exhibit significant interaction, various profiles of over- and under-expression were identified. However, the joint functional analysis of these groups may hinder or bias the discovery of enriched categories. Thus, discretizing the pairwise contrasts into over-, under- and non-differentially expressed identified clusters of genes exhibiting common expression profiles across the six contrasts. Profiles including more than 100 genes were subjected to functional enrichment analysis in DAVID. Table 3 lists the most common profiles of over- (denoted with a 1), under- (denoted with a-1) and not differentially expressed genes across the 6 pairwise contrasts considered and the number of genes in each profile cluster. The three most common profiles were: +100000 (183 genes), -100000 (146 genes), and-10–1000 (142 genes), where the first column in the series of +1, -1 and 0 denotes the AW-IM contrast and the third column denotes the AM-IM contrast. For example, +100000 represents a pattern of significant positive differential expression in the AM-IM contrast and non-significant (P-value > 0.0001) differential expression in all other contrasts.

**Table 3:** Most common profiles of over- (denoted with +1), under- (denoted with-1) and not (denoted with 0) differentially expressed (P-value < 0.0001) genes across the 6 pairwise contrasts.

Profile^1^						Number of Genes^2^
AM-AW	AM-IM	AW-IM	IW-IM	IW-AW	IW-AM	
0	0	0	0	0	0	1509
0	0	+1	0	0	0	183
0	0	-1	0	0	0	146
0	-1	-1	0	0	0	142
0	-1	0	0	0	0	86
0	+1	0	0	0	0	83
0	0	+1	+1	0	0	71
0	+1	+1	0	0	0	68
0	-1	-1	-1	0	0	67
0	+1	+1	+1	0	0	54

1. The six contrast groups are ordered as follows- active wild-type vs. inactive myostatin-reduced, active myostatin-reduced vs. active wild-type, active myostatin-reduced vs. jnactive myostatin-reduced, inactive wild-type vs. active wild-type, inactive wild-type vs. active myostatin-reduced, and jnactive wild-type vs. inactive myostatin-reduced. Each number of the 6 in the Profile “code” refers to each contrast in order (the first number in the Profile “code” denotes significance level for the first group, active wild-type vs. inactive myostatin-reduced, the second number denotes significance level for the second group, etc.)

2. Unlisted profiles include < 50 genes

Tables 4, 5, and 6 list the clusters of functional categories enriched for profiles +100000 and-100000 (enrichment score > 3.0), and-10–1000 (enrichment score > 2.0). Two of the differential expression profiles across the pairwise contrasts included over- or under-expression in the two most extreme activity-genotype contrasts studied IW-AM (categories +100000 and-100000). Considering that genes from a process or pathway can be impacted in opposite manner by the same activity-genotype combination, the genes in the previous two profile clusters were combined and functional analysis of this augmented cluster was also undertaken.

**Table 4:** Enriched (enrichment score > 3.0) clusters of Gene Ontology (GO) biological process (BP), molecular function (MF) Functional Annotation Tool (FAT) categories, and KEGG pathways among the genes exhibiting the profile characterized by under-expressed in active wild-type vs inactive myostatin-reduced and in active myostatin-reduced vs inactive myostatin-reduced and not differentially expressed in all other contrasts.

Category	Term	Number of Genes	P-Value	FDR-adjusted P-value
Score = 16.28				
KEGG PATHWAY	mmu00190:Oxidative phosphorylation	31	6.69E-33	6.84E-30
KEGG PATHWAY	mmu05012:Parkinson’s disease	30	6.01E-31	6.14E-28
GOTERM BP FAT	GO:0022900~electron transport chain	24	1.58E-27	2.39E-24
KEGG PATHWAY	mmu05010:Alzheimer’s disease	30	9.82E-27	1.00E-23
KEGG PATHWAY	mmu05016:Huntington’s disease	30	1.16E-26	1.19E-23
GOTERM BP FAT	GO:0006091~generation of precursor metabolites and energy	29	2.84E-25	4.30E-22
GOTERM BP FAT	GO:0055114~oxidation reduction	32	3.95E-17	5.99E-14
GOTERM MF FAT	GO:0015078~hydrogen ion transmembrane transporter activity	12	1.27E-11	1.65E-08
GOTERM MF FAT	GO:0015077~monovalent inorganic cation transmembrane transporter activity	12	2.47E-11	3.20E-08
GOTERM MF FAT	GO:0022890~inorganic cation transmembrane transporter activity	12	1.55E-09	2.01E-06
KEGG PATHWAY	mmu04260:Cardiac muscle contraction	10	2.24E-07	2.29E-04
GOTERM MF FAT	GO:0015002~heme-copper terminal oxidase activity	6	5.10E-07	6.61E-04
GOTERM MF FAT	GO:0016675~oxidoreductase activity, acting on heme group of donors	6	5.10E-07	6.61E-04
GOTERM MF FAT	GO:0016676~oxidoreductase activity, acting on heme group of donors, oxygen as acceptor	6	5.10E-07	6.61E-04
GOTERM MF FAT	GO:0004129~cytochrome-c oxidase activity	6	5.10E-07	6.61E-04

**Table 5:** Enriched (enrichment score > 3.0) clusters of Gene Ontology (GO) biological process (BP), molecular function (MF) Functional Annotation Tool (FAT) categories, and KEGG pathways among the genes under- or over-expressed in the active wild-type vs inactive myostatin-reduced contrast and not differentially expressed in all other contrasts.

Category	Term	Number of Genes	P-Value	FDR-adjusted P-value
Score = 5.07				
GOTERM BP FAT	GO:0006091~generation of precursor metabolites and energy	24	1.74E-10	2.86E-07
GOTERM BP FAT	GO:0022900~electron transport chain	15	8.68E-09	1.43E-05
GOTERM BP FAT	GO:0055114~oxidation reduction	32	6.91E-07	1.1E-03
GOTERM BP FAT	GO:0045333~cellular respiration	9	7.84E-06	0.01
GOTERM BP FAT	GO:0015980~energy derivation by oxidation of organic compounds	11	7.88E-06	0.01
GOTERM BP FAT	GO:0022904~respiratory electron transport chain	6	1.10E-04	0.18
GOTERM BP FAT	GO:0006119~oxidative phosphorylation	7	4.14E-04	0.68
Score = 4.18				
GOTERM BP FAT	GO:0006091~generation of precursor metabolites and energy	24	1.74E-10	2.86E-07
KEGG PATHWAY	mmu00190:Oxidative phosphorylation	16	7.12E-09	8.25E-06
GOTERM BP FAT	GO:0022900~electron transport chain	15	8.68E-09	1.43E-05
KEGG PATHWAY	mmu05016:Huntington’s disease	18	1.82E-08	2.11E-05
KEGG PATHWAY	mmu05012:Parkinson’s disease	15	7.73E-08	8.96E-05
KEGG PATHWAY	mmu05010:Alzheimer’s disease	16	6.59E-07	7.63E-04
GOTERM BP FAT	GO:0006119~oxidative phosphorylation	7	4.14E-04	0.68

**Table 6:** Enriched (enrichment score > 2.0) clusters of Gene Ontology (GO) biological process (BP), molecular function (MF) Functional Annotation Tool (FAT) categories, and KEGG pathways among the genes under- or over-expressed in the active myostatin-reduced vs inactive myostatin-reduced contrast and not differentially expressed in all other contrasts.

Category	Term	Number of Genes	P-Value	FDR-adjusted P-value
Score = 2.43				
GOTERM BP FAT	GO:0001525~angiogenesis	8	3.00E-04	0.48

Gene under-expression in active wild-type and active myostatin-reduced relative to inactive myostatin-reduced is a clear example of activity-by-genotype interaction because the combination of inactivity and myostatin-reduced genotypes was associated with higher gene expression than activity (regardless of genotype). Among genes sharing the first profile (under-expression in active wild-type and active myostatin-reduced relative to inactive myostatin-reduced) one highly enriched functional cluster (enrichment score = 16.28) was identified (Table 4). This cluster is comprised of 14 GO BP, MF, and KEGG pathway terms including the cardiac muscle pathway and pathways for inflammation-associated neurodegenerative conditions including Parkinson’s disease, Alzheimer’s disease, and Huntington’s disease.

Among the profile cluster characterized by genes under- or over-expressed in the AW-IM contrast and not differentially expressed in all other contrasts (Table 5), five functional clusters presented enrichment scores > 2.0. Functional categories in the two clusters with highest scores are discussed (Table 5). The first functional cluster (enrichment score = 5.06) contained 9 BP terms. Oxidation reduction and oxidative phosphorylation were again found significant in this list along with multiple electron transport terms including electron transport chain, respiratory electron transport chain, ATP synthesis coupled electron transport, and mitochondrial ATP synthesis coupled electron transport. Additionally, this list includes generation of precursor metabolites and energy, cellular respiration, and energy derivation by oxidation of organic compounds terms. The second functional cluster (enrichment score = 4.18) contained 15 GO BP, MF, and KEGG pathway terms. This cluster contained many of the same terms found significant in the previous contrast discussed, including oxidative phosphorylation, cardiac muscle contraction, and neurodegenerative diseases. A large number of electron transport chain, energy generation, and mitochondrial ATP synthesis terms appeared on this list as well, highlighting again the crucial electron-transfer role of mitochondrial-driven processes.

Among the profile characterized by genes under- or over-expressed in the AM-IM contrast and not differentially expressed in all other contrasts (Table 6), the functional cluster presenting an enrichment score > 2.0 is discussed. The first functional cluster (enrichment score = 2.43) contained 5 BP terms. These significant terms included: angiogenesis, blood vessel development, vasculature development, blood vessel morphogenesis, and tube development. These categories are consistent with the vascular development category previously identified.

### Main effect of myostatin genotype on gene expression in triceps brachii muscles

Table 7 lists the 13 genes differentially expressed (FDR-adjusted P-value < 0.005; log2(fold change) > |1.3|) between genotype groups excluding transcripts exhibiting significant interaction effects. An extended list of differentially expressed genes (FDR-adjusted P-value < 0.01) is presented in Table G in S1 File. In total, 238 genes were differentially expressed between genotypes at FDR-adjusted P-value < 0.005. The most significant differentially expressed genes (Table 7) had positive log2(fold change) estimates indicating over-expression of those genes in the myostatin-reduced relative to wild-type mice. For example, platelet-derived growth factor subunit B (Pdgfb; S1 File) is a neuropeptide gene over-expressed in myostatin-reduced relative to wild-type mice.

**Table 7:** Genes differentially expressed (FDR-adjusted P-Value < 0.005 and log2(fold change) > |1.3|) between myostatin-reduced and wild-type mice in the skeletal muscle.

Gene*	Log2 (Myostatin-reduced/Wild-type)	FDR-adjusted P-value
ERCC2	4.19	2.5E-03
DGCR8	4.09	2.5E-03
METTL21E	3.48	2.5E-03
GSPT1	2.38	2.5E-03
ACTC1	2.29	2.5E-03
GREM2	1.91	2.5E-03
SLN	1.89	2.5E-03
CDH4	1.88	2.5E-03
F830016B08RIK	1.87	2.5E-03
KATNAL2	1.74	2.5E-03
IL12A	1.47	2.5E-03
MYBPH	1.34	2.5E-03
VASH2	1.30	2.5E-03

*Expanded gene names, listed in alphabetical order: ACTC1 = actin, alpha, cardiac muscle 1; CDH4 = cadherin 4, type 1, R-cadherin; DGCR8 = DGCR8 microprocessor complex subunit; ERCC2 = Excision Repair Cross-Complementing Rodent Repair Deficiency, Complementation Group 2; F830016B08RIK = RIKEN cDNA F830016B08 gene; GREM2 = gremlin 2, DAN family BMP antagonist; GSPT1 = G1 to S phase transition 1; IL12A = interleukin 12A (natural killer cell stimulatory factor 1, cytotoxic lymphocyte maturation factor 1, p35); KATNAL2 = katanin p60 subunit A-like 2; METTL21E = methyltransferase like 21E; MYBPH = myosin binding protein H; SLN = sarcolipin; VASH2 = vasohibin 2

Clusters of functional categories enriched (enrichment score > 3.0) among the genes differentially expressed between genotypes (raw P-value < 0.00005 comparable to FDR-adjusted P-value < 0.005) are listed in Table 8. An extended list of clusters (enrichment score > 1.5) including gene membership is presented in Table H in S1 File.

**Table 8:** Enriched (enrichment score > 3.0) clusters of Gene Ontology (GO) biological process (BP), molecular function (MF) Functional Annotation Tool (FAT) categories, and KEGG pathways among the genes differentially expressed between myostatin-reduced and wild-control mice (FDR-adjusted P-value < 0.05).

Category	Term	Number of Genes	P-Value	FDR-adjusted P-value^1^
Score = 3.59				
KEGG PATHWAY	mmu05410:Hypertrophic cardiomyopathy (HCM)	10	7.69E-05	0.09
KEGG PATHWAY	mmu04260:Cardiac muscle contraction	9	2.65E-04	0.31
KEGG PATHWAY	mmu05414:Dilated cardiomyopathy	9	8.16E-04	0.96
Score = 3.43				
GO BP FAT	GO:0007167~enzyme linked receptor protein signaling pathway	20	7.05E-06	0.01
GO BP	GO:0007179~transforming growth factor beta receptor signaling pathway	7	5.38E-04	0.89

^1^ False Discovery Rate adjusted P-value. Only terms with FDR-adjusted P-value < 0.99 or with more than 5 genes are listed

### Main effect of physical activity on gene expression in triceps brachii muscles

Table 9 lists the 21 genes differentially expressed (FDR-adjusted P-value < 0.005; log2(fold change) > |1.3|) between triceps brachii muscle from active and inactive mice excluding genes exhibiting significant interaction effects. An extended list of differentially expressed genes between activity levels (FDR-adjusted P-value < 0.01) is presented in Table I in S1 File. In total, 399 genes were differentially expressed at FDR-adjusted P-value < 0.005. The most significant differentially expressed genes (Table 9) had positive log2 (fold change) estimates indicating over-expression of those genes in active relative to inactive mice.

**Table 9:** Genes differentially expressed (FDR-adjusted P-Value < 0.005 and log2(fold change) > |1.3|) between active and inactive mice in the skeletal muscle.

Gene*	Log2(Active/Inactive)	FDR-adjusted P-value
ERCC2	3.95	1.54E-03
BDH1	2.38	1.54E-03
GM1078	2.28	1.54E-03
BC048679	2.28	1.54E-03
LRRC52	1.92	1.54E-03
LDHB	1.88	1.54E-03
TNNC1	1.86	1.54E-03
EGLN3	1.82	1.54E-03
MYL2	1.82	1.54E-03
TNNT1	1.78	1.54E-03
MYH7	1.78	1.54E-03
MYOM3	1.76	1.54E-03
ESRRB	1.74	1.54E-03
SLC26A10	1.65	1.54E-03
FHL2	1.65	1.54E-03
ANKRD2	1.63	1.54E-03
MYH2	1.61	1.54E-03
TM6SF1	1.59	1.54E-03
IQSEC2	1.57	1.54E-03
VAV2	1.53	1.54E-03
TPM3	1.51	1.54E-03

*Expanded gene names, listed in alphabetical order:ANKRD2 = ankyrin repeat domain 2 (stretch responsive muscle); BC048679 = cDNA sequence BC048679; BDH1 = 3-hydroxybutyrate dehydrogenase, type 1; EGLN3 = egl-9 family hypoxia-inducible factor 3; ERCC2 = excision repair cross-complementing rodent repair deficiency, complementation group 2; ESRRB = estrogen-related receptor beta; FHL2 = four and a half LIM domains 2; GM1078 = SH3 domain binding kinase family, member 3; IQSEC2 = IQ motif and Sec7 domain 2; LDHB = lactate dehydrogenase B; LRRC52 = leucine rich repeat containing 52; MYH2 = myosin, heavy chain 2, skeletal muscle, adult; MYH7 = myosin, heavy chain 7, cardiac muscle, beta; MYL2 = myosin, light chain 2, regulatory, cardiac, slow; MYOM3 = myomesin 3; SLC26A10 = solute carrier family 26, member 10; TM6SF1 = transmembrane 6 superfamily member 1; TNNC1 = troponin C type 1 (slow); TNNT1 = troponin T type 1 (skeletal, slow); TPM3 = tropomyosin 3; VAV2 = vav 2 guanine nucleotide exchange factor

Clusters of functional categories enriched (enrichment score > 3.0) among the genes differentially expressed between activity levels (raw P-value < 0.00005 or FDR-adjusted P-value < 0.005) are listed in Table 10. An extended list of clusters enriched among genes exhibiting significant activity effect (including gene membership) is presented in Table J in S1 File (enrichment score > 2.0).

**Table 10:** Enriched (enrichment score > 8.0) clusters of Gene Ontology (GO) biological process (BP), molecular function (MF) Functional Annotation Tool (FAT) categories, and KEGG pathways among the genes differentially expressed between active and inactive mice (FDR-adjusted P-value < 0.05).

Category	Term	Number of Genes	P-Value	FDR-adjusted P-value
Score = 19.79				
KEGG PATHWAY	mmu00190:Oxidative phosphorylation	51	5.29E-29	6.39E-26
KEGG PATHWAY	mmu05012:Parkinson’s disease	50	2.05E-27	2.47E-24
GOTERM BP FAT	GO:0006091~generation of precursor metabolites and energy	65	2.35E-27	4.12E-24
KEGG PATHWAY	mmu05010:Alzheimer’s disease	55	7.43E-25	8.98E-22
KEGG PATHWAY	mmu05016:Huntington’s disease	53	5.65E-23	6.82E-20
GOTERM BP FAT	GO:0022900~electron transport chain	39	4.30E-22	7.54E-19
GOTERM MF FAT	GO:0015078~hydrogen ion transmembrane transporter activity	23	4.07E-11	6.28E-08
GOTERM MF FAT	GO:0015077~monovalent inorganic cation transmembrane transporter activity	23	1.44E-10	2.22E-07
GOTERM MF FAT	GO:0022890~inorganic cation transmembrane transporter activity	26	2.46E-09	3.79E-06
Score = 8.42				
KEGG PATHWAY	mmu04260:Cardiac muscle contraction	28	1.13E-14	1.35E-11
GOTERM MF FAT	GO:0015078~hydrogen ion transmembrane transporter activity	23	4.07E-11	6.28E-08
GOTERM MF FAT	GO:0015077~monovalent inorganic cation transmembrane transporter activity	23	1.44E-10	2.22E-07
GOTERM MF FAT	GO:0022890~inorganic cation transmembrane transporter activity	26	2.46E-09	3.79E-06
GOTERM MF FAT	GO:0016675~oxidoreductase activity, acting on heme group of donors	10	7.32E-07	1.13E-03
GOTERM MF FAT	GO:0016676~oxidoreductase activity, acting on heme group of donors, oxygen as acceptor	10	7.32E-07	1.13E-03
GOTERM MF FAT	GO:0015002~heme-copper terminal oxidase activity	10	7.32E-07	1.13E-03
GOTERM MF FAT	GO:0004129~cytochrome-c oxidase activity	10	7.32E-07	1.13E-03

Both genotype and activity level were associated with significant changes in gene expression, irrespective of the remainder factor indicating main effects. The more extreme fold change estimates observed in the genotype relative to the activity contrasts (based on the top differentially expressed genes) indicate that the genotypes considered in this study have a higher impact on gene expression than the physical activity levels evaluated (Tables 7 and 9).

## Discussion

### Understanding activity-by-genotype interaction effect on gene expression in triceps brachii muscles

**Common expression profiles.** The patterns of differential gene expression across activity-genotype contrasts provided insight into activity-by-genotype interaction effect on gene expression in triceps brachii muscles. The identification of 33 genes shared between one set of the three orthogonal contrast groups including the baseline group set, inactive wild-type mice (IW-AM, IW-AW, and IW-IM) suggests a specific role of these genes in inactive wild-type mice that are sensitive to changes in the activity-genotype condition (Table 2). Meanwhile, the large percentage of non-overlapping genes in the contrast of inactive wild-type relative to both active and inactive myostatin-reduced offers a glimpse to the distinct impact of activity level on gene expression within myostatin-reduced mice.

Consideration of the number of differentially expressed genes across pairwise contrasts alone uncovered insightful interaction patterns that are cornerstone for more complex patterns across contrasts. Among all pairwise contrasts, myostatin-related genotype differences within the active group (contrast AM-AW) were associated with the fewest number of differentially expressed genes (86 genes), suggesting that activity may be picking up and modulating or compensating some expression regulated by myostatin. The second lowest number of differentially expressed genes (119 genes) was related to differences in activity level within wild-type mice (contrast IW-AW), indicating that activity alone was associated with more limited changes in gene expression than those observed in the combination of activity and myostatin reduction. Finally, considering the fact that activity level elicited more differentially expressed genes in myostatin-reduced than in wild-type mice along with the finding that the greatest number of differentially expressed genes is in the AW-IM contrast confirms the hypothesized synergistic impact of physical activity and the silencing of myostatin on gene expression.

**Functional enrichment within profiles.** Functional enrichment analysis of the activity-genotype contrasts offered insights into the impact of the activity-genotype combinations on GO MF and BP categories and KEGG pathways. Differentially expressed genes in the AM-IM and the IW-AW contrasts exhibited enrichment of the tricarboxylic acid cycle TCA cycle (mmu00020) KEGG pathway and a number of GO MF terms linked to metabolism of cofactors and vitamins that are also linked to these metabolic pathways. These enriched categories are consistent with the higher metabolism of active muscles; elevated amino acid and energy metabolism are seen in muscles of physically active mice, and presumably this elevated amino acid metabolism maintains the TCA cycle intermediates needed for fatty acid metabolism [38].

Muscle cell differentiation (GO:0042692) and muscle organ development (GO:0007517) were two BP terms enriched among the genes differentially expressed in the IW-IM and AM-AW contrasts. These categories are consistent with the known role of myostatin on cell differentiation and proliferation in triceps. Multiple studies have confirmed the direct impact of myostatin on these muscles. Specifically, myostatin-deficient mice have significantly larger tricep muscles than wild-type mice [39–41]. Myostatin depletion increased muscle mass by an average of 28%–44% in sedentary mice [39].

Enrichment of hypertrophic and dilated cardiomyopathy KEGG pathways (mmu05410 and mmu05414, respectively) was observed among genes differentially expressed in the IW-IM and AM-AW contrasts. Although hypertrophic cardiomyopathy is characterized by an hypertrophied heart muscle while tricep samples were used in this study, our results suggest that the expression of genes in similar biological processes are altered by myostatin genotype regardless of activity level. Our result is consistent with a previous report that a hypertrophic cardiomyopathy mutation is expressed in the messenger RNA of skeletal as well as cardiac muscle [42].

Finally, enrichment of the vasculature development BP (GO:0001944) among the genes differentially expressed in the AM-IM and IW-IM contrasts suggests that activity level within myostatin-reduced mice and myostatin genotype within inactive mice have comparable impact on the expression of genes in the vascular development pathway. While inactivity may have counteracted the effect of myostatin reduction in the former contrast, myostatin reduction may have counteracted the effect of inactivity in the latter contrast. Our results offer support at the gene expression level to claims that the processes that regulate blood vessel development can also enable the adult to adapt to changes in tissues that can be elicited by activity or pathologies [43].

**Notable genes.** Consideration of individual genes exhibiting significant activity-by-genotype interaction further augmented the understanding of the interplay between activity and myostatin genotype on the triceps brachii muscle transcriptome in C57/BL6 mice. PAK1 displayed consistent borderline significant differential expression across multiple contrasts, resulting in a significant overall interaction effect. A study of target genes of myostatin loss-of-function in muscles of bovine fetuses identified PAK1 [44]. The complex interplay between PAK proteins in regulation of signaling cascades controlling cell motility, proliferation, and morphology and reorganization of the cytoskeleton [45] may lead to compensatory mechanisms resulting in wild-type mice exhibiting higher levels of PAK1 than myostatin-reduced mixed within the inactive group. Supporting this hypothesis, signaling of PAK1 has been linked to the G1 to S phase transition of the cell cycle via regulation of cyclin D1 machinery [46]. This function is consistent with our findings of high levels of expression of MIR1945- G1 to S phase transition 1 (Gspt1) in our genotype contrast of myostatin-reduced vs. wild-type mice. Both genes are known to be associated with SMAD3 that in turn is associated with TGFB1. Similarly, myostatin negatively regulates the activation of satellite cells by controlling the G1 to S phase transition through down-regulation of Cdk2 and up-regulation of P21, the protein encoded by PAK1 [47]. The expression of PAK1 was highest in active myostatin-reduced mice, followed by inactive wild-type mice and then inactive myostatin-reduced mice. The detection of this gene further confirms the importance of G1 to S phase transition 1 in muscular physiology and the role of myostatin in inhibition. PAK1 appears to be activated during the process of vascular remodeling [48] and this is in agreement with the identification of enrichment of vascular development pathway in the IM-AW and IW-IM contrasts.

CSRP3 and Myoz2 shared the same interaction pattern of highest expression in inactive myostatin-reduced relative to all other activity level genotype groups. The parallel expression profiles of these two genes detected in the present study is in agreement with previous reports. The expression of CSRP3 and Myoz2 is high in skeletal muscles [49–50], positively regulating myogenesis through promotion of myogenic differentiation [51]. CSRP3 encodes the muscle LIM protein (MLP), a muscle specific protein expressed and located at the z-line [52] which has been described as essential for myogenesis given its potential for induction of myogenic differentiation [53]. Mice with a deficiency of this gene exhibit dilated cardiomyopathy [54]. Only a few proteins have been shown to interact directly with MLP: actin [55], alpha-actinin [56], beta-spectrin [57], and N-RAP [58]; A definitive link between myostatin and MLP has not been established. Other studies have found a relationship between expression of MLP and contractility [59–60]. In addition to playing structural and functional roles in skeletal muscle, MLP has been suggested to be a mediator of mechanical stress in cardiac tissue [61]. Muscle growth resulting from myostatin inactivation presumably creates an imbalance between the metabolic requirements of tissue cells and the previous perfusion capabilities of blood vessels, and CSRP3-encoded MLP may work to mediate this stress and reduce likelihood of cardiomyopathy.

Clusters of expression profiles among genes exhibiting significant activity-by-genotype interaction were identified as well. Among genes sharing the first profile (under-expression in active wild-type and active myostatin-reduced relative to inactive myostatin-reduced and similar expression levels across all other activity-genotype groups), KEGG pathways for several inflammation-associated neurodegenerative conditions including Parkinson’s disease, Alzheimer’s disease, and Huntington’s disease were enriched. Our results are in agreement with reports that myostatin causes sporadic inclusion body myositis (sIBM), a muscle-wasting disease that has pathogenesis similar to that of Alzheimer’s and Parkinson’s diseases [62]. Also, activin A protects from neural degeneration in individuals with Huntington’s disease, [63] and the relationship between myostatin and activin has been well established. Myostatin signals muscle mass control through activin receptors [64], meanwhile activin type IIB receptor acts as a myostatin inhibitor, causing a dramatic muscle mass increase [65]. In addition to the previous pathways, oxidation-reduction, oxidoreductase activity, and oxidase activity categories were also enriched among genes in the first profile. This enrichment is consistent with studies demonstrating that oxidative stress is often induced by physical activity due to the generation of reactive oxygen species (ROS) that occurs as skeletal muscles contract [66]. Also, myostatin acts as a pro-oxidant, inducing oxidative stress in skeletal muscle by inducing ROS [67]. In turn, this induces anti-oxidant enzymes in skeletal muscle through TNF-α and NADPH oxidase in a feed-forward manner [68]. Additional GO terms associated with the electron transport chain, such as generation of precursor metabolites and energy, monovalent inorganic transmembrane transporter activity, and inorganic cation transmembrane transporter activity, enriched in the first profile highlight the expected link between muscle function and mitochondria-dependent reformation of ATP through nutrient oxidation.

Additionally, oxidative phosphorylation, electron transport chain, energy generation, and mitochondrial ATP synthesis GO terms were enriched among the profile characterized by genes under- or over-expressed in the AW-IM contrast and not differentially expressed in all other contrasts. These findings are consistent with the electron transport chain, or the flow of electrons resulting from NADH and FADH2 oxidation, that establishes an electrochemical gradient vital in powering ATP synthesis in oxidative phosphorylation, the final stage of aerobic cell respiration. Myostatin reduction, although not affecting phosphorylated compound concentrations and intracellular pH at rest, causes up to a 206% increase in ATP cost of contraction as well as limiting the shift toward oxidative metabolism during muscle activity [69]. Muscle buildup caused by myostatin is sustained through a combination of reduced ATP synthesis and decreased protein degradation activity [13].

**Antagonistic and synergistic expression patterns.** Finally, using genes exhibiting significant (FDR-adjusted P-value < 2xE-12) genotype-by-activity interaction effects, antagonistic and synergistic expression patterns were identified. Synergistic effects occur when the expression of a gene under a combination of genotype and activity levels is more extreme than the average expression under each level separately. Antagonistic effects occur when the expression of a gene under a combination of genotype and activity levels is less extreme than the average expression under each level separately. An example of synergistic pattern would be when a gene that has high over-expression (e.g. 4 fold) in myostatin inactive relative to the average of all other groups meanwhile the expression in myostatin relative to wild type and the expression in inactive relative to active are less or non-significant (e.g. less than 2 fold). An example of antagonistic pattern would be a gene that is not or less differentially expressed (e.g. 1 fold) in myostatin inactive mice relative to the average of all other groups meanwhile the expression in myostatin relative to wild type and the expression in inactive relative to active are more significant or extreme (e.g. more than 3 fold).

Examples of synergistic or antagonistic mode of action of genotype and activity factors on gene expression are listed in Table 1. Mettl21e; Cytochrome P450, Family 1, Subfamily A, Polypeptide 1 (Cyp1a1); and Myelin protein zero (Mpz) were identified as antagonistic genes, where gene expression increases in one contrast and concurrently decreases in another contrast. Cyp1a1 and Mpz shared the same interaction pattern, characterized by lower expression in active wild-type mice relative to the inactive wild-type mice, and by higher expression in active myostatin-reduced relative to inactive myostatin-reduced mice. A striking example of antagonistic interaction among these significant genes is Mettl21e, which follows the opposite interaction pattern. The expression of Mettl21e in active wild-type mice is higher than that in inactive wild-type mice, and the expression in active myostatin-reduced mice is lower than in inactive myostatin-reduced mice. Similarly, Sarcolipin (Sln), Actin Alpha 2 (Acta2), nuclear receptor corepressor 2 (Ncor2), guanine nucleotide binding protein beta polypeptide 2-like 1 (Gnb2l1), and Gremlin 2 of the Cysteine Knot Superfamily (Grem2) displayed antagonistic interactions (Table 1 and Table A in S1 File).

The identification of significant interactions enabled the detection of synergistic effects between genotype and activity. For genes Naca, Dusp23, and Dhcr24, the difference in expression between myostatin genotype groups was more extreme than between activity groups (Table A in S1 File). The changes in gene expression between genotypes appeared to be magnified by activity. This suggests that the observed changes in transcript abundances identified in the activity-level contrast may be due to the sole effect of physical activity, while changes identified in the genotype contrast may be due to the effect of both physical activity and myostatin depletion. The similar expression levels between genotypes among inactive mice and striking differential expression between genotypes among the active mice indicates strong synergistic interplay. Other genes also exhibiting synergistic interaction between genotype and activity, included Dimethylarginine Dimethylaminohydrolase 1 (Ddah1), FBJ Murine Osteosarcoma Viral Oncogene Homolog (Fos), WNK Lysine Deficient Protein Kinase 2 (Wnk2), Transmembrane Protein 100 (Tmem100), Prostate Transmembrane Protein Androgen Induced 1 (Pmepa1), Receptor G Protein-Coupled Activity Modifying Protein 1 (Ramp1), LanC Lantibiotic Synthetase Component C-Like 1 (Lancl1), P21 Protein Cdc42/Rac-Activated Kinase 1 (Pak1), and Attractin-Like 1 (Atrnl1).

### Understanding the main effect of myostatin on gene expression in triceps brachii muscles

Among the genes differentially expressed (FDR-adjusted P-Value < 0.005 and log2(fold change) > |1.3|) between myostatin-reduced and wild-type mice (Table 7 and Table G in S1 File), the functions of three genes are associated to muscle physiology. The detection of Actin, alpha, cardiac muscle 1 (Actc1) in this study is consistent with reports that cardiac α-actin can functionally substitute at least in part for skeletal muscle α-actin in skeletal muscle [70]. Similarly, the detection of Sln differential expression between myostatin genotype groups is consistent with reports postulating that high Sln expression in human skeletal muscle is important to the physiology of the tissue [71]. Interestingly, Sln has recently been shown to mediate muscle-based thermogenesis [72]. Finally, differential expression of Grem2 has been detected in the quadricepts of a mouse model of non-dystrophic skeletal muscle congenital disease [73]. Also, Grem2 acts as an antagonist of bone morphogenetic proteins (BMPs) that influence the effectiveness of myostatin. Myostatin is synthesized as a precursor protein, which then becomes biologically active through BMP-driven proteolytic processing events [74–75]. The profile observed in this study can be explained by myostatin gene expression depletion requiring lower BMP convertase, which is achieved through the inhibitory action of Grem2.

Among the rest of the genes differentially expressed between genotype groups, four provided remarkable insight into myostatin’s effects on the gene networks of muscle development. The protein coded by the Piezo-type mechanosensitive ion channel component 1 (Piezo1) allows cells to react to physical stimuli. Mechanosensitive ion channels play a key role in the physiology of smooth muscle [71,76–77]. Consistent with the differential expression of XPD (also known as Excision repair cross-complementing rodent repair deficiency complementation group 2 or Ercc2) between genotype groups, a mutation on XP genes has been associated with a reduction in skeletal muscle in mice [78]. Similarly to the present study, Microprocessor complex subunit DGCR8 (Dgcr8) or DiGeorge syndrome critical region gene 8 has been linked to myoblast differentiation [79]. Finally, our results revealed the expression of gene G1 to S Phase Transition 1 (Gspt1), responsible for the G1 to S phase transition of the cell cycle [80]. Given the differential expression pattern of the present study, we propose that that myostatin could inhibit the process of myoblasts moving from the G1 to S phase of the cell cycle through up-regulation of p21 and subsequent inhibition of Cdk2 activity. An extended list (FDR-adjusted P-value < 0.01**)** of differentially expressed genes between myostatin-reduced and wild-type mice in triceps brachii muscles can be found in Table G in S1 File.

### Understanding the main effect of physical activity on gene expression in triceps brachii muscles

Many of the differentially expressed genes between active and inactive mice, unsurprisingly, are associated with the biological processes of contractile response of muscles to activity. A notable finding is that Ercc2 was differentially expressed between genotype groups and between activity groups as well, yet this gene did not exhibit a significant activity-by-genotype interaction effect. A similar molecular mechanism is speculated for both comparisons. Among other genes differentially expressed between activity groups, Tnnt1 [81] plays an essential role in skeletal muscle contraction by regulating calcium sensitivity [82]. The myoglobin protein, encoded by Mb, is present only in myocytes and oxidative skeletal muscle fibers. This gene is essential for oxygen storage in muscle [83], and facilitates oxygen diffusion by desaturating rapidly as muscle activity increases [84]. Cyp26b1 encodes protein Cytochrome P450 26B1, known to be present in adult mice skeletal muscle [85]. Cyp26b1 signals aortic smooth muscle cells through regulation of the metabolism of all-trans-retinoic acid, [86] which is crucial for regulation of gene expression, cell growth and differentiation [87]. Finally, the protein encoded by Tropomyosin alpha-3 (TPM3) is also essential for regulation of skeletal muscle contraction [88].

Novel associations between differentially expressed genes and activity level were also identified in this study. Many of these genes have indirect links to muscle function and activity, but the actual mechanism uncovered is unique and unexpected. The differentially expressed gene 3-hydroxybutyrate dehydrogenase (Bdh1) encodes an enzyme involved in the interconversion of acetoacetate and (R)-3-hydroxybutyrate, essential for fatty acid catabolism. Also, Bdh1 mRNA is found in all forms of muscle [89]. The role in catabolism could be associated with the need for energy that characterizes the skeletal muscle under activity. Regarding the enrichment of the ATP metabolic process, physical activity causes an increase in ATP cost of contraction [69]. At the same time, active mice have significant reduction in accumulation of body fat as compared to wild-type IQ motif Sec7 domain 2 (Iqsec2 or Brag2), which has been associated with myoblast cell-cell fusion [90]. This is notable because molecular components associated to cell-cell fusion are found both in myoblast and macrophage cells [90]. In addition, estrogen-related receptor beta (ERRbetta) is a nuclear receptor protein encoded by the Esrrβ gene that was differentially expressed among activity groups. This result is consistent with work demonstrating that ERRbeta/gamma agonist modulates GRalpha expression, and glucocorticoid responsive gene expression in skeletal muscle cells [91]. Finally, AK4 was differential expressed between activity groups and this gene is responsible for encoding adenylate kinase 4, an energy-mediating enzyme. This finding is in agreement with reports that AK4 is highly expressed in human skeletal muscle [92].

The enrichment of GO biological process terms related to vasculature development (angiogenesis, blood vessel development, vasculature development, blood vessel morphogenesis, and tube development) among the genes differentially expressed in the AM-IM and IW-IM contrasts suggests that the combination of activity and myostatin-reduced genotype has comparable impact to the combination of inactivity and wild-type typical myostatin genotype on the expression of genes in the vascular development pathway. A link between vascular development and muscle development is expected based on the logical physiological association of the two organ systems. Vasculature is modified in order to meet the metabolic requirements of tissue cells in response to changes in metabolic rate; oxygen is a major control element of this adaptation, as hypoxia initiates various signals which in turn lead to an increase in vessel growth [93]. Given this information, vascular growth and activation of vascular development pathways would be expected upon myostatin inactivation, as the resulting muscle growth presumably creates an imbalance between the metabolic requirements of tissue cells and the previous perfusion capabilities of blood vessels. In addition, genes activated during vascular processes, such as PAK1, were found through our analyses to exhibit significant activity-by-genotype interaction. The association between myostatin level and PAK1 was confirmed in a previous study of genes targeted by myostatin loss-of-function in bovine muscles [44].

## Conclusions

The study of the impact of physical activity and myostatin level on gene expression in the triceps brachii muscles of C57/BL6 mice uncovered novel and confirmed known associations at the gene and gene network levels. Novel and significant interaction effects were observed for some genes (e.g. Naca, Grem2) including synergistic effects (e.g. Naca, Dhcr24) and antagonistic effects (e.g. Mettl21e, Cyp1a1, Mpz). Functional analysis of genes presenting significant interaction effects uncovered novel (e.g. angiogenesis) and expected (e.g. oxidative phosphorylation, electron transport chain) enriched pathways and biological processes.

Among the genes exhibiting significant main genotype effect, known (e.g. Sln, Grem2) and novel (e.g. Piezo1, Ercc2, Gspt1) associations were detected. Functional analysis of genes presenting significant genotype effect uncovered novel (e.g. dilated and hypertrophic cardiomyopathy) and expected (e.g. muscle cell differentiation, muscle organ development) enriched pathways and biological processes. Likewise, among the genes exhibiting significant main activity effect, known (e.g. MB, Tpm and novel (e.g. Bdh1, Esrrβ) associations were detected. Functional analysis of genes presenting significant activity effect uncovered novel (e.g. Alzheimer’s, Parkinson’s and Huntington’s disease) and expected (e.g. oxidative phosphorylation, cardiac muscle contraction) enriched pathways and biological processes. While several genes and functional categories enriched among the differentially expressed genes uncovered in this study were consistent with previous reports, the identity and profile of the genes exhibiting the most extreme interaction and main genotype and activity effects opened new avenues of inquiry on the role of specific genes in skeletal muscle development and the effects of myostatin and physical activity on muscle function. The present study centered on the comparison of four genotype-activity groups based on transcriptome information from a specific skeletal muscle type, mouse strain, gender, and age. Consideration of additional muscle types, genotypes, activities, ages, and genders would help identify additional synergistic and antagonistic relationships between these factors.

The findings from the present study could have medical implications on preventive practices and therapies associated with muscle atrophy in humans and companion animal species and genome-enabled selection practices applied to food-production animal species. The study of changes in gene expression in response to myostatin gene expression level in skeletal muscle tissue involved genes that code for a number of proteins that are feedback regulated by the myostatin molecule. The functions of the genes exhibiting differential expression between genotype groups are primarily regulatory. This functional category includes microRNA and Piezo proteins that make the list of the top 10 differentially expressed genes, side-by-side with Grem2 proteins that modulate the metalloprotein BMP-mediated cleavage of the myostatin propeptide. The role of genes regulated by microRNAs was unanticipated, especially because these genes seem to impact central functions such as the G1 to S phase transition of the cell cycle of myoblasts. The role of genes coding for Piezo proteins that make mechanosensitive ion channels, which in turn regulate cationic currents in the cells, was also remarkable and unanticipated, especially because of the consistent profile of the genes in this family. The study of changes in gene expression patterns in response to activity level revealed enrichment of genes that code structural proteins important for muscle function, including troponin, tropomyosin and myoglobin proteins. Activity was also associated with differential expression of genes important for fatty acid metabolism, some linked to type II diabetes and obesity and others to DNA-repair capacity, stem cell renewal, and various forms of cancer.

Our results provide evidence supporting the role of myostatin as a master regulator and the hypothesis that physical activity affect the expression of genes associated with homeostatic balance between storage of fat and muscle growth. Down-regulation of myostatin expression enables muscle growth at full expense of storage of fat, a condition that is hardwired at the regulatory level (e.g. through antagonists of metalloenzymes responsible for the myostatin activation). During activity, the changes in gene expression associated with balance between storage of fat and growth appears more instantaneous and subtle. This balance involves the regulation of metabolic pathways of fatty acid synthesis and does not impinge on oxidative phosphorylation pathways. The master regulatory functions of myostatin identified in this study should now be explored at the biochemical level to identify details of the regulatory networks, especially because of their potential to assist in the development of muscular disorders.

## Supporting Information

S1 File Supporting tables. Table A: Differentially expressed genes (FDR-adjusted P-value <.01) across activity-genotype contrasts.
Table B: Enriched (enrichment score > 3) clusters of Gene Ontology (GO) biological process (BP), molecular function (MF) Functional Annotation Tool (FAT) categories, and KEGG pathways among differentially expressed genes (FDR-adjusted P-value < 0.01) in the active myostatin-reduced vs jnactive myostatin-reduced contrast group.
Table C: Enriched (enrichment score > 2.0) clusters of Gene Ontology (GO) biological process (BP), molecular function (MF) Functional Annotation Tool (FAT) categories, and KEGG pathways among differentially expressed genes (FDR-adjusted P-value < 0.01) in the inactive wild-type vs active wild-type contrast group.
Table D: Enriched (enrichment score > 3) clusters of Gene Ontology (GO) biological process (BP), molecular function (MF) Functional Annotation Tool (FAT) categories, and KEGG pathways among differentially expressed genes (FDR-adjusted P-value < 0.01) in the inactive wild-type vs inactive myostatin-reduced contrast group.
Table E: Enriched (enrichment score > 3) clusters of Gene Ontology (GO) biological process (BP), molecular function (MF) Functional Annotation Tool (FAT) categories, and KEGG pathways among the genes differentially expressed between active and inactive mice (FDR-adjusted P-value < 0.05.
Table F: Enriched (enrichment score > 3) clusters of Gene Ontology (GO) biological process (BP), molecular function (MF) Functional Annotation Tool (FAT) categories, and KEGG pathways among differentially expressed genes (FDR-adjusted P-value < 0.01) in the active wild-type vs inactive myostatin-reduced contrast group.
Table G: Genes differentially expressed (FDR-adjusted P-value < 0.01) between myostatin-reduced and wild-type mice in triceps brachii muscle.
Table H: Enriched (enrichment score > 3) clusters of Gene Ontology (GO) biological process (BP), molecular function (MF) Functional Annotation Tool (FAT) categories, and KEGG pathways among differentially expressed genes (FDR-adjusted P-value < 0.005) between triceps brachii muscle of myostatin-reduced and wild-type mice.
Table I: Genes differentially expressed (FDR-adjusted P-value < 0.01) between active and sedentary mice in triceps brachii muscle.
Table J: Enriched (enrichment score > 2) clusters of Gene Ontology (GO) biological process (BP), molecular function (MF) Functional Annotation Tool (FAT) categories, and KEGG pathways among differentially expressed genes (FDR-adjusted P-value < 0.005) between triceps brachii muscle of active and sedentary mice.
(DOCX)
