# Supplementary material for: Synergistic and Antagonistic Interplay between Myostatin Gene Expression and Physical Activity Levels on Gene Expression Patterns in Triceps Brachii Muscles of C57/BL6 Mice
Source: PLoS One. 2015 Feb 24;10(2):e0116828. doi: 10.1371/journal.pone.0116828 (PMC4339580; doi:10.1371/journal.pone.0116828)
Supplement: S1 File — Table A: Differentially expressed genes (FDR-adjusted P-value <.01) across activity-genotype contrasts. Table B: Enriched (enrichment score > 3) clusters of Gene Ontology (GO) biological process (BP), molecular function (MF) Functional Annotation Tool (FAT) categories, and KEGG pathways among differentially expressed genes (FDR-adjusted P-value < 0.01) in the active myostatin-reduced vs jnactive myostatin-reduced contrast group. Table C: Enriched (enrichment score > 2.0) clusters of Gene Ontology (GO) biological process (BP), molecular function (MF) Functional Annotation Tool (FAT) categories, and KEGG pathways among differentially expressed genes (FDR-adjusted P-value < 0.01) in the inactive wild-type vs active wild-type contrast group. Table D: Enriched (enrichment score > 3) clusters of Gene Ontology (GO) biological process (BP), molecular function (MF) Functional Annotation Tool (FAT) categories, and KEGG pathways among differentially expressed genes (FDR-adjusted P-value < 0.01) in the inactive wild-type vs inactive myostatin-reduced contrast group. Table E: Enriched (enrichment score > 3) clusters of Gene Ontology (GO) biological process (BP), molecular function (MF) Functional Annotation Tool (FAT) categories, and KEGG pathways among the genes differentially expressed between active and inactive mice (FDR-adjusted P-value < 0.05. Table F: Enriched (enrichment score > 3) clusters of Gene Ontology (GO) biological process (BP), molecular function (MF) Functional Annotation Tool (FAT) categories, and KEGG pathways among differentially expressed genes (FDR-adjusted P-value < 0.01) in the active wild-type vs inactive myostatin-reduced contrast group. Table G: Genes differentially expressed (FDR-adjusted P-value < 0.01) between myostatin-reduced and wild-type mice in triceps brachii muscle. Table H: Enriched (enrichment score > 3) clusters of Gene Ontology (GO) biological process (BP), molecular function (MF) Functional Annotation Tool (FAT) categories, and KEGG pat [file pone.0116828.s001.docx]

**Table A. Differentially expressed genes (FDR-adjusted P-value < .01) across activity-genotype contrasts**

|  | **Log2Fold^1^** | | | | | | **FDR-adjusted P-Value** |
| --- | --- | --- | --- | --- | --- | --- | --- |
| **Gene Name*** | **AM-AW^3^** | **AM-IM^4^** | **AW-IM^2^** | **IW-IM^7^** | **IW-AW^5^** | **IW-AM^6^** |  |
| Myh7 | -0.715896 | -4.86754 | -5.58351 | -4.37461 | -1.20834 | -0.492349 | 1.55061E-13 |
| Mettl21e | 2.71757 | 0.572284 | 3.28946 | 4.48453 | -1.1954 | -3.91291 | 1.55061E-13 |
| Tnnc1 | -0.893475 | -4.13628 | -5.02991 | -3.66885 | -1.36077 | -0.467109 | 1.55061E-13 |
| Tnnt1 | -0.922226 | -4.07145 | -4.99398 | -3.66343 | -1.32996 | -0.407596 | 1.55061E-13 |
| Myl2 | -1.14521 | -3.74298 | -4.88828 | -3.49059 | -1.39755 | -0.252186 | 1.55061E-13 |
| Tpm3 | -0.745005 | -2.21626 | -2.9615 | -1.75991 | -1.20156 | -0.456519 | 1.55061E-13 |
| Atp2a2 | -0.899726 | -2.14883 | -3.04872 | -2.31009 | -0.738611 | 0.161206 | 1.55061E-13 |
| Csrp3 | -0.907975 | -1.92441 | -2.83231 | -2.0124 | -0.819791 | 0.0881133 | 1.55061E-13 |
| Fxyd6 | -0.760724 | -2.04843 | -2.80908 | -1.96326 | -0.845303 | -0.0848052 | 1.55061E-13 |
| Ddah1 | -1.64489 | 0.0800674 | -1.56503 | -2.15413 | 0.589166 | 2.23406 | 1.55061E-13 |
| Mss51 | -1.25011 | 0.470283 | -0.780012 | -1.62592 | 0.846016 | 2.0961 | 1.55061E-13 |
| Per1 | 1.21527 | 0.402372 | 1.61812 | 0.822605 | 0.795971 | -0.419627 | 1.55061E-13 |
| Dusp18 | -0.77636 | -0.732904 | -1.50945 | -0.643008 | -0.866315 | -0.0900057 | 1.55061E-13 |
| Pak1 | 0.555671 | -0.0904667 | 0.464841 | 1.06756 | -0.602787 | -1.15834 | 1.55061E-13 |
| Myl3 | -0.618511 | -3.15087 | -3.76929 | -2.75736 | -1.01168 | -0.393057 | 6.84093E-13 |
| Myoz2 | -0.596771 | -2.43484 | -3.03167 | -2.12488 | -0.90691 | -0.310092 | 6.84093E-13 |
| Ak4 | 0.545779 | -1.53163 | -0.9861 | 0.414543 | -1.40048 | -1.94637 | 1.89746E-12 |
| Naca | 0.874196 | 0.0330758 | 0.907418 | 0.427897 | 0.479745 | -0.394733 | 1.89746E-12 |
| Grem2 | 1.44986 | 0.213147 | 1.66277 | 2.52511 | -0.862282 | -2.31217 | 3.1566E-12 |
| Cyp1a1 | 2.04283 | -0.846068 | 1.19676 | 0.267273 | 0.929775 | -1.11319 | 3.1566E-12 |
| Ces1d | 0.455376 | 0.532124 | 0.987403 | 0.459335 | 0.528273 | 0.0728087 | 6.34341E-12 |
| Slc27a3 | 0.925562 | -0.194904 | 0.731343 | 1.42381 | -0.692129 | -1.61807 | 8.47991E-12 |
| Fos | -0.78073 | 0.0397939 | -0.740816 | -1.52664 | 0.786004 | 1.56659 | 8.47991E-12 |
| Trdn | 0.463262 | 0.423521 | 0.886414 | 0.0296331 | 0.856133 | 0.39306 | 1.58076E-11 |
| Il12a | 1.70077 | 0.551647 | 2.25226 | 1.32525 | 0.927111 | -0.773595 | 2.13486E-11 |
| Cyr61 | -0.956727 | 0.382286 | -0.574454 | -1.08462 | 0.510228 | 1.46699 | 2.44585E-11 |
| Wnk2 | 1.10552 | 0.0567183 | 1.16275 | 0.760786 | 0.402344 | -0.703462 | 2.44585E-11 |
| 1700001O22Rik | 0.842961 | 0.222087 | 1.06561 | 0.595321 | 0.470575 | -0.372726 | 2.44585E-11 |
| Tmem100 | -0.765781 | 0.050259 | -0.715694 | -1.11695 | 0.401146 | 1.16691 | 3.02369E-11 |
| St8sia4 | -0.529187 | -0.429698 | -0.959408 | -0.244969 | -0.714862 | -0.185517 | 3.23918E-11 |
| Mb | -0.504384 | -2.58944 | -3.09372 | -2.09249 | -1.00098 | -0.496674 | 3.49884E-11 |
| Pla2g12a | -0.377099 | -0.819614 | -1.19686 | -0.60273 | -0.594224 | -0.217047 | 3.85662E-11 |
| Tob2 | 0.512371 | 0.451731 | 0.9643 | 0.602603 | 0.361978 | -0.150646 | 3.85662E-11 |
| Gm7120 | 0.43366 | 0.524299 | 0.957789 | 0.225426 | 0.732458 | 0.298901 | 5.01265E-11 |
| Casq2 | 0.385092 | -2.00264 | -1.61783 | -0.751016 | -0.866788 | -1.25171 | 5.71885E-11 |
| Fndc5 | -0.360908 | -0.888147 | -1.24909 | -0.487968 | -0.760955 | -0.400188 | 5.78926E-11 |
| Atp1b1 | -0.490381 | -1.59653 | -2.08696 | -1.52722 | -0.559948 | -0.0695379 | 6.21906E-11 |
| Panx1 | -1.01462 | 0.279102 | -0.735482 | -1.17522 | 0.439802 | 1.45441 | 6.22859E-11 |
| Bdh1 | -0.359711 | -3.71899 | -4.07864 | -2.24487 | -1.83362 | -1.47396 | 7.52822E-11 |
| Rn45s | 1.13755 | -0.783728 | 0.354782 | 1.15936 | -0.804067 | -1.94193 | 8.14324E-11 |
| Ttyh2 | 0.716991 | 0.384094 | 1.10135 | 0.734185 | 0.367524 | -0.349722 | 9.49667E-11 |
| Mstn | -2.92049 | 0.668091 | -2.25296 | -2.56216 | 0.308867 | 3.22949 | 1.63371E-10 |
| Slc25a34 | -0.382647 | -2.23288 | -2.61522 | -1.73888 | -0.875975 | -0.493611 | 1.80164E-10 |
| Pmepa1 | -0.676026 | -0.0258012 | -0.701679 | -1.02573 | 0.324245 | 1.00005 | 2.01009E-10 |
| Acta2 | 0.972604 | -0.62395 | 0.348594 | -0.390314 | 0.738874 | -0.233739 | 2.01009E-10 |
| Cilp | -0.909292 | 0.355212 | -0.553914 | -0.839474 | 0.28574 | 1.19496 | 2.49142E-10 |
| Mt1 | -0.38949 | -0.591828 | -0.981266 | -0.0180637 | -0.96302 | -0.573534 | 2.87141E-10 |
| Sln | 1.5134 | 0.156663 | 1.67004 | 2.36704 | -0.696639 | -2.21015 | 2.90287E-10 |
| Ifi203 | -0.531174 | -0.474153 | -1.00571 | -0.281123 | -0.725002 | -0.193762 | 2.94038E-10 |
| Slc6a9 | 0.831254 | 0.250153 | 1.08194 | 0.547925 | 0.534472 | -0.297182 | 3.33468E-10 |
| Tnni1 | -0.88229 | -4.30219 | -5.18437 | -3.82164 | -1.36246 | -0.480147 | 3.36075E-10 |
| Esrrg | -0.389574 | -1.08989 | -1.47983 | -1.00362 | -0.476419 | -0.0867375 | 5.39004E-10 |
| BC048679 | 0.360712 | -2.82927 | -2.46857 | -0.662041 | -1.80628 | -2.16706 | 5.49847E-10 |
| Gdf11 | 0.739614 | 0.631221 | 1.3712 | 0.915573 | 0.455784 | -0.283912 | 5.93215E-10 |
| Ankrd2 | -0.265592 | -2.5071 | -2.77255 | -1.58833 | -1.18405 | -0.918469 | 7.82705E-10 |
| Lmcd1 | -0.335453 | -1.35131 | -1.68659 | -1.21386 | -0.472519 | -0.137162 | 9.90492E-10 |
| Klhl34 | -0.632542 | -1.72372 | -2.3562 | -2.03059 | -0.325334 | 0.307087 | 1.04215E-09 |
| Ramp1 | -0.666053 | -0.0506084 | -0.716657 | -0.929479 | 0.213069 | 0.87899 | 1.06716E-09 |
| Ldhb | -0.222724 | -2.73185 | -2.95468 | -1.51733 | -1.43703 | -1.21423 | 1.18488E-09 |
| Dkk3 | -2.5454 | 0.636021 | -1.90933 | -2.12484 | 0.215646 | 2.7609 | 1.26339E-09 |
| Igfn1 | -1.82714 | 2.02863 | 0.201991 | -0.49127 | 0.693449 | 2.52033 | 1.26339E-09 |
| Tpsb2 | -0.502745 | -0.959129 | -1.46157 | -0.37654 | -1.08452 | -0.582074 | 1.26339E-09 |
| Ntn4 | -0.32025 | -0.987585 | -1.30752 | -0.314415 | -0.992921 | -0.67272 | 1.27634E-09 |
| Lpl | -0.229583 | -1.04073 | -1.27055 | -0.619692 | -0.651137 | -0.421475 | 1.30504E-09 |
| Fam69b | -0.368628 | -1.74188 | -2.11034 | -0.892928 | -1.2169 | -0.848426 | 1.32359E-09 |
| Acot11 | -0.235128 | -0.893441 | -1.12842 | -0.490573 | -0.637499 | -0.402546 | 1.41961E-09 |
| Lancl1 | -1.22412 | 0.0889203 | -1.13543 | -1.33056 | 0.195232 | 1.41926 | 1.42692E-09 |
| Mybph | 1.05389 | 0.698107 | 1.75278 | 1.50607 | 0.246897 | -0.807333 | 1.74843E-09 |
| Mpz | 1.36966 | -1.11255 | 0.257157 | -0.914319 | 1.17164 | -0.198071 | 1.75035E-09 |
| Unc119 | 0.892117 | 0.113365 | 1.00608 | 0.684899 | 0.321478 | -0.570883 | 1.75355E-09 |
| Shmt1 | 0.759062 | 0.154052 | 0.913173 | 0.650136 | 0.263151 | -0.495957 | 1.76692E-09 |
| Khdrbs3 | -0.24673 | -1.89661 | -2.14326 | -1.29773 | -0.845662 | -0.598869 | 1.88844E-09 |
| Slc26a10 | 0.261632 | -2.2994 | -2.03764 | -0.843688 | -1.19354 | -1.45532 | 1.93161E-09 |
| Doc2g | -0.217763 | -0.703346 | -0.920693 | -0.0670946 | -0.853282 | -0.635756 | 1.93946E-09 |
| Hspa4l | -0.212766 | -0.930081 | -1.14344 | -0.360035 | -0.78366 | -0.570609 | 1.96069E-09 |
| Egln3 | 0.174029 | -2.06542 | -1.89144 | -0.289421 | -1.60193 | -1.77599 | 1.97255E-09 |
| Idh2 | -0.18077 | -1.58632 | -1.76693 | -0.917866 | -0.848825 | -0.668088 | 2.03566E-09 |
| Dbp | 0.542235 | 0.507796 | 1.0506 | 0.326271 | 0.724558 | 0.182141 | 2.03566E-09 |
| Cited2 | -0.181455 | -0.681137 | -0.862512 | -0.0882837 | -0.77429 | -0.592874 | 2.03795E-09 |
| Aldh18a1 | 1.02088 | -0.0397745 | 0.981213 | 0.707513 | 0.273895 | -0.747069 | 2.04017E-09 |
| Ociad2 | 0.653846 | 0.260727 | 0.91414 | 0.741522 | 0.172743 | -0.48107 | 2.04017E-09 |
| Myh2 | -0.231258 | -2.62379 | -2.85517 | -1.77721 | -1.07788 | -0.846408 | 2.07328E-09 |
| Lars2 | 1.0215 | -0.80187 | 0.219735 | 0.730966 | -0.511039 | -1.53269 | 2.07328E-09 |
| Nudt7 | -0.191994 | -0.863274 | -1.05533 | -0.445468 | -0.609762 | -0.417844 | 2.07328E-09 |
| Mcf2l | 1.05325 | -0.474647 | 0.578608 | 0.25431 | 0.32445 | -0.72883 | 2.07328E-09 |
| Atp13a5 | 0.578668 | 0.521054 | 1.09958 | 0.473497 | 0.626126 | 0.0474731 | 2.10768E-09 |
| Smtnl1 | -0.213559 | -1.04514 | -1.2586 | -0.714847 | -0.54368 | -0.330103 | 2.14179E-09 |
| Cish | 0.191654 | -1.12641 | -0.934233 | -0.0176965 | -0.916374 | -1.10835 | 2.30662E-09 |
| Igfbp5 | 0.859672 | 0.239939 | 1.09969 | 1.26742 | -0.167664 | -1.02748 | 2.41071E-09 |
| Tm6sf1 | 0.197842 | -1.81073 | -1.61331 | -0.266769 | -1.34684 | -1.54461 | 2.65371E-09 |
| Actn2 | 0.159452 | -1.65062 | -1.49114 | -0.87373 | -0.617356 | -0.776652 | 2.78017E-09 |
| G0s2 | -0.486917 | -0.465927 | -0.952481 | -0.0200916 | -0.932099 | -0.445286 | 3.14498E-09 |
| Casc4 | -0.227615 | -0.724318 | -0.952249 | -0.266089 | -0.686604 | -0.458941 | 3.19981E-09 |
| Tiam1 | -0.170534 | 0.880138 | 0.709431 | -0.132578 | 0.842038 | 1.01264 | 3.20756E-09 |
| Tmem37 | -0.571188 | -0.152709 | -0.723881 | -0.855149 | 0.131771 | 0.702839 | 3.27385E-09 |
| Dgat2 | -0.133548 | -1.6707 | -1.80409 | -0.968845 | -0.834932 | -0.701581 | 3.27653E-09 |
| Cpeb4 | -0.341854 | 0.915644 | 0.573509 | 0.104076 | 0.469395 | 0.81129 | 3.27653E-09 |
| Plin5 | -0.145363 | -1.76146 | -1.90656 | -1.03719 | -0.868886 | -0.723744 | 3.44319E-09 |
| Fabp3 | -0.126821 | -1.5533 | -1.68004 | -0.926527 | -0.753467 | -0.626625 | 3.44319E-09 |
| Rgs2 | -0.168931 | -0.907656 | -1.07687 | 0.03947 | -1.11654 | -0.947584 | 3.54306E-09 |
| Cd36 | -0.120542 | -1.04245 | -1.16358 | -0.58506 | -0.578948 | -0.458284 | 3.77281E-09 |
| Mdh1 | -0.122873 | -0.914515 | -1.03785 | -0.343856 | -0.694165 | -0.571054 | 3.7888E-09 |
| Tnfaip2 | 0.563205 | -0.323111 | 0.240256 | 0.688907 | -0.448388 | -1.01172 | 3.93859E-09 |
| Mylk4 | -0.125939 | 0.854401 | 0.728069 | 0.0668604 | 0.66127 | 0.787177 | 3.96292E-09 |
| Iqsec2 | 0.170062 | -1.60202 | -1.43184 | 0.138077 | -1.56961 | -1.73992 | 4.14894E-09 |
| Rrad | -0.936199 | 0.150053 | -0.785849 | -0.639926 | -0.145758 | 0.790375 | 4.21756E-09 |
| F830016B08Rik | 1.22791 | 1.04745 | 2.27433 | 2.41949 | -0.145541 | -1.37326 | 4.41979E-09 |
| Vopp1 | -0.224706 | -1.01753 | -1.24243 | -0.487288 | -0.755072 | -0.530419 | 4.41979E-09 |
| Pank1 | -0.373166 | -0.690833 | -1.06426 | -0.57337 | -0.491116 | -0.117894 | 4.41979E-09 |
| Mib1 | -0.0936048 | 0.869702 | 0.775783 | 0.202889 | 0.572882 | 0.666593 | 4.78415E-09 |
| Fabp4 | -0.0871893 | -0.774367 | -0.861601 | -0.325559 | -0.536107 | -0.44878 | 4.89517E-09 |
| Plekhb2 | -0.481142 | -0.477631 | -0.958905 | -0.731072 | -0.22758 | 0.253422 | 4.90811E-09 |
| Lmod2 | -0.449582 | -1.41935 | -1.86912 | -1.56425 | -0.304894 | 0.144745 | 5.47542E-09 |
| Nnt | -0.0768678 | -1.41214 | -1.48916 | -0.695951 | -0.793153 | -0.716218 | 5.71648E-09 |
| Col24a1 | 0.304851 | 1.11627 | 1.42127 | 0.713824 | 0.707555 | 0.402541 | 5.71648E-09 |
| Magix | -0.620914 | -0.132922 | -0.753523 | -0.827201 | 0.073903 | 0.694535 | 5.71648E-09 |
| Hsd17b7 | 0.669545 | 0.771693 | 1.44081 | 1.19922 | 0.241609 | -0.42799 | 5.81318E-09 |
| Fabp5 | -0.79665 | -0.663814 | -1.4607 | -0.338191 | -1.12261 | -0.32587 | 5.81318E-09 |
| Katnal2 | 1.2383 | 0.949141 | 2.18737 | 2.07046 | 0.116868 | -1.1214 | 6.00694E-09 |
| Got1 | -0.0664144 | -0.986217 | -1.05263 | -0.435024 | -0.617507 | -0.551076 | 6.08464E-09 |
| Acaa2 | -0.0589171 | -1.22011 | -1.27908 | -0.688839 | -0.590084 | -0.531097 | 6.36094E-09 |
| Tmem86a | -0.755538 | -0.438166 | -1.19347 | -1.0192 | -0.173785 | 0.581455 | 6.72744E-09 |
| Myom3 | -0.0491812 | -3.00941 | -3.0584 | -1.92738 | -1.13082 | -1.08168 | 7.19763E-09 |
| Aldh2 | -0.0436545 | -1.08082 | -1.12414 | -0.612254 | -0.511646 | -0.468057 | 7.19763E-09 |
| Irx5 | 0.838353 | 0.168417 | 1.00709 | 0.243384 | 0.763686 | -0.0746266 | 7.52413E-09 |
| Gpt | -0.0331721 | -0.861798 | -0.89468 | -0.245276 | -0.649108 | -0.616014 | 7.98074E-09 |
| Cyp26b1 | 1.60694 | -2.37783 | -0.770833 | -0.733835 | -0.0362907 | -1.64349 | 8.18547E-09 |
| Arrdc2 | 1.13577 | 0.307522 | 1.44353 | 1.41436 | 0.0295222 | -1.10637 | 8.18547E-09 |
| Ckmt2 | -0.0303527 | -1.13624 | -1.16656 | -0.280624 | -0.885854 | -0.855455 | 8.18547E-09 |
| Fmo2 | -0.0353279 | -0.856921 | -0.892495 | -0.119211 | -0.773464 | -0.738155 | 8.18547E-09 |
| Esrrb | 0.0246113 | -2.56507 | -2.54018 | -1.28253 | -1.25736 | -1.28217 | 8.54117E-09 |
| Cobll1 | -0.0170189 | -0.845592 | -0.862783 | 0.0775604 | -0.940516 | -0.923429 | 8.98512E-09 |
| Actc1 | 2.17748 | 0.231645 | 2.40914 | 2.4209 | -0.0115611 | -2.18902 | 9.13761E-09 |
| Car4 | 0.592546 | -1.43691 | -0.844253 | -0.140059 | -0.704055 | -1.29668 | 9.14314E-09 |
| Kif5c | 0.734011 | 0.369332 | 1.103 | 1.0248 | 0.0781352 | -0.655762 | 1.42115E-08 |
| Aldh1l2 | 0.52055 | 0.515153 | 1.03557 | 0.56274 | 0.472867 | -0.0476899 | 1.5246E-08 |
| Ephx2 | -0.15602 | -0.825203 | -0.981327 | -0.480055 | -0.501217 | -0.345185 | 1.5385E-08 |
| Fgf1 | -0.439034 | -1.13783 | -1.57693 | -1.27028 | -0.30667 | 0.132262 | 1.73549E-08 |
| Abcb1a | -0.179594 | -0.735899 | -0.915689 | -0.349487 | -0.566332 | -0.386651 | 1.93336E-08 |
| Dhrs4 | 0.0111254 | -1.2367 | -1.22563 | -0.599057 | -0.62626 | -0.637363 | 2.05121E-08 |
| Alpl | 0.0668634 | -1.4121 | -1.34499 | -0.756609 | -0.588128 | -0.655116 | 2.18213E-08 |
| Ybx2 | 0.410803 | -1.26384 | -0.852301 | -0.305458 | -0.546567 | -0.957617 | 2.18213E-08 |
| Xdh | 0.488572 | -0.853964 | -0.365397 | 0.0933089 | -0.458571 | -0.947175 | 2.36526E-08 |
| Arrdc3 | 0.0418716 | 0.880379 | 0.921743 | 0.381348 | 0.540097 | 0.498365 | 2.55099E-08 |
| Reep6 | 0.987788 | -0.110772 | 0.877641 | 1.06059 | -0.182723 | -1.17076 | 2.67187E-08 |
| P2ry1 | -0.0993728 | -0.859779 | -0.959689 | -0.366006 | -0.593851 | -0.494376 | 2.91364E-08 |
| Dusp26 | -0.545073 | -0.887276 | -1.43208 | -1.45131 | 0.0195649 | 0.564475 | 2.96295E-08 |
| Rhpn2 | 0.746243 | 0.668153 | 1.4144 | 0.929654 | 0.484809 | -0.261466 | 2.96295E-08 |
| Ankrd1 | -1.44536 | 0.730767 | -0.714856 | -0.646338 | -0.0686617 | 1.37686 | 3.20544E-08 |
| Lrtm1 | 0.465842 | 0.487765 | 0.953237 | 0.727039 | 0.226164 | -0.23972 | 3.97605E-08 |
| Casc1 | -0.174853 | -1.89446 | -2.06927 | -1.19898 | -0.870222 | -0.695383 | 4.05339E-08 |
| Gamt | 0.57789 | 0.457957 | 1.03625 | 0.778743 | 0.257818 | -0.320297 | 4.05817E-08 |
| Ddt | 0.120754 | -0.643628 | -0.522803 | 0.22579 | -0.748486 | -0.869183 | 4.13982E-08 |
| Cmbl | 0.488393 | 0.398773 | 0.887028 | 0.78951 | 0.0975415 | -0.390791 | 4.43243E-08 |
| Fam65b | 0.568487 | 0.590124 | 1.15852 | 1.06705 | 0.0916457 | -0.47681 | 4.53813E-08 |
| Sipa1l1 | 0.590065 | 0.381986 | 0.972086 | 0.877892 | 0.0942681 | -0.495865 | 5.47375E-08 |
| Gdap1 | 0.436676 | 0.482363 | 0.918501 | 0.742504 | 0.175783 | -0.260789 | 5.47375E-08 |
| Ankrd39 | -0.537118 | -0.294885 | -0.831497 | -0.964101 | 0.132876 | 0.669569 | 5.63065E-08 |
| Car2 | 0.0463307 | -0.82825 | -0.781907 | -0.179807 | -0.602057 | -0.648456 | 5.64463E-08 |
| Bmp6 | 0.554658 | -1.339 | -0.784161 | -0.498199 | -0.285652 | -0.840408 | 6.01227E-08 |
| Acadl | -0.22464 | -1.10721 | -1.33214 | -0.939788 | -0.392642 | -0.167836 | 6.01227E-08 |
| Gadd45g | 0.47966 | 0.300637 | 0.780439 | 0.860655 | -0.0800572 | -0.559667 | 6.09006E-08 |
| Cntnap2 | 0.536078 | 1.02671 | 1.56239 | 0.804808 | 0.757474 | 0.221376 | 6.16217E-08 |
| Wdyhv1 | -0.353354 | -1.03995 | -1.39327 | -0.478777 | -0.914434 | -0.561285 | 6.45115E-08 |
| Enho | 0.752274 | -0.260953 | 0.492099 | 0.881865 | -0.389392 | -1.14203 | 6.68767E-08 |
| Timeless | -0.189223 | -1.13962 | -1.32884 | -0.629754 | -0.698968 | -0.509733 | 7.0877E-08 |
| Mcpt4 | -0.647918 | -0.08924 | -0.737304 | 0.262612 | -0.999949 | -0.351995 | 7.40666E-08 |
| Evc | -0.363577 | -0.929901 | -1.29341 | -0.996605 | -0.296505 | 0.0669934 | 7.45769E-08 |
| Gimap4 | -0.059759 | -0.985165 | -1.04524 | -0.26148 | -0.783937 | -0.724055 | 7.87059E-08 |
| Otud1 | -0.154599 | -0.584794 | -0.739303 | -1.22992 | 0.490495 | 0.645133 | 8.06256E-08 |
| Tap1 | -0.152789 | -0.901061 | -1.05364 | -0.270225 | -0.783144 | -0.630584 | 8.67138E-08 |
| Crip3 | 0.635862 | 0.118763 | 0.754708 | 1.15478 | -0.399627 | -1.03567 | 9.10855E-08 |
| Zfp703 | 0.0425831 | -0.867341 | -0.824222 | -0.318514 | -0.505363 | -0.548243 | 1.00197E-07 |
| Cma1 | -0.7504 | -0.473709 | -1.2242 | -0.33676 | -0.887326 | -0.136939 | 1.04845E-07 |
| Mettl11b | -0.687808 | 0.376948 | -0.311145 | -0.790931 | 0.479757 | 1.16763 | 1.05946E-07 |
| Slc25a25 | 0.380599 | 1.434 | 1.81487 | 1.36504 | 0.450148 | 0.0693536 | 1.07779E-07 |
| Atp5g1 | 0.0147274 | -0.861255 | -0.846354 | -0.387201 | -0.458692 | -0.47371 | 1.20263E-07 |
| Tceal7 | -0.121484 | 0.579367 | 0.457693 | -0.373953 | 0.831285 | 0.952852 | 1.24845E-07 |
| Gstm7 | 0.235523 | -1.98978 | -1.75428 | -0.827463 | -0.926569 | -1.16214 | 1.29796E-07 |
| Tspan12 | 0.0554918 | -1.26148 | -1.2065 | -0.582545 | -0.624184 | -0.679579 | 1.40534E-07 |
| Piezo1 | 0.571703 | 0.370884 | 0.943029 | 0.892207 | 0.0512196 | -0.520693 | 1.41987E-07 |
| Stap2 | 0.620119 | 0.242392 | 0.862689 | 0.688959 | 0.174003 | -0.446265 | 1.42759E-07 |
| Lrtm2 | 0.393493 | 0.183285 | 0.576971 | -0.596405 | 1.17383 | 0.78006 | 1.47719E-07 |
| Emcn | 0.178304 | -0.8918 | -0.713581 | -0.197702 | -0.516023 | -0.694288 | 1.50569E-07 |
| Ecscr | -0.180237 | -0.803321 | -0.983643 | -0.285226 | -0.698216 | -0.517993 | 1.57773E-07 |
| Ctgf | -0.450168 | -0.431432 | -0.881673 | -1.18612 | 0.304535 | 0.754697 | 1.61807E-07 |
| Homer2 | -0.0874622 | -1.00449 | -1.09229 | -0.622484 | -0.469874 | -0.382484 | 1.70829E-07 |
| Myh1 | 0.12395 | -2.25695 | -2.13306 | -1.47828 | -0.654814 | -0.778625 | 2.11649E-07 |
| Syp | -0.516048 | -0.559904 | -1.07581 | -0.390075 | -0.685414 | -0.169516 | 2.19616E-07 |
| Btg2 | 0.119431 | -0.588652 | -0.469257 | -1.09128 | 0.622085 | 0.502419 | 2.26344E-07 |
| Tie1 | 0.251034 | -1.05641 | -0.805181 | -0.457793 | -0.347049 | -0.598194 | 2.29071E-07 |
| Socs2 | -0.570877 | -0.325715 | -0.896712 | -0.713363 | -0.1835 | 0.387334 | 2.43684E-07 |
| Ppp1r3c | 0.459155 | 0.398003 | 0.856699 | 0.930247 | -0.0734365 | -0.532508 | 2.48577E-07 |
| Trpc3 | 0.0540595 | -0.877727 | -0.823788 | -0.131481 | -0.692267 | -0.746226 | 2.76619E-07 |
| Stard5 | -0.954113 | 0.167253 | -0.786954 | -0.220596 | -0.566161 | 0.387741 | 2.96977E-07 |
| Pkp4 | 0.137413 | -0.854446 | -0.717027 | -0.206097 | -0.510874 | -0.648316 | 3.3738E-07 |
| Adck3 | -0.00740069 | -1.00752 | -1.01487 | -0.535588 | -0.479036 | -0.471747 | 3.67805E-07 |
| Slc25a22 | -0.0833946 | -1.05311 | -1.13621 | -0.657481 | -0.47821 | -0.395119 | 4.10681E-07 |
| Trp53i11 | 0.396217 | -1.18161 | -0.785135 | -0.477482 | -0.307112 | -0.703629 | 4.21353E-07 |
| Gck | -0.314104 | 0.78785 | 0.473906 | -0.696881 | 1.17097 | 1.48497 | 4.68399E-07 |
| Cntfr | 0.882935 | -0.51992 | 0.36391 | 0.702201 | -0.33788 | -1.22128 | 4.90078E-07 |
| Map1b | 0.355512 | 0.666495 | 1.02182 | 0.777534 | 0.244187 | -0.111224 | 5.25824E-07 |
| Gm5091 | 1.1009 | -0.0256131 | 1.07504 | 0.514241 | 0.5612 | -0.539862 | 5.30554E-07 |
| Ttc9 | -0.409773 | -0.803183 | -1.21283 | -0.821841 | -0.390899 | 0.0187372 | 5.77002E-07 |
| Lrrcc1 | -0.981685 | 0.263097 | -0.719121 | -0.778583 | 0.0589884 | 1.04092 | 6.11842E-07 |
| Itga4 | 0.475032 | 0.559739 | 1.03408 | 0.856942 | 0.176916 | -0.297998 | 6.30662E-07 |
| Fhl1 | -0.251496 | -1.43406 | -1.6858 | -1.34847 | -0.33724 | -0.0857773 | 7.34477E-07 |
| Golga7b | 0.584455 | 0.348965 | 0.933621 | 0.846571 | 0.0873426 | -0.497262 | 7.52829E-07 |
| Tpx2 | -0.600314 | 0.876668 | 0.276032 | -0.588853 | 0.864798 | 1.46524 | 7.54407E-07 |
| Fgl2 | -0.747174 | -0.393997 | -1.14143 | -0.793219 | -0.348557 | 0.39866 | 7.84691E-07 |
| Prima1 | -1.2737 | -0.00309295 | -1.27614 | -1.95682 | 0.681157 | 1.95447 | 8.60355E-07 |
| Arap3 | 0.0141659 | -0.862523 | -0.848185 | -0.418944 | -0.428956 | -0.443257 | 9.02199E-07 |
| Lims2 | 0.554275 | -1.02176 | -0.467371 | -0.28913 | -0.177893 | -0.732239 | 9.87385E-07 |
| Klf10 | 0.266025 | 0.721193 | 0.986993 | 0.667867 | 0.319212 | 0.053142 | 1.02182E-06 |
| Mthfd2 | 0.793122 | -0.265038 | 0.52781 | 0.774522 | -0.246708 | -1.03968 | 1.0573E-06 |
| AI506816 | 0.182653 | 0.721702 | 0.904559 | 0.320308 | 0.584307 | 0.401442 | 1.07088E-06 |
| Pdlim1 | -0.0971207 | -1.20699 | -1.30401 | -0.835331 | -0.468549 | -0.371449 | 1.07166E-06 |
| 2610035D17Rik | -0.382876 | -0.693568 | -1.07657 | -0.732824 | -0.3436 | 0.0392185 | 1.11241E-06 |
| Cuzd1 | 0.433095 | 0.622889 | 1.05579 | 0.651832 | 0.40394 | -0.0290812 | 1.41403E-06 |
| Ctnnal1 | -0.469946 | -0.443711 | -0.913907 | -0.745949 | -0.168132 | 0.301899 | 1.49661E-06 |
| Rgs4 | -0.0253055 | -1.05542 | -1.08111 | -0.578459 | -0.502755 | -0.477371 | 1.69871E-06 |
| Tnfsf10 | 0.472053 | 0.484749 | 0.956299 | 0.858592 | 0.0977372 | -0.374222 | 1.70415E-06 |
| Srd5a3 | -0.861639 | -0.110185 | -0.971883 | -0.570186 | -0.401484 | 0.459912 | 1.80813E-06 |
| Sh2d4a | 0.150767 | -0.601795 | -0.451316 | 0.378757 | -0.83012 | -0.980785 | 1.86389E-06 |
| Psmb8 | -0.198163 | -0.709067 | -0.906981 | -0.228515 | -0.678192 | -0.480159 | 2.17297E-06 |
| Hspb7 | -0.226096 | -0.988468 | -1.21423 | -0.961664 | -0.252218 | -0.0264015 | 2.18226E-06 |
| Vgll2 | -0.276385 | -1.14143 | -1.41756 | -1.2088 | -0.208432 | 0.0678076 | 2.19872E-06 |
| Nipa1 | 0.630191 | 0.620618 | 1.25054 | 1.27193 | -0.0211853 | -0.651395 | 2.21668E-06 |
| Dkk2 | -0.503497 | -0.545274 | -1.04922 | -0.828904 | -0.220495 | 0.283153 | 2.21668E-06 |
| Mafb | 0.290448 | -0.488491 | -0.197878 | 0.584929 | -0.782791 | -1.07332 | 2.26735E-06 |
| Tbc1d1 | -0.259597 | 0.0739017 | -0.185822 | -0.909405 | 0.723645 | 0.983288 | 2.2717E-06 |
| Ccnyl1 | -0.519853 | -0.107572 | -0.627886 | -0.833396 | 0.205357 | 0.725316 | 2.29989E-06 |
| 3110057O12Rik | 0.0274499 | -1.11904 | -1.09239 | -0.518592 | -0.574192 | -0.601394 | 2.35076E-06 |
| Podxl | 0.350811 | -1.01874 | -0.667909 | -0.639301 | -0.0284518 | -0.379385 | 2.37713E-06 |
| Adig | 0.0478722 | -1.39884 | -1.35093 | -0.752909 | -0.597669 | -0.645674 | 2.43041E-06 |
| Sema3b | -0.318992 | -0.826859 | -1.14571 | -0.7596 | -0.385804 | -0.0669268 | 2.47268E-06 |
| Ptpn3 | -0.329857 | -0.743337 | -1.07338 | -1.12871 | 0.0553305 | 0.385184 | 2.96687E-06 |
| Ercc2 | 5.30306 | -4.88721 | 0.415533 | 0.0832008 | 0.332801 | -4.9702 | 3.25381E-06 |
| Metrnl | -0.263106 | -0.756762 | -1.01989 | -0.390656 | -0.628882 | -0.365924 | 3.74342E-06 |
| Eid2 | -0.843 | 0.0957362 | -0.746705 | -0.508308 | -0.238374 | 0.604556 | 4.08375E-06 |
| Fam19a5 | -0.557291 | -0.625894 | -1.18316 | -1.17719 | -0.00567138 | 0.551458 | 4.17634E-06 |
| Myh11 | 0.784735 | -0.916579 | -0.131814 | -0.463612 | 0.331779 | -0.452933 | 4.44986E-06 |
| Snhg7 | -2.00456 | 3.25904 | 1.25337 | 0.934257 | 0.320811 | 2.3253 | 4.46612E-06 |
| Anks1 | 0.202285 | -0.926915 | -0.724379 | -0.42851 | -0.295538 | -0.498008 | 4.4797E-06 |
| Abcb1b | -0.135519 | -0.960279 | -1.09595 | -0.668492 | -0.42753 | -0.291915 | 5.67299E-06 |
| Nos3 | 0.390001 | -1.00127 | -0.610936 | -0.426683 | -0.183945 | -0.57415 | 7.75622E-06 |
| Stk26 | 0.430228 | 0.658684 | 1.08819 | 1.32918 | -0.241371 | -0.671254 | 8.33508E-06 |
| Aacs | 0.760336 | -0.0891088 | 0.6715 | 0.693145 | -0.0211857 | -0.781693 | 9.30558E-06 |
| Dach2 | -0.0277199 | -1.25274 | -1.28122 | -0.810517 | -0.471311 | -0.443367 | 9.51702E-06 |
| Ube2t | 0.134081 | -0.85662 | -0.722731 | 0.169249 | -0.892053 | -1.02617 | 1.08985E-05 |
| Ccdc85a | 0.191537 | -1.04174 | -0.85047 | -0.631978 | -0.218633 | -0.410141 | 1.20336E-05 |
| Cd200 | -0.294087 | -1.38309 | -1.67728 | -0.985002 | -0.692141 | -0.398161 | 1.28239E-05 |
| Srgn | 0.0789141 | -0.94109 | -0.862467 | -0.520985 | -0.341637 | -0.420462 | 1.32905E-05 |
| Gmnn | -0.0564416 | -0.831205 | -0.888045 | -0.344858 | -0.543388 | -0.486728 | 1.35823E-05 |
| Gm4841 | -0.087802 | 0.666281 | 0.578002 | 2.21825 | -1.64037 | -1.55254 | 1.38354E-05 |
| Ralb | -0.151852 | -0.946447 | -1.09828 | -0.777825 | -0.320354 | -0.168578 | 1.45343E-05 |
| Paqr4 | 0.192464 | -1.14407 | -0.951366 | -0.428947 | -0.522061 | -0.714791 | 1.47232E-05 |
| Gfod1 | 0.227532 | -1.22737 | -0.999745 | -0.706653 | -0.292919 | -0.520586 | 1.48154E-05 |
| Casr | 0.200969 | 0.982077 | 1.18315 | 0.922548 | 0.260778 | 0.0597631 | 1.51292E-05 |
| Gfra4 | 0.901153 | 0.0111015 | 0.912777 | 0.507233 | 0.405824 | -0.495602 | 1.56748E-05 |
| Ptprk | -0.207076 | -0.892896 | -1.10024 | -0.734692 | -0.365695 | -0.158518 | 1.61659E-05 |
| 9530091C08Rik | 0.209499 | 0.838777 | 1.04793 | 0.78203 | 0.265934 | 0.0563387 | 1.74294E-05 |
| Smarca1 | 0.0760268 | 0.641885 | 0.717153 | 0.998683 | -0.281983 | -0.357669 | 2.07617E-05 |
| Hmgb3 | 0.266415 | -0.878296 | -0.612286 | 0.966298 | -1.57873 | -1.84496 | 2.12767E-05 |
| Il15 | 0.345417 | -0.33318 | 0.0119095 | 1.05902 | -1.04708 | -1.39248 | 2.1961E-05 |
| Fdxr | -0.0782864 | -0.656632 | -0.734574 | 0.123776 | -0.857971 | -0.779899 | 2.26701E-05 |
| Flt4 | 0.0902847 | -0.980998 | -0.890477 | -0.564292 | -0.32594 | -0.416351 | 2.73752E-05 |
| Ddit4 | 0.362366 | 1.29717 | 1.65956 | 1.74495 | -0.0849261 | -0.447408 | 2.99766E-05 |
| Dapp1 | -0.339289 | -0.833262 | -1.17276 | -1.19184 | 0.0189398 | 0.358265 | 3.05276E-05 |
| Lrrn1 | -0.190653 | -1.38813 | -1.57904 | -1.41819 | -0.160887 | 0.0298383 | 3.24577E-05 |
| Rnd3 | -0.298466 | -0.89242 | -1.19134 | -1.10775 | -0.0838524 | 0.21481 | 3.94996E-05 |
| Me2 | -0.0775607 | -0.422391 | -0.500239 | 0.436434 | -0.936768 | -0.859103 | 3.94996E-05 |
| Fam179a | -0.0860038 | -1.4428 | -1.52864 | -1.21415 | -0.314145 | -0.228243 | 4.01996E-05 |
| Kcnab1 | 0.274645 | 1.08551 | 1.35976 | 1.20675 | 0.152778 | -0.121772 | 4.10863E-05 |
| Gm4861 | 0.389737 | 0.605452 | 0.994908 | 0.649286 | 0.34554 | -0.0441874 | 4.25731E-05 |
| Atf3 | 0.116629 | -1.30683 | -1.19025 | -1.43716 | 0.246946 | 0.130344 | 4.59803E-05 |
| Gm2382 | -0.56927 | -0.442729 | -1.0122 | -0.307321 | -0.704851 | -0.13541 | 4.75796E-05 |
| Dok5 | -0.418115 | -0.770787 | -1.18904 | -1.30164 | 0.11276 | 0.53093 | 6.77253E-05 |
| Ddx5 | 0.714562 | -0.875943 | -0.161385 | -0.624318 | 0.462929 | -0.251632 | 7.30536E-05 |
| Idnk | -0.00456432 | -0.785822 | -0.79053 | -0.0396848 | -0.750859 | -0.746334 | 7.70416E-05 |
| Smad7 | -0.298843 | -0.253243 | -0.55183 | -0.954922 | 0.403292 | 0.702023 | 8.12969E-05 |
| Pkn3 | 0.369179 | -1.48722 | -1.11788 | -0.944688 | -0.172894 | -0.542224 | 8.33831E-05 |
| Clec10a | 0.286039 | 0.668631 | 0.954685 | 0.845786 | 0.109116 | -0.177051 | 9.34276E-05 |
| Rassf9 | -0.215716 | -0.865994 | -1.08196 | -0.796416 | -0.285924 | -0.0700738 | 9.48085E-05 |
| Cdkl3 | -0.260337 | -0.473956 | -0.734523 | 0.109544 | -0.844407 | -0.583942 | 0.000101466 |
| 2310007L24Rik | 0.6398 | -0.148761 | 0.491275 | 0.723939 | -0.232333 | -0.872217 | 0.00010896 |
| Plod3 | 0.161523 | 1.63054 | 1.7925 | 1.64397 | 0.148675 | -0.0130473 | 0.000113548 |
| Pcdh1 | 0.600339 | -1.10051 | -0.499921 | -0.44582 | -0.0539523 | -0.654356 | 0.000115344 |
| Aldh1l1 | 0.522622 | -0.844977 | -0.322078 | 0.128954 | -0.450763 | -0.973499 | 0.000121326 |
| Prkg1 | -0.241777 | -0.725409 | -0.967765 | -0.932227 | -0.0358433 | 0.206132 | 0.000130141 |
| Sox18 | 0.233149 | -1.02238 | -0.788916 | -0.93143 | 0.142757 | -0.0904357 | 0.000139844 |
| A930003A15Rik | 0.534165 | 0.538192 | 1.07204 | 0.522552 | 0.549646 | 0.0154788 | 0.000155374 |
| 2310016D03Rik | -0.202821 | -0.869835 | -1.07292 | -1.05856 | -0.0143276 | 0.18832 | 0.00015615 |
| Barx2 | -0.105774 | -1.05153 | -1.15732 | -1.05297 | -0.104222 | 0.00153304 | 0.00019649 |
| Tmem132a | 0.140411 | -1.02828 | -0.887141 | -0.691189 | -0.195314 | -0.336127 | 0.000200927 |
| Zfp385b | -0.210898 | -1.366 | -1.57695 | -1.40192 | -0.175227 | 0.0355862 | 0.00022706 |
| Meg3 | 0.0786255 | 0.3034 | 0.382128 | -0.553726 | 0.936068 | 0.857276 | 0.00022971 |
| Stmn2 | 0.220128 | -1.34464 | -1.12469 | -0.692829 | -0.432033 | -0.652108 | 0.000230862 |
| Egr1 | -0.0876588 | 0.162913 | 0.0753636 | -1.48457 | 1.55993 | 1.64754 | 0.000238158 |
| Col7a1 | 0.636472 | 4.65728 | 5.29459 | 4.55634 | 0.738404 | 0.101577 | 0.000250084 |
| Inpp5j | 0.38361 | -1.3387 | -0.954797 | -1.02896 | 0.0746342 | -0.30923 | 0.00025728 |
| Frzb | 0.640077 | -0.82982 | -0.189922 | 0.212382 | -0.402301 | -1.04242 | 0.000268949 |
| Gas5 | -1.05184 | 0.959564 | -0.0925583 | -0.258581 | 0.166066 | 1.21789 | 0.000270323 |
| Nrtn | 0.379888 | -1.20944 | -0.828567 | -0.265749 | -0.562331 | -0.942439 | 0.00028066 |
| Ogdhl | -0.0814613 | -1.4155 | -1.49668 | -1.44741 | -0.049068 | 0.0323061 | 0.000337615 |
| Leprel4 | -0.0197324 | -1.03584 | -1.05518 | -0.771829 | -0.283187 | -0.26353 | 0.000390435 |
| Sfrp5 | 0.400464 | -1.3139 | -0.912938 | -1.22274 | 0.309994 | -0.0906367 | 0.000417048 |
| Cpa3 | -0.399719 | 0.0634791 | -0.336934 | 1.02199 | -1.35922 | -0.959261 | 0.000419739 |
| Socs4 | -0.888016 | 0.859886 | -0.02915 | 0.365835 | -0.395223 | 0.493102 | 0.000444015 |
| Mybpc1 | -0.0032148 | -1.20519 | -1.20857 | -1.16957 | -0.0391163 | -0.0357415 | 0.000461744 |
| Flt3l | 0.311617 | -1.28759 | -0.975767 | -0.471474 | -0.50398 | -0.815798 | 0.000476653 |
| 0610040B10Rik | -0.440783 | -1.27862 | -1.71972 | -0.797851 | -0.922021 | -0.481103 | 0.000522797 |
| Rtp4 | -0.369576 | -0.79554 | -1.16521 | -0.956778 | -0.208514 | 0.161094 | 0.000541665 |
| Atp2a3 | 0.675745 | -1.00266 | -0.326706 | -0.398296 | 0.0718807 | -0.603988 | 0.000545968 |
| Mapk11 | 0.478481 | 0.345027 | 0.823828 | 0.96236 | -0.138266 | -0.616942 | 0.000764459 |
| Hspa1a | -0.137804 | -0.925333 | -1.06279 | -1.18524 | 0.12258 | 0.26055 | 0.000802439 |
| Tspan33 | -0.131312 | -0.969695 | -1.10077 | -0.769104 | -0.331441 | -0.200299 | 0.000827017 |
| Slc9a1 | 4.4449 | -3.64085 | 0.814364 | -2.87833 | 3.68854 | -0.756329 | 0.000889467 |
| Casp6 | -0.232496 | -0.711665 | -0.944268 | -0.31701 | -0.627265 | -0.394722 | 0.000958729 |
| Zfp81 | -1.07178 | 0.755852 | -0.316452 | -0.0750257 | -0.241719 | 0.830305 | 0.000992777 |
| Morn4 | 0.123089 | 0.522072 | 0.645367 | 0.968314 | -0.322681 | -0.445993 | 0.001001793 |
| Il6ra | -0.446688 | -0.0323152 | -0.478897 | -0.925103 | 0.446288 | 0.892799 | 0.001083039 |
| BC028528 | -0.555666 | -1.21791 | -1.77361 | 0.0166636 | -1.79054 | -1.23485 | 0.001111067 |
| Hmga2-ps1 | -0.940264 | 0.347742 | -0.592656 | 0.546871 | -1.13935 | -0.199125 | 0.001123358 |
| Catsper4 | -0.389946 | 0.709352 | 0.319395 | -0.484639 | 0.80411 | 1.19413 | 0.001131638 |
| Pparg | 0.331256 | -1.18084 | -0.849754 | -0.378907 | -0.470907 | -0.802008 | 0.001245302 |
| Gata2 | 0.432061 | -0.983077 | -0.550746 | -0.42658 | -0.123851 | -0.556092 | 0.001322935 |
| Myc | 0.145905 | 0.814709 | 0.960764 | 0.679025 | 0.2818 | 0.135877 | 0.001390785 |
| AI118078 | 0.134462 | 0.387896 | 0.522533 | 1.182 | -0.659131 | -0.793743 | 0.001607506 |
| St6galnac2 | 0.1923 | -1.08436 | -0.891999 | -0.676827 | -0.215142 | -0.407478 | 0.001624258 |
| Fam65a | -0.205803 | 0.183036 | -0.0228962 | 1.66305 | -1.68562 | -1.47985 | 0.001638475 |
| Dpf1 | 0.352006 | -0.368027 | -0.0157145 | 0.900502 | -0.915959 | -1.26816 | 0.00169969 |
| Gimap1 | -0.00877092 | -0.999122 | -1.00778 | -0.417753 | -0.5898 | -0.581096 | 0.001914754 |
| Higd1b | 0.439243 | -1.24796 | -0.808559 | -0.299431 | -0.50903 | -0.948381 | 0.001915183 |
| Tph1 | 0.669659 | -0.148374 | 0.520765 | 1.91827 | -1.39763 | -2.06695 | 0.002195442 |
| En1 | 0.0136511 | -0.996993 | -0.983126 | -0.973017 | -0.0101332 | -0.0237755 | 0.002387029 |
| Isg15 | -0.936086 | 0.066322 | -0.869668 | -0.547428 | -0.322123 | 0.613898 | 0.00272791 |
| Zfpm1 | 0.754763 | -0.667445 | 0.0879884 | 0.385651 | -0.297415 | -1.05232 | 0.002845635 |
| Penk | -0.457446 | 0.881543 | 0.42404 | -0.021476 | 0.44538 | 0.903048 | 0.002875051 |
| Npl | 0.91212 | -0.35566 | 0.556266 | 0.326546 | 0.229698 | -0.682392 | 0.002987081 |
| Ccl24 | -0.902658 | 0.913194 | 0.0103644 | 0.597925 | -0.587286 | 0.315274 | 0.003018676 |
| Snhg3 | -0.534683 | 1.01558 | 0.480556 | 0.136933 | 0.343475 | 0.878245 | 0.003339804 |
| Slc15a2 | 0.246509 | -0.588728 | -0.342229 | 0.315812 | -0.658066 | -0.904572 | 0.003741899 |
| Vsig2 | 0.0382464 | -0.936535 | -0.897691 | -0.2886 | -0.608738 | -0.647423 | 0.004151857 |
| Ifi27l2a | -0.930903 | 0.229111 | -0.701671 | -0.651838 | -0.0494682 | 0.881187 | 0.00423602 |
| Arhgap26 | 0.558782 | 0.230727 | 0.789269 | 0.962154 | -0.172804 | -0.731546 | 0.00438875 |
| C030037D09Rik | 0.0695927 | -0.84695 | -0.776968 | -0.156237 | -0.620606 | -0.690432 | 0.004469119 |
| Myl10 | 0.0274745 | -2.44942 | -2.42196 | -2.37494 | -0.0469753 | -0.0744044 | 0.005348681 |
| Parp9 | 1.4541 | -0.952849 | 0.501306 | 1.13557 | -0.634248 | -2.08849 | 0.006203238 |
| Cep290 | 0.79385 | 0.0243688 | 0.817292 | -0.369815 | 1.18654 | 0.393026 | 0.006724514 |
| Agfg2 | 0.179697 | -0.452416 | -0.272394 | 0.459722 | -0.731709 | -0.911758 | 0.006854361 |
| Olfr558 | 0.281434 | -1.04105 | -0.759906 | -0.840568 | 0.0804384 | -0.20093 | 0.007791778 |
| Uprt | -0.553749 | 0.932456 | 0.378829 | 0.826344 | -0.447545 | 0.106146 | 0.007830217 |
| Cx3cl1 | -0.0851364 | -0.830877 | -0.915768 | -1.08706 | 0.171649 | 0.256544 | 0.008320413 |

1. When considering two values, A and B, Log2Fold Change = Log2 (B/A). For example, Log2Fold of the contrast AW-IM = log2(IM/AW).

2. AW-IM refers to the active wild-type vs. inactive myostatin-reduced contrast group

3. AM-AW refers to the active myostatin-reduced vs. active wild-type contrast group

4. AM-IM refers to the active myostatin-reduced vs. inactive myostatin-reduced contrast group

5. IW-AW refers to the inactive wild-type vs. active wild-type contrast group

6. IW-AM refers to the inactive wild-type vs. active myostatin-reduced contrast group

7. IW-IM refers to the jnactive wild-type vs. inactive myostatin-reduced contrast group

**Table B. Enriched (enrichment score > 3.0) clusters of Gene Ontology (GO) biological process (BP), molecular function (MF) Functional Annotation Tool (FAT) categories, and KEGG pathways among differentially expressed genes (FDR-adjusted P-value < 0.01) in the active myostatin-reduced vs jnactive myostatin-reduced contrast group**

| **Category** | **Term** | **Number of Genes** | **P-Value** | **FDR-adjusted P-value** |
| --- | --- | --- | --- | --- |
| Score = 9.68 |  |  |  |  |
| GOTERM BP FAT | GO:0006091~generation of precursor metabolites and energy | 44 | 3.84E-16 | 5.77E-13 |
| KEGG PATHWAY | mmu00190:Oxidative phosphorylation | 31 | 9.99E-14 | 1.20E-10 |
| KEGG PATHWAY | mmu05010:Alzheimer's disease | 36 | 2.63E-13 | 3.17E-10 |
| KEGG PATHWAY | mmu05012:Parkinson's disease | 30 | 1.25E-12 | 1.50E-09 |
| KEGG PATHWAY | mmu05016:Huntington's disease | 34 | 8.71E-12 | 1.05E-08 |
| GOTERM BP FAT | GO:0022900~electron transport chain | 24 | 3.04E-11 | 5.31E-08 |
| GOTERM MF FAT | GO:0022890~inorganic cation transmembrane transporter activity | 17 | 2.80E-05 | 0.042378 |
| GOTERM MF FAT | GO:0015078~hydrogen ion transmembrane transporter activity | 13 | 6.43E-05 | 0.097376 |
| GOTERM MF FAT | GO:0015077~monovalent inorganic cation transmembrane transporter activity | 13 | 1.16E-04 | 0.175144 |

| Score = 5.30 |  |  |  |  |
| --- | --- | --- | --- | --- |
| GOTERM BP FAT | GO:0001525~angiogenesis | 19 | 3.09E-06 | 0.005395 |
| GOTERM BP FAT | GO:0001944~vasculature development | 27 | 3.71E-06 | 0.006479 |
| GOTERM BP FAT | GO:0048514~blood vessel morphogenesis | 23 | 7.17E-06 | 0.012512 |
| GOTERM BP FAT | GO:0001568~blood vessel development | 26 | 7.41E-06 | 0.012931 |

| Score = 3.96 |  |  |  |  |
| --- | --- | --- | --- | --- |
| GOTERM MF FAT | GO:0051540~metal cluster binding | 11 | 1.64E-05 | 0.024771 |
| GOTERM MF FAT | GO:0051536~iron-sulfur cluster binding | 11 | 1.64E-05 | 0.024771 |
| GOTERM MF FAT | GO:0005506~iron ion binding | 28 | 3.36E-04 | 0.50721 |

| Score = 3.94 |  |  |  |  |
| --- | --- | --- | --- | --- |
| GOTERM MF FAT | GO:0048037~cofactor binding | 25 | 6.38E-06 | 0.009665 |
| GOTERM MF FAT | GO:0050662~coenzyme binding | 19 | 4.23E-05 | 0.064092 |

| Score = 3.89 |  |  |  |  |
| --- | --- | --- | --- | --- |
| KEGG PATHWAY | mmu04260:Cardiac muscle contraction | 18 | 6.92E-08 | 8.33E-05 |
| GOTERM MF FAT | GO:0022890~inorganic cation transmembrane transporter activity | 17 | 2.80E-05 | 0.042378 |
| GOTERM MF FAT | GO:0015078~hydrogen ion transmembrane transporter activity | 13 | 6.43E-05 | 0.097376 |
| GOTERM MF FAT | GO:0015077~monovalent inorganic cation transmembrane transporter activity | 13 | 1.16E-04 | 0.175144 |

| Score = 3.35 |  |  |  |  |
| --- | --- | --- | --- | --- |
| GOTERM MF FAT | GO:0001883~purine nucleoside binding | 91 | 3.14E-05 | 0.047562 |
| GOTERM MF FAT | GO:0030554~adenyl nucleotide binding | 90 | 3.79E-05 | 0.057339 |
| GOTERM MF FAT | GO:0001882~nucleoside binding | 91 | 3.96E-05 | 0.059996 |
| GOTERM MF FAT | GO:0032559~adenyl ribonucleotide binding | 81 | 6.06E-04 | 0.914871 |
| GOTERM MF FAT | GO:0017076~purine nucleotide binding | 99 | 6.54E-04 | 0.985956 |

**Table C. Enriched (enrichment score > 2.0) clusters of Gene Ontology (GO) biological process (BP), molecular function (MF) Functional Annotation Tool (FAT) categories, and KEGG pathways among differentially expressed genes (FDR-adjusted P-value < 0.01) in the inactive wild-type vs active wild-type contrast group**

| **Category** | **Term** | **Number of Genes** | **P-Value** | **FDR-adjusted P-value** |
| --- | --- | --- | --- | --- |
| Score = 2.94 |  |  |  |  |
| GOTERM BP FAT | GO:0055114~oxidation reduction | 14 | 1.22E-04 | 0.184857 |
| GOTERM MF FAT | GO:0048037~cofactor binding | 8 | 4.83E-04 | 0.624564 |

| Score = 2.83 |  |  |  |  |
| --- | --- | --- | --- | --- |
| KEGG PATHWAY | mmu04260:Cardiac muscle contraction | 9 | 3.22E-07 | 3.48E-04 |
| KEGG PATHWAY | mmu05410:Hypertrophic cardiomyopathy (HCM) | 6 | 8.26E-04 | 0.888921 |

**Table D. Enriched (enrichment score > 3.0) clusters of Gene Ontology (GO) biological process (BP), molecular function (MF) Functional Annotation Tool (FAT) categories, and KEGG pathways among differentially expressed genes (FDR-adjusted P-value < 0.01) in the inactive wild-type vs inactive myostatin-reduced contrast group**

| **Category** | **Term** | **Number of Genes** | **P-Value** | **FDR-adjusted P-value** |
| --- | --- | --- | --- | --- |
| Score = 4.98 |  |  |  |  |
| GOTERM BP FAT | GO:0048514~blood vessel morphogenesis | 17 | 1.60E-06 | 0.002669 |
| GOTERM BP FAT | GO:0001525~angiogenesis | 13 | 1.08E-05 | 0.017915 |
| GOTERM BP FAT | GO:0001568~blood vessel development | 17 | 2.30E-05 | 0.038295 |
| GOTERM BP FAT | GO:0001944~vasculature development | 17 | 3.09E-05 | 0.051554 |

| Score = 3.04 |  |  |  |  |
| --- | --- | --- | --- | --- |
| KEGG PATHWAY | mmu05410:Hypertrophic cardiomyopathy (HCM) | 10 | 9.83E-05 | 0.114889 |

**Table E. Enriched (enrichment score > 3.0) clusters of Gene Ontology (GO) biological process (BP), molecular function (MF) Functional Annotation Tool (FAT) categories, and KEGG pathways among the genes differentially expressed between active and inactive mice (FDR-adjusted P-value < 0.05)**

| **Category** | **Term** | **Number of Genes** | **P-Value** | **FDR-adjusted P-value** |
| --- | --- | --- | --- | --- |
| Score = 19.79 |  |  |  |  |
| KEGG PATHWAY | mmu00190:Oxidative phosphorylation | 51 | 5.29E-29 | 6.39E-26 |
| KEGG PATHWAY | mmu05012:Parkinson's disease | 50 | 2.05E-27 | 2.47E-24 |
| GOTERM BP FAT | GO:0006091~generation of precursor metabolites and energy | 65 | 2.35E-27 | 4.12E-24 |
| KEGG PATHWAY | mmu05010:Alzheimer's disease | 55 | 7.43E-25 | 8.98E-22 |
| KEGG PATHWAY | mmu05016:Huntington's disease | 53 | 5.65E-23 | 6.82E-20 |
| GOTERM BP FAT | GO:0022900~electron transport chain | 39 | 4.30E-22 | 7.54E-19 |
| GOTERM MF FAT | GO:0015078~hydrogen ion transmembrane transporter activity | 23 | 4.07E-11 | 6.28E-08 |
| GOTERM MF FAT | GO:0015077~monovalent inorganic cation transmembrane transporter activity | 23 | 1.44E-10 | 2.22E-07 |
| GOTERM MF FAT | GO:0022890~inorganic cation transmembrane transporter activity | 26 | 2.46E-09 | 3.79E-06 |

| Score = 8.42 |  |  |  |  |
| --- | --- | --- | --- | --- |
| KEGG PATHWAY | mmu04260:Cardiac muscle contraction | 28 | 1.13E-14 | 1.35E-11 |
| GOTERM MF FAT | GO:0015078~hydrogen ion transmembrane transporter activity | 23 | 4.07E-11 | 6.28E-08 |
| GOTERM MF FAT | GO:0015077~monovalent inorganic cation transmembrane transporter activity | 23 | 1.44E-10 | 2.22E-07 |
| GOTERM MF FAT | GO:0022890~inorganic cation transmembrane transporter activity | 26 | 2.46E-09 | 3.79E-06 |
| GOTERM MF FAT | GO:0016675~oxidoreductase activity, acting on heme group of donors | 10 | 7.32E-07 | 1.13E-03 |
| GOTERM MF FAT | GO:0016676~oxidoreductase activity, acting on heme group of donors, oxygen as acceptor | 10 | 7.32E-07 | 1.13E-03 |
| GOTERM MF FAT | GO:0015002~heme-copper terminal oxidase activity | 10 | 7.32E-07 | 1.13E-03 |
| GOTERM MF FAT | GO:0004129~cytochrome-c oxidase activity | 10 | 7.32E-07 | 1.13E-03 |

| Score = 6.98 |  |  |  |  |
| --- | --- | --- | --- | --- |
| GOTERM MF FAT | GO:0048037~cofactor binding | 36 | 1.59E-09 | 2.45E-06 |
| GOTERM MF FAT | GO:0050662~coenzyme binding | 29 | 4.43E-09 | 6.84E-06 |
| GOTERM MF FAT | GO:0050660~FAD binding | 13 | 1.69E-04 | 0.26 |

| Score = 6.67 |  |  |  |  |
| --- | --- | --- | --- | --- |
| GOTERM BP FAT | GO:0019318~hexose metabolic process | 29 | 2.02E-08 | 3.54E-05 |
| GOTERM BP FAT | GO:0005996~monosaccharide metabolic process | 29 | 2.92E-07 | 5.11E-04 |
| GOTERM BP FAT | GO:0006006~glucose metabolic process | 23 | 1.67E-06 | 2.93E-03 |

| Score = 5.09 |  |  |  |  |
| --- | --- | --- | --- | --- |
| GOTERM MF FAT | GO:0016655~oxidoreductase activity, acting on NADH or NADPH, quinone or similar compound as acceptor | 10 | 3.51E-06 | 5.42E-03 |
| GOTERM MF FAT | GO:0016651~oxidoreductase activity, acting on NADH or NADPH | 13 | 5.18E-06 | 0.01 |
| GOTERM MF FAT | GO:0008137~NADH dehydrogenase (ubiquinone) activity | 9 | 1.24E-05 | 0.02 |
| GOTERM MF FAT | GO:0003954~NADH dehydrogenase activity | 9 | 1.24E-05 | 0.02 |
| GOTERM MF FAT | GO:0050136~NADH dehydrogenase (quinone) activity | 9 | 1.24E-05 | 0.02 |

| Score = 4.17 |  |  |  |  |
| --- | --- | --- | --- | --- |
| GOTERM BP FAT | GO:0006732~coenzyme metabolic process | 25 | 1.61E-07 | 2.83E-04 |
| GOTERM BP FAT | GO:0051186~cofactor metabolic process | 27 | 1.28E-06 | 2.24E-03 |
| GOTERM BP FAT | GO:0045333~cellular respiration | 14 | 5.28E-06 | 9.26E-03 |
| GOTERM BP FAT | GO:0006084~acetyl-CoA metabolic process | 10 | 1.39E-05 | 0.02 |
| GOTERM BP FAT | GO:0009109~coenzyme catabolic process | 9 | 6.35E-05 | 0.11 |
| GOTERM BP FAT | GO:0051187~cofactor catabolic process | 9 | 1.36E-04 | 0.24 |
| KEGG PATHWAY | mmu00020:Citrate cycle (TCA cycle) | 9 | 3.31E-04 | 0.39 |

| Score = 4.14 |  |  |  |  |
| --- | --- | --- | --- | --- |
| GOTERM BP FAT | GO:0009145~purine nucleoside triphosphate biosynthetic process | 19 | 7.81E-07 | 1.37E-03 |
| GOTERM BP FAT | GO:0009142~nucleoside triphosphate biosynthetic process | 19 | 9.19E-07 | 1.61E-03 |
| GOTERM BP FAT | GO:0009144~purine nucleoside triphosphate metabolic process | 20 | 1.12E-06 | 1.96E-03 |
| GOTERM BP FAT | GO:0046034~ATP metabolic process | 18 | 1.92E-06 | 3.37E-03 |
| GOTERM BP FAT | GO:0009205~purine ribonucleoside triphosphate metabolic process | 19 | 2.33E-06 | 4.08E-03 |
| GOTERM BP FAT | GO:0006754~ATP biosynthetic process | 17 | 2.46E-06 | 4.31E-03 |
| GOTERM BP FAT | GO:0009199~ribonucleoside triphosphate metabolic process | 19 | 2.70E-06 | 4.72E-03 |
| GOTERM BP FAT | GO:0006119~oxidative phosphorylation | 14 | 2.83E-06 | 4.97E-03 |
| GOTERM BP FAT | GO:0009201~ribonucleoside triphosphate biosynthetic process | 18 | 3.09E-06 | 5.41E-03 |
| GOTERM BP FAT | GO:0009206~purine ribonucleoside triphosphate biosynthetic process | 18 | 3.09E-06 | 5.41E-03 |
| GOTERM BP FAT | GO:0009141~nucleoside triphosphate metabolic process | 20 | 3.97E-06 | 6.97E-03 |
| GOTERM BP FAT | GO:0009152~purine ribonucleotide biosynthetic process | 19 | 5.46E-06 | 9.58E-03 |
| GOTERM BP FAT | GO:0009150~purine ribonucleotide metabolic process | 20 | 6.67E-06 | 0.01 |
| GOTERM BP FAT | GO:0009260~ribonucleotide biosynthetic process | 19 | 9.29E-06 | 0.02 |
| GOTERM BP FAT | GO:0009259~ribonucleotide metabolic process | 20 | 1.38E-05 | 0.02 |
| GOTERM BP FAT | GO:0006164~purine nucleotide biosynthetic process | 21 | 1.53E-05 | 0.03 |
| GOTERM BP FAT | GO:0006163~purine nucleotide metabolic process | 22 | 4.84E-05 | 0.08 |
| GOTERM MF FAT | GO:0015405~P-P-bond-hydrolysis-driven transmembrane transporter activity | 17 | 6.90E-05 | 0.11 |
| GOTERM MF FAT | GO:0015399~primary active transmembrane transporter activity | 17 | 7.73E-05 | 0.12 |
| GOTERM MF FAT | GO:0042626~ATPase activity, coupled to transmembrane movement of substances | 16 | 7.80E-05 | 0.12 |
| GOTERM MF FAT | GO:0016820~hydrolase activity, acting on acid anhydrides, catalyzing transmembrane movement of substances | 16 | 7.80E-05 | 0.12 |
| GOTERM MF FAT | GO:0043492~ATPase activity, coupled to movement of substances | 16 | 7.80E-05 | 0.12 |
| GOTERM BP FAT | GO:0015992~proton transport | 11 | 2.16E-04 | 0.38 |
| GOTERM BP FAT | GO:0006818~hydrogen transport | 11 | 2.55E-04 | 0.45 |
| GOTERM BP FAT | GO:0015986~ATP synthesis coupled proton transport | 9 | 4.00E-04 | 0.69 |
| GOTERM BP FAT | GO:0015985~energy coupled proton transport, down electrochemical gradient | 9 | 4.00E-04 | 0.69 |
| GOTERM BP FAT | GO:0009165~nucleotide biosynthetic process | 21 | 4.44E-04 | 0.78 |
| GOTERM MF FAT | GO:0042625~ATPase activity, coupled to transmembrane movement of ions | 12 | 5.27E-04 | 0.81 |

| Score = 3.69 |  |  |  |  |
| --- | --- | --- | --- | --- |
| KEGG PATHWAY | mmu00280:Valine, leucine and isoleucine degradation | 15 | 2.13E-07 | 2.58E-04 |
| KEGG PATHWAY | mmu00640:Propanoate metabolism | 11 | 4.84E-06 | 0.01 |
| KEGG PATHWAY | mmu00650:Butanoate metabolism | 11 | 3.86E-05 | 0.05 |
| KEGG PATHWAY | mmu00310:Lysine degradation | 11 | 1.00E-04 | 0.12 |

| Score = 3.29 |  |  |  |  |
| --- | --- | --- | --- | --- |
| GOTERM BP FAT | GO:0003012~muscle system process | 14 | 2.29E-05 | 0.04 |
| GOTERM BP FAT | GO:0006936~muscle contraction | 13 | 3.42E-05 | 0.06 |

| Score = 3.21 |  |  |  |  |
| --- | --- | --- | --- | --- |
| GOTERM MF FAT | GO:0051540~metal cluster binding | 12 | 2.54E-05 | 0.04 |
| GOTERM MF FAT | GO:0051536~iron-sulfur cluster binding | 12 | 2.54E-05 | 0.04 |
| GOTERM MF FAT | GO:0005506~iron ion binding | 33 | 4.22E-04 | 0.65 |

**Table F. Enriched (enrichment score > 3.0) clusters of Gene Ontology (GO) biological process (BP), molecular function (MF) Functional Annotation Tool (FAT) categories, and KEGG pathways among differentially expressed genes (FDR-adjusted P-value < 0.01) in the active wild-type vs inactive myostatin-reduced contrast group**

| **Category** | **Term** | **Number of Genes** | **P-Value** | **FDR-adjusted P-value** |
| --- | --- | --- | --- | --- |
| Score = 15.35 |  |  |  |  |
| GOTERM BP FAT | GO:0006091~generation of precursor metabolites and energy | 70 | 1.05E-28 | 1.85E-25 |
| KEGG PATHWAY | mmu00190:Oxidative phosphorylation | 52 | 2.66E-28 | 3.23E-25 |
| KEGG PATHWAY | mmu05012:Parkinson's disease | 50 | 9.66E-26 | 1.18E-22 |
| GOTERM BP FAT | GO:0022900~electron transport chain | 43 | 1.96E-24 | 3.45E-21 |
| KEGG PATHWAY | mmu05010:Alzheimer's disease | 56 | 6.22E-24 | 7.57E-21 |
| KEGG PATHWAY | mmu05016:Huntington's disease | 56 | 8.38E-24 | 1.02E-20 |
| GOTERM BP FAT | GO:0055114~oxidation reduction | 102 | 1.04E-20 | 1.84E-17 |
| KEGG PATHWAY | mmu04260:Cardiac muscle contraction | 28 | 8.56E-14 | 1.04E-10 |
| GOTERM MF FAT | GO:0015077~monovalent inorganic cation transmembrane transporter activity | 24 | 1.18E-10 | 1.83E-07 |
| GOTERM MF FAT | GO:0015078~hydrogen ion transmembrane transporter activity | 23 | 2.19E-10 | 3.41E-07 |
| GOTERM MF FAT | GO:0022890~inorganic cation transmembrane transporter activity | 26 | 1.43E-08 | 2.23E-05 |
| GOTERM MF FAT | GO:0004129~cytochrome-c oxidase activity | 9 | 1.64E-05 | 0.025624 |
| GOTERM MF FAT | GO:0016676~oxidoreductase activity, acting on heme group of donors, oxygen as acceptor | 9 | 1.64E-05 | 0.025624 |
| GOTERM MF FAT | GO:0016675~oxidoreductase activity, acting on heme group of donors | 9 | 1.64E-05 | 0.025624 |
| GOTERM MF FAT | GO:0015002~heme-copper terminal oxidase activity | 9 | 1.64E-05 | 0.025624 |

| Score = 6.77 |  |  |  |  |
| --- | --- | --- | --- | --- |
| GOTERM MF FAT | GO:0016655~oxidoreductase activity, acting on NADH or NADPH, quinone or similar compound as acceptor | 12 | 6.16E-08 | 9.62E-05 |
| GOTERM MF FAT | GO:0003954~NADH dehydrogenase activity | 11 | 1.93E-07 | 3.02E-04 |
| GOTERM MF FAT | GO:0008137~NADH dehydrogenase (ubiquinone) activity | 11 | 1.93E-07 | 3.02E-04 |
| GOTERM MF FAT | GO:0050136~NADH dehydrogenase (quinone) activity | 11 | 1.93E-07 | 3.02E-04 |
| GOTERM MF FAT | GO:0016651~oxidoreductase activity, acting on NADH or NADPH | 15 | 3.13E-07 | 4.89E-04 |

| Score = 5.05 |  |  |  |  |
| --- | --- | --- | --- | --- |
| GOTERM BP FAT | GO:0045333~cellular respiration | 18 | 1.25E-08 | 2.20E-05 |
| GOTERM BP FAT | GO:0006084~acetyl-CoA metabolic process | 13 | 4.40E-08 | 7.74E-05 |
| KEGG PATHWAY | mmu00020:Citrate cycle (TCA cycle) | 13 | 1.94E-07 | 2.36E-04 |
| GOTERM BP FAT | GO:0006732~coenzyme metabolic process | 26 | 3.11E-07 | 5.47E-04 |
| GOTERM BP FAT | GO:0051186~cofactor metabolic process | 27 | 9.20E-06 | 0.016193 |
| GOTERM BP FAT | GO:0009109~coenzyme catabolic process | 10 | 1.82E-05 | 0.031998 |
| GOTERM BP FAT | GO:0046356~acetyl-CoA catabolic process | 9 | 2.99E-05 | 0.052582 |
| GOTERM BP FAT | GO:0051187~cofactor catabolic process | 10 | 4.38E-05 | 0.077033 |
| GOTERM BP FAT | GO:0006099~tricarboxylic acid cycle | 8 | 1.82E-04 | 0.320662 |
| GOTERM BP FAT | GO:0009060~aerobic respiration | 8 | 5.43E-04 | 0.952398 |

| Score = 4.87 |  |  |  |  |
| --- | --- | --- | --- | --- |
| GOTERM BP FAT | GO:0015980~energy derivation by oxidation of organic compounds | 26 | 8.74E-11 | 1.54E-07 |
| GOTERM BP FAT | GO:0045333~cellular respiration | 18 | 1.25E-08 | 2.20E-05 |
| GOTERM BP FAT | GO:0022904~respiratory electron transport chain | 10 | 1.32E-05 | 0.02321 |
| GOTERM MF FAT | GO:0008121~ubiquinol-cytochrome-c reductase activity | 5 | 1.16E-04 | 0.18018 |
| GOTERM MF FAT | GO:0016681~oxidoreductase activity, acting on diphenols and related substances as donors, cytochrome as acceptor | 5 | 1.16E-04 | 0.18018 |
| GOTERM MF FAT | GO:0016679~oxidoreductase activity, acting on diphenols and related substances as donors | 5 | 1.16E-04 | 0.18018 |
| GOTERM BP FAT | GO:0042773~ATP synthesis coupled electron transport | 7 | 3.07E-04 | 0.539741 |

| Score = 4.41 |  |  |  |  |
| --- | --- | --- | --- | --- |
| GOTERM BP FAT | GO:0001568~blood vessel development | 32 | 1.52E-05 | 0.026737 |
| GOTERM BP FAT | GO:0001944~vasculature development | 32 | 2.48E-05 | 0.043586 |
| GOTERM BP FAT | GO:0048514~blood vessel morphogenesis | 27 | 4.13E-05 | 0.072619 |
| GOTERM BP FAT | GO:0001525~angiogenesis | 20 | 1.44E-04 | 0.252429 |

| Score = 4.37 |  |  |  |  |
| --- | --- | --- | --- | --- |
| GOTERM BP FAT | GO:0003012~muscle system process | 15 | 1.55E-05 | 0.027265 |
| GOTERM BP FAT | GO:0006936~muscle contraction | 14 | 2.06E-05 | 0.036253 |
| GOTERM BP FAT | GO:0006941~striated muscle contraction | 8 | 2.45E-04 | 0.430851 |

| Score = 4.20 |  |  |  |  |
| --- | --- | --- | --- | --- |
| GOTERM BP FAT | GO:0019318~hexose metabolic process | 28 | 6.88E-07 | 0.001212 |
| GOTERM BP FAT | GO:0005996~monosaccharide metabolic process | 29 | 2.52E-06 | 0.004429 |
| GOTERM BP FAT | GO:0006006~glucose metabolic process | 24 | 2.80E-06 | 0.004926 |

| Score = 3.71 |  |  |  |  |
| --- | --- | --- | --- | --- |
| GOTERM BP FAT | GO:0006119~oxidative phosphorylation | 16 | 2.61E-07 | 4.59E-04 |
| GOTERM BP FAT | GO:0009144~purine nucleoside triphosphate metabolic process | 21 | 1.36E-06 | 0.002403 |
| GOTERM BP FAT | GO:0046034~ATP metabolic process | 19 | 1.91E-06 | 0.003354 |
| GOTERM BP FAT | GO:0009205~purine ribonucleoside triphosphate metabolic process | 20 | 2.62E-06 | 0.004605 |
| GOTERM BP FAT | GO:0009199~ribonucleoside triphosphate metabolic process | 20 | 3.05E-06 | 0.005369 |
| GOTERM BP FAT | GO:0009145~purine nucleoside triphosphate biosynthetic process | 19 | 3.67E-06 | 0.00646 |
| GOTERM BP FAT | GO:0009142~nucleoside triphosphate biosynthetic process | 19 | 4.30E-06 | 0.007562 |
| GOTERM BP FAT | GO:0009141~nucleoside triphosphate metabolic process | 21 | 5.08E-06 | 0.008943 |
| GOTERM BP FAT | GO:0009150~purine ribonucleotide metabolic process | 21 | 8.68E-06 | 0.015277 |
| GOTERM BP FAT | GO:0006754~ATP biosynthetic process | 17 | 9.83E-06 | 0.017301 |
| GOTERM BP FAT | GO:0009206~purine ribonucleoside triphosphate biosynthetic process | 18 | 1.30E-05 | 0.022897 |
| GOTERM BP FAT | GO:0009201~ribonucleoside triphosphate biosynthetic process | 18 | 1.30E-05 | 0.022897 |
| GOTERM BP FAT | GO:0009259~ribonucleotide metabolic process | 21 | 1.84E-05 | 0.032458 |
| GOTERM BP FAT | GO:0006164~purine nucleotide biosynthetic process | 22 | 2.25E-05 | 0.039532 |
| GOTERM BP FAT | GO:0009152~purine ribonucleotide biosynthetic process | 19 | 2.39E-05 | 0.042047 |
| GOTERM BP FAT | GO:0006163~purine nucleotide metabolic process | 24 | 2.69E-05 | 0.047409 |
| GOTERM BP FAT | GO:0009260~ribonucleotide biosynthetic process | 19 | 3.97E-05 | 0.069923 |
| GOTERM BP FAT | GO:0044271~nitrogen compound biosynthetic process | 35 | 7.51E-05 | 0.132103 |
| GOTERM BP FAT | GO:0006818~hydrogen transport | 12 | 1.34E-04 | 0.235416 |
| GOTERM MF FAT | GO:0015405~P-P-bond-hydrolysis-driven transmembrane transporter activity | 17 | 1.90E-04 | 0.296366 |
| GOTERM MF FAT | GO:0015399~primary active transmembrane transporter activity | 17 | 2.12E-04 | 0.330822 |
| GOTERM BP FAT | GO:0015992~proton transport | 11 | 5.14E-04 | 0.901149 |

| Score = 3.68 |  |  |  |  |
| --- | --- | --- | --- | --- |
| KEGG PATHWAY | mmu00310:Lysine degradation | 13 | 6.01E-06 | 0.007323 |
| KEGG PATHWAY | mmu00280:Valine, leucine and isoleucine degradation | 13 | 2.21E-05 | 0.026883 |
| KEGG PATHWAY | mmu00380:Tryptophan metabolism | 12 | 2.84E-05 | 0.034604 |
| KEGG PATHWAY | mmu00640:Propanoate metabolism | 10 | 7.27E-05 | 0.088511 |
| KEGG PATHWAY | mmu00650:Butanoate metabolism | 10 | 4.23E-04 | 0.513715 |

| **Category** | **Term** | **Number of Genes** | **P-Value** | **FDR-adjusted P-value** |
| --- | --- | --- | --- | --- |
| Score = 3.62 |  |  |  |  |
| GOTERM MF FAT | GO:0000166~nucleotide binding | 161 | 1.53E-05 | 0.023864 |
| GOTERM MF FAT | GO:0017076~purine nucleotide binding | 138 | 7.69E-05 | 0.120009 |
| GOTERM MF FAT | GO:0001883~purine nucleoside binding | 118 | 8.01E-05 | 0.12499 |
| GOTERM MF FAT | GO:0030554~adenyl nucleotide binding | 117 | 8.53E-05 | 0.132977 |
| GOTERM MF FAT | GO:0001882~nucleoside binding | 118 | 1.02E-04 | 0.158305 |

| Score = 3.25 |  |  |  |  |
| --- | --- | --- | --- | --- |
| GOTERM BP FAT | GO:0042692~muscle cell differentiation | 22 | 1.75E-06 | 0.00308 |
| GOTERM BP FAT | GO:0060537~muscle tissue development | 23 | 5.95E-06 | 0.010466 |
| GOTERM BP FAT | GO:0014706~striated muscle tissue development | 22 | 6.77E-06 | 0.011921 |
| GOTERM BP FAT | GO:0051146~striated muscle cell differentiation | 18 | 7.05E-06 | 0.012407 |
| GOTERM BP FAT | GO:0007517~muscle organ development | 26 | 1.50E-05 | 0.026482 |
| GOTERM BP FAT | GO:0055001~muscle cell development | 14 | 2.49E-05 | 0.04375 |
| GOTERM BP FAT | GO:0055002~striated muscle cell development | 13 | 3.26E-05 | 0.057439 |
| GOTERM BP FAT | GO:0007519~skeletal muscle tissue development | 15 | 3.65E-05 | 0.064225 |
| GOTERM BP FAT | GO:0060538~skeletal muscle organ development | 15 | 5.02E-05 | 0.08835 |
| GOTERM BP FAT | GO:0048741~skeletal muscle fiber development | 9 | 2.87E-04 | 0.504218 |

| Score = 3.13 |  |  |  |  |
| --- | --- | --- | --- | --- |
| KEGG PATHWAY | mmu00640:Propanoate metabolism | 10 | 7.27E-05 | 0.088511 |
| KEGG PATHWAY | mmu00620:Pyruvate metabolism | 11 | 1.98E-04 | 0.24114 |

**Table G. Genes differentially expressed (FDR-adjusted P-value < 0.01) between myostatin-reduced and wild-type mice in the skeletal muscle.**

| **gene** | **log2(Myostatin-reduced/Wild-type)** | **FDR-adjusted P-value** |
| --- | --- | --- |
| Gdap1 | 0.581842 | 0.00246256 |
| Plekhb2 | -0.601226 | 0.00246256 |
| Mstn | -2.75773 | 0.00246256 |
| Ccnyl1 | -0.69498 | 0.00246256 |
| Xrcc5 | 0.467004 | 0.00246256 |
| Plcd4 | 0.566403 | 0.00246256 |
| Utp14b | 0.457476 | 0.00246256 |
| Ramp1 | -0.791397 | 0.00246256 |
| Mybph | 1.33981 | 0.00246256 |
| Glul | 0.685157 | 0.00246256 |
| Esrrg | -0.606473 | 0.00246256 |
| Lpgat1 | -0.434078 | 0.00246256 |
| Snhg6 | 0.632426 | 0.00246256 |
| Ankrd39 | -0.738012 | 0.00246256 |
| Mettl21e | 3.48344 | 0.00246256 |
| Acadl | -0.493922 | 0.00246256 |
| Lancl1 | -1.29096 | 0.00246256 |
| Igfbp5 | 1.07327 | 0.00246256 |
| Tmem37 | -0.709795 | 0.00246256 |
| Igfn1 | -0.827709 | 0.00246256 |
| Prrx1 | -0.415178 | 0.00246256 |
| Mettl11b | -0.751652 | 0.00246256 |
| Atp1b1 | -0.793388 | 0.00246256 |
| Hsd17b7 | 0.937452 | 0.00246256 |
| Grem2 | 1.91334 | 0.00246256 |
| Pld5 | 0.548427 | 0.00246256 |
| Vash2 | 1.30078 | 0.00246256 |
| Mfsd7b | -0.485029 | 0.00246256 |
| Ctgf | -0.790422 | 0.00246256 |
| Vgll2 | -0.622155 | 0.00246256 |
| Reep6 | 1.02852 | 0.00246256 |
| Gadd45b | -0.56809 | 0.00246256 |
| Naca | 0.62951 | 0.00246256 |
| Ddit4 | 1.21417 | 0.00246256 |
| Gamt | 0.69397 | 0.00246256 |
| Lingo3 | 0.600046 | 0.00246256 |
| Socs2 | -0.636216 | 0.00246256 |
| Gdf11 | 0.862347 | 0.00246256 |
| Dusp18 | -0.70463 | 0.00246256 |
| Gpx3 | -0.564418 | 0.00246256 |
| Per1 | 0.992361 | 0.00246256 |
| Unc119 | 0.793727 | 0.00246256 |
| Rhbdl3 | 0.563393 | 0.00246256 |
| Tmem100 | -0.964487 | 0.00246256 |
| Kcnj2 | -0.450971 | 0.00246256 |
| Ttyh2 | 0.727328 | 0.00246256 |
| Shmt1 | 0.707864 | 0.00246256 |
| Atp1b2 | 0.437681 | 0.00246256 |
| Stxbp4 | 0.409813 | 0.00246256 |
| Etv4 | 1.11357 | 0.00246256 |
| Exoc7 | 0.635727 | 0.00246256 |
| Tbc1d16 | 0.535674 | 0.00246256 |
| Fasn | 0.522216 | 0.00246256 |
| Sipa1l1 | 0.749292 | 0.00246256 |
| Fos | -1.20922 | 0.00246256 |
| Exoc3l4 | 1.00016 | 0.00246256 |
| Tnfaip2 | 0.628258 | 0.00246256 |
| Angel1 | 0.607465 | 0.00246256 |
| Rps6ka5 | -0.585073 | 0.00246256 |
| Prima1 | -1.64366 | 0.00246256 |
| Fam65b | 0.856142 | 0.00246256 |
| Gadd45g | 0.700232 | 0.00246256 |
| Wnk2 | 0.938102 | 0.00246256 |
| Map1b | 0.605828 | 0.00246256 |
| Lrtm1 | 0.596474 | 0.00246256 |
| Tnnc1 | -1.3372 | 0.00246256 |
| Mss51 | -1.48168 | 0.00246256 |
| Zcchc24 | 0.408466 | 0.00246256 |
| Prkcd | 0.547909 | 0.00246256 |
| Myh7 | -1.27223 | 0.00246256 |
| Cmbl | 0.649629 | 0.00246256 |
| Lgals1 | 0.490765 | 0.00246256 |
| Triobp | 0.456054 | 0.00246256 |
| Fam19a5 | -0.83434 | 0.00246256 |
| Klf10 | 0.498627 | 0.00246256 |
| St3gal1 | 0.486707 | 0.00246256 |
| Mb | -0.885912 | 0.00246256 |
| Pdgfb | 0.520245 | 0.00246256 |
| Tob2 | 0.56628 | 0.00246256 |
| Bdh1 | -0.606331 | 0.00246256 |
| Dopey2 | 0.522647 | 0.00246256 |
| Gspt1 | 2.38007 | 0.00246256 |
| Dgcr8 | 4.0941 | 0.00246256 |
| Casr | 0.647488 | 0.00246256 |
| Rcan1 | -0.565975 | 0.00246256 |
| Rn45s | 1.13655 | 0.00246256 |
| Supt3 | 0.623895 | 0.00246256 |
| Crip3 | 0.885655 | 0.00246256 |
| Stap2 | 0.668213 | 0.00246256 |
| Egr1 | -0.926777 | 0.00246256 |
| F830016B08Rik | 1.87044 | 0.00246256 |
| Smad7 | -0.609576 | 0.00246256 |
| Nrep | -0.556791 | 0.00246256 |
| Fgf1 | -0.748648 | 0.00246256 |
| Afap1l1 | 0.457003 | 0.00246256 |
| Katnal2 | 1.74421 | 0.00246256 |
| Ankrd2 | -0.587302 | 0.00246256 |
| Golga7b | 0.735187 | 0.00246256 |
| Atrnl1 | -0.592166 | 0.00246256 |
| Ankrd1 | -0.99873 | 0.00246256 |
| Ppp1r3c | 0.690053 | 0.00246256 |
| Sorbs1 | -0.538187 | 0.00246256 |
| Aldh18a1 | 0.866971 | 0.00246256 |
| Rrp12 | 0.432673 | 0.00246256 |
| Sh3pxd2a | 0.435707 | 0.00246256 |
| Otud1 | -0.656853 | 0.00246256 |
| Ralgds | 0.559418 | 0.00246256 |
| Kif5c | 0.892053 | 0.00246256 |
| Itga4 | 0.638377 | 0.00246256 |
| Chac1 | -0.442514 | 0.00246256 |
| Sema6d | -0.45808 | 0.00246256 |
| Smox | 0.556067 | 0.00246256 |
| Cdh4 | 1.8844 | 0.00246256 |
| Pfkfb3 | 0.546962 | 0.00246256 |
| 1700001O22Rik | 0.711038 | 0.00246256 |
| Slc25a25 | 1.04296 | 0.00246256 |
| Itgb6 | -0.415514 | 0.00246256 |
| Smtnl1 | -0.42452 | 0.00246256 |
| Actc1 | 2.29893 | 0.00246256 |
| Tgm2 | 0.479921 | 0.00246256 |
| Pmepa1 | -0.855506 | 0.00246256 |
| Lrrcc1 | -0.87996 | 0.00246256 |
| Skil | -0.48067 | 0.00246256 |
| Kcnab1 | 0.815306 | 0.00246256 |
| Il12a | 1.46618 | 0.00246256 |
| Tpm3 | -1.01634 | 0.00246256 |
| Sort1 | 0.571843 | 0.00246256 |
| Pla2g12a | -0.464197 | 0.00246256 |
| Ddit4l | 0.431241 | 0.00246256 |
| Col24a1 | 0.585311 | 0.00246256 |
| Ddah1 | -1.93497 | 0.00246256 |
| Slc27a3 | 1.13979 | 0.00246256 |
| Plekho1 | 0.516888 | 0.00246256 |
| Myoz2 | -0.988623 | 0.00246256 |
| Dapp1 | -0.721285 | 0.00246256 |
| Cyr61 | -1.01428 | 0.00246256 |
| Fbxl4 | 0.429152 | 0.00246256 |
| Nkain1 | 0.41387 | 0.00246256 |
| Slc30a2 | -0.540219 | 0.00246256 |
| Hspb7 | -0.516134 | 0.00246256 |
| Lrrc38 | 0.458125 | 0.00246256 |
| Ctnnbip1 | 0.623477 | 0.00246256 |
| Pm20d2 | -0.500914 | 0.00246256 |
| Enho | 0.830548 | 0.00246256 |
| Cntfr | 0.812256 | 0.00246256 |
| Ptpn3 | -0.684584 | 0.00246256 |
| Tbc1d1 | -0.628372 | 0.00246256 |
| Stbd1 | 0.542696 | 0.00246256 |
| Arhgap24 | -0.579075 | 0.00246256 |
| Ttc28 | 0.402909 | 0.00246256 |
| Myl2 | -1.56373 | 0.00246256 |
| Aacs | 0.737903 | 0.00246256 |
| Plod3 | 1.13585 | 0.00246256 |
| Evc | -0.609543 | 0.00246256 |
| Ociad2 | 0.690124 | 0.00246256 |
| Mlec | 0.502334 | 0.00246256 |
| Atp2a2 | -1.25922 | 0.00246256 |
| Aimp2 | 0.643739 | 0.00246256 |
| Lmod2 | -0.885277 | 0.00246256 |
| Snd1 | 0.438793 | 0.00246256 |
| St3gal5 | -0.481746 | 0.00246256 |
| Lrrn1 | -0.645526 | 0.00246256 |
| Lmcd1 | -0.633656 | 0.00246256 |
| Ift122 | 0.519497 | 0.00246256 |
| Mgst1 | -0.444133 | 0.00246256 |
| Pdk4 | -0.547792 | 0.00246256 |
| Gadd45a | 0.575148 | 0.00246256 |
| Mthfd2 | 0.788305 | 0.00246256 |
| Cyp26b1 | 0.868973 | 0.00246256 |
| Plxna1 | 0.419592 | 0.00246256 |
| Timp4 | 0.522855 | 0.00246256 |
| Ercc2 | 4.19412 | 0.00246256 |
| Dyrk1b | 0.709851 | 0.00246256 |
| Rhpn2 | 0.861215 | 0.00246256 |
| Mamstr | 0.534735 | 0.00246256 |
| Tmem86a | -0.851344 | 0.00246256 |
| Pak1 | 0.777729 | 0.00246256 |
| Gdpd5 | 0.579543 | 0.00246256 |
| Tnnt1 | -1.42731 | 0.00246256 |
| Vasp | 0.500937 | 0.00246256 |
| Prr12 | 0.506187 | 0.00246256 |
| Csrp3 | -1.22979 | 0.00246256 |
| Nipa1 | 0.97933 | 0.00246256 |
| Gm4980 | 0.421846 | 0.00246256 |
| Dkk3 | -2.30064 | 0.00246256 |
| Mcf2l | 0.662732 | 0.00246256 |
| Dusp26 | -0.918148 | 0.00246256 |
| Rasd2 | 0.480425 | 0.00246256 |
| Plekha2 | 0.535134 | 0.00246256 |
| Lonrf1 | 0.554328 | 0.00246256 |
| Arrdc2 | 1.29248 | 0.00246256 |
| Il15 | 0.611418 | 0.00246256 |
| Ces1d | 0.461645 | 0.00246256 |
| Rrad | -0.769181 | 0.00246256 |
| Hp | -0.664237 | 0.00246256 |
| Slc7a5 | 1.13346 | 0.00246256 |
| Piezo1 | 0.749254 | 0.00246256 |
| Fxyd6 | -1.0769 | 0.00246256 |
| Sln | 1.88691 | 0.00246256 |
| Dnaja4 | -0.484501 | 0.00246256 |
| Cyp1a1 | 1.13089 | 0.00246256 |
| Cilp | -0.862704 | 0.00246256 |
| Dusp7 | 0.496359 | 0.00246256 |
| Myl3 | -1.06792 | 0.00246256 |
| Lars2 | 0.904239 | 0.00246256 |
| Panx1 | -1.09283 | 0.00246256 |
| Pik3cb | -0.571683 | 0.00246256 |
| Slc38a3 | -0.449636 | 0.00246256 |
| Mid1ip1 | 0.547013 | 0.00246256 |
| Stk26 | 0.892347 | 0.00246256 |
| Fhl1 | -0.627755 | 0.00246256 |
| Klhl34 | -1.07143 | 0.00246256 |
| Magix | -0.712636 | 0.00246256 |
| Smarca1 | 0.563635 | 0.00246256 |
| Enox2 | 0.50747 | 0.00246256 |
| Nhs | 0.874941 | 0.00246256 |
| Obsl1 | 0.4324 | 0.00455105 |
| A730008H23Rik | 0.584275 | 0.00455105 |
| Fam83g | 0.712506 | 0.00455105 |
| 2310007L24Rik | 0.694491 | 0.00455105 |
| Grb10 | 0.429562 | 0.00455105 |
| Pabpc1 | 0.406865 | 0.00455105 |
| Map3k7cl | -0.46711 | 0.00455105 |
| C4b | -0.640562 | 0.00455105 |
| Plin3 | 0.422196 | 0.00455105 |
| Pcx | 0.392362 | 0.00455105 |
| Adh1 | 0.547222 | 0.00455105 |
| Slc6a9 | 0.67238 | 0.00455105 |
| Acot7 | -0.454258 | 0.00455105 |
| Arhgef17 | 0.395356 | 0.00455105 |
| Pfkfb4 | 0.391809 | 0.00455105 |
| Tmem45b | -1.2929 | 0.00455105 |
| Armcx4 | -0.437607 | 0.00455105 |
| Slc40a1 | -0.383477 | 0.00644582 |
| Zfp365 | -0.777457 | 0.00644582 |
| Llgl2 | 0.595253 | 0.00644582 |
| Gck | -0.537441 | 0.00644582 |
| Ak4 | 0.509 | 0.00644582 |
| Cited4 | 0.454019 | 0.00644582 |
| Fndc5 | -0.403516 | 0.00644582 |
| Rere | 0.365849 | 0.00644582 |
| Slc25a34 | -0.720531 | 0.00644582 |
| Fam213b | 0.39971 | 0.00644582 |
| Yipf7 | -0.379462 | 0.00644582 |
| Slc7a2 | 0.395351 | 0.00644582 |
| 9530091C08Rik | 0.519554 | 0.00644582 |
| Adhfe1 | 0.414066 | 0.00826513 |
| Btg2 | -0.464529 | 0.00826513 |
| Tpd52l1 | 0.446785 | 0.00826513 |
| Sec14l1 | 0.422324 | 0.00826513 |
| Chrne | 0.515298 | 0.00826513 |
| Fam84b | -0.592906 | 0.00826513 |
| Fam171b | -0.884652 | 0.00826513 |
| Rab13 | 0.549755 | 0.00826513 |
| Ldhb | -0.476312 | 0.00826513 |

**Table H. Enriched (enrichment score > 3.0) clusters of Gene Ontology (GO) biological process (BP), molecular function (MF) Functional Annotation Tool (FAT) categories, and KEGG pathways among differentially expressed genes (FDR-adjusted P-value < 0.005) between skeletal muscle of myostatin-reduced and wild-type mice**

| **Category** | **Term** | **Number of Genes** | **P-Value** | **FDR-adjusted P-value** |
| --- | --- | --- | --- | --- |
| Score = 5.15 |  |  |  |  |
| KEGG PATHWAY | mmu04260:Cardiac muscle contraction | 9 | 3.88E-06 | 0.004373 |
| KEGG PATHWAY | mmu05410:Hypertrophic cardiomyopathy (HCM) | 9 | 6.80E-06 | 0.007672 |
| KEGG PATHWAY | mmu05414:Dilated cardiomyopathy | 9 | 1.34E-05 | 0.015159 |

**Table I. Genes differentially expressed (FDR-adjusted P-value < 0.01) between active and sedentary mice in the skeletal muscle**

| **Gene** | **log2(Active /Inactive)** | **FDR-adjusted P-value** |
| --- | --- | --- |
| Ercc2 | 3.9486 | 0.00154003 |
| Bdh1 | 2.38272 | 0.00154003 |
| Sbk3 | 2.28043 | 0.00154003 |
| BC048679 | 2.27579 | 0.00154003 |
| Lrrc52 | 1.92142 | 0.00154003 |
| Ldhb | 1.88288 | 0.00154003 |
| Tnnc1 | 1.86315 | 0.00154003 |
| Egln3 | 1.82401 | 0.00154003 |
| Myl2 | 1.82118 | 0.00154003 |
| Tnnt1 | 1.7799 | 0.00154003 |
| Myh7 | 1.7768 | 0.00154003 |
| Myom3 | 1.76056 | 0.00154003 |
| Esrrb | 1.74311 | 0.00154003 |
| Slc26a10 | 1.65401 | 0.00154003 |
| Fhl2 | 1.64924 | 0.00154003 |
| Ankrd2 | 1.62966 | 0.00154003 |
| Myh2 | 1.61204 | 0.00154003 |
| Tm6sf1 | 1.5994 | 0.00154003 |
| Iqsec2 | 1.57088 | 0.00154003 |
| Vav2 | 1.52528 | 0.00154003 |
| Tpm3 | 1.50847 | 0.00154003 |
| Myl3 | 1.49783 | 0.00154003 |
| Ak4 | 1.47809 | 0.00154003 |
| Gnmt | 1.4688 | 0.00154003 |
| Mb | 1.46561 | 0.00154003 |
| Casq2 | 1.40514 | 0.00154003 |
| Gstm7 | 1.39107 | 0.00154003 |
| Fam69b | 1.38172 | 0.00154003 |
| Myoz2 | 1.36247 | 0.00154003 |
| Cyp26b1 | 1.324 | 0.00154003 |
| Slc25a34 | 1.29785 | 0.00154003 |
| Casc1 | 1.24002 | 0.00154003 |
| Khdrbs3 | 1.23569 | 0.00154003 |
| Plin5 | 1.19107 | 0.00154003 |
| Hpdl | 1.19048 | 0.00154003 |
| Hmgb3 | 1.17247 | 0.00154003 |
| Fxyd6 | 1.15445 | 0.00154003 |
| Lrrc27 | 1.14944 | 0.00154003 |
| Dgat2 | 1.14667 | 0.00154003 |
| Idh2 | 1.12891 | 0.00154003 |
| Csrp3 | 1.10532 | 0.00154003 |
| Car4 | 1.09632 | 0.00154003 |
| Atp2a2 | 1.09401 | 0.00154003 |
| Fabp3 | 1.08041 | 0.00154003 |
| Arpp21 | 1.07659 | 0.00154003 |
| Nnt | 1.05522 | 0.00154003 |
| Actn2 | 1.0494 | 0.00154003 |
| Rgs2 | 1.04604 | 0.00154003 |
| Chrnd | 1.03828 | 0.00154003 |
| Btnl9 | 1.03204 | 0.00154003 |
| Acer2 | 1.01814 | 0.00154003 |
| Tpsb2 | 1.00692 | 0.00154003 |
| Cish | 1.00023 | 0.00154003 |
| Ckmt2 | 0.992515 | 0.00154003 |
| Ntn4 | 0.964518 | 0.00154003 |
| Npr3 | 0.957444 | 0.00154003 |
| Tspan12 | 0.937989 | 0.00154003 |
| Alpl | 0.932776 | 0.00154003 |
| Adig | 0.931206 | 0.00154003 |
| Cobll1 | 0.905355 | 0.00154003 |
| Atp1b1 | 0.90405 | 0.00154003 |
| Gimap4 | 0.889819 | 0.00154003 |
| Zfp827 | 0.887728 | 0.00154003 |
| Dhrs4 | 0.886307 | 0.00154003 |
| Vopp1 | 0.885488 | 0.00154003 |
| Hspa4l | 0.876428 | 0.00154003 |
| Dach2 | 0.873968 | 0.00154003 |
| Myh13 | 0.87371 | 0.00154003 |
| Timeless | 0.868515 | 0.00154003 |
| Ybx2 | 0.86671 | 0.00154003 |
| Ube2t | 0.866165 | 0.00154003 |
| Col8a1 | 0.862807 | 0.00154003 |
| Acaa2 | 0.852414 | 0.00154003 |
| Lpl | 0.84108 | 0.00154003 |
| 3110057O12Rik | 0.836222 | 0.00154003 |
| Cd36 | 0.833082 | 0.00154003 |
| Fmo2 | 0.83167 | 0.00154003 |
| Dusp18 | 0.820174 | 0.00154003 |
| Tap1 | 0.820024 | 0.00154003 |
| Mdh1 | 0.81314 | 0.00154003 |
| Fndc5 | 0.805109 | 0.00154003 |
| Bmp6 | 0.788763 | 0.00154003 |
| Mt1 | 0.77924 | 0.00154003 |
| Lmcd1 | 0.778753 | 0.00154003 |
| Got1 | 0.772156 | 0.00154003 |
| Trpc3 | 0.770404 | 0.00154003 |
| Pdlim1 | 0.766907 | 0.00154003 |
| Rgs4 | 0.763588 | 0.00154003 |
| Doc2g | 0.757796 | 0.00154003 |
| Smtnl1 | 0.746927 | 0.00154003 |
| Casc4 | 0.746809 | 0.00154003 |
| Esrrg | 0.742136 | 0.00154003 |
| Fhl1 | 0.741041 | 0.00154003 |
| Aldh2 | 0.734166 | 0.00154003 |
| Cited2 | 0.733543 | 0.00154003 |
| Acot11 | 0.732191 | 0.00154003 |
| Rsad2 | 0.728663 | 0.00154003 |
| Nudt7 | 0.72341 | 0.00154003 |
| Emcn | 0.719361 | 0.00154003 |
| Sh2d4a | 0.719027 | 0.00154003 |
| P2ry1 | 0.719015 | 0.00154003 |
| Homer2 | 0.717852 | 0.00154003 |
| Klhl34 | 0.714664 | 0.00154003 |
| Lmod2 | 0.713505 | 0.00154003 |
| Gpt | 0.712249 | 0.00154003 |
| Trp53i11 | 0.709699 | 0.00154003 |
| Car2 | 0.708418 | 0.00154003 |
| Adck3 | 0.703323 | 0.00154003 |
| Fmo1 | 0.699201 | 0.00154003 |
| Slc25a22 | 0.695956 | 0.00154003 |
| Acadl | 0.695387 | 0.00154003 |
| Gfod1 | 0.693489 | 0.00154003 |
| Lars2 | 0.692933 | 0.00154003 |
| Pla2g12a | 0.692537 | 0.00154003 |
| Pkp4 | 0.679442 | 0.00154003 |
| Ddt | 0.677496 | 0.00154003 |
| Xdh | 0.666819 | 0.00154003 |
| Ifi203 | 0.6643 | 0.00154003 |
| Tie1 | 0.66111 | 0.00154003 |
| Rn45s | 0.65945 | 0.00154003 |
| Abcb1b | 0.655513 | 0.00154003 |
| Pgm5 | 0.654806 | 0.00154003 |
| Abcb1a | 0.646431 | 0.00154003 |
| Fabp4 | 0.64633 | 0.00154003 |
| Aldh1l1 | 0.643539 | 0.00154003 |
| Ephx2 | 0.643209 | 0.00154003 |
| Srgn | 0.640638 | 0.00154003 |
| St8sia4 | 0.637636 | 0.00154003 |
| Zfp703 | 0.636276 | 0.00154003 |
| Acadvl | 0.628495 | 0.00154003 |
| Slc41a1 | 0.626471 | 0.00154003 |
| Atp5g1 | 0.624137 | 0.00154003 |
| Lrrn1 | 0.620814 | 0.00154003 |
| Sdhb | 0.619289 | 0.00154003 |
| Fgf1 | 0.616669 | 0.00154003 |
| Nceh1 | 0.614171 | 0.00154003 |
| Arap3 | 0.609285 | 0.00154003 |
| Tstd3 | 0.604964 | 0.00154003 |
| Atp5g3 | 0.604647 | 0.00154003 |
| Ccdc85a | 0.603726 | 0.00154003 |
| Phyh | 0.599642 | 0.00154003 |
| Ednrb | 0.598638 | 0.00154003 |
| Mafb | 0.598584 | 0.00154003 |
| Selenbp1 | 0.591839 | 0.00154003 |
| Etfa | 0.591764 | 0.00154003 |
| Lims2 | 0.591764 | 0.00154003 |
| Eltd1 | 0.590477 | 0.00154003 |
| Pank1 | 0.590065 | 0.00154003 |
| Uqcrq | 0.588689 | 0.00154003 |
| Egfl7 | 0.581595 | 0.00154003 |
| Acss2 | 0.579986 | 0.00154003 |
| Ablim3 | 0.578027 | 0.00154003 |
| Chrna1 | 0.568084 | 0.00154003 |
| Tango2 | 0.565755 | 0.00154003 |
| Anks1 | 0.563899 | 0.00154003 |
| Nos3 | 0.561438 | 0.00154003 |
| Ech1 | 0.554058 | 0.00154003 |
| Kcnj8 | 0.55207 | 0.00154003 |
| Grb14 | 0.551414 | 0.00154003 |
| Cox7a1 | 0.546811 | 0.00154003 |
| Fgd5 | 0.543179 | 0.00154003 |
| Kdr | 0.538269 | 0.00154003 |
| Tek | 0.536642 | 0.00154003 |
| H19 | 0.529955 | 0.00154003 |
| Hspb7 | 0.527609 | 0.00154003 |
| Aqp7 | 0.525323 | 0.00154003 |
| Vgll2 | 0.525321 | 0.00154003 |
| Gpr116 | 0.525203 | 0.00154003 |
| Mybpc1 | 0.52209 | 0.00154003 |
| Hccs | 0.520082 | 0.00154003 |
| Arhgef15 | 0.518629 | 0.00154003 |
| Gstk1 | 0.518453 | 0.00154003 |
| Vegfb | 0.518438 | 0.00154003 |
| Fam195a | 0.517198 | 0.00154003 |
| Evc | 0.515264 | 0.00154003 |
| Oxct1 | 0.515164 | 0.00154003 |
| Prdx5 | 0.512696 | 0.00154003 |
| Cryab | 0.512678 | 0.00154003 |
| Eci1 | 0.512658 | 0.00154003 |
| Etfb | 0.511134 | 0.00154003 |
| Ciapin1 | 0.510084 | 0.00154003 |
| Esam | 0.507629 | 0.00154003 |
| Mtfp1 | 0.506941 | 0.00154003 |
| Etfdh | 0.504616 | 0.00154003 |
| C1qtnf9 | 0.504101 | 0.00154003 |
| Plbd1 | 0.503372 | 0.00154003 |
| Bcam | 0.501392 | 0.00154003 |
| Luzp1 | 0.495779 | 0.00154003 |
| Podxl | 0.494228 | 0.00154003 |
| Hsdl2 | 0.493727 | 0.00154003 |
| Rhou | 0.493294 | 0.00154003 |
| Hadh | 0.492698 | 0.00154003 |
| Perm1 | 0.491619 | 0.00154003 |
| Hadhb | 0.48277 | 0.00154003 |
| Kcng4 | 0.482312 | 0.00154003 |
| Mllt4 | 0.481827 | 0.00154003 |
| Tmem65 | 0.479353 | 0.00154003 |
| Flt1 | 0.479156 | 0.00154003 |
| Ndufb10 | 0.477532 | 0.00154003 |
| Plin3 | 0.475743 | 0.00154003 |
| Ly6c1 | 0.473697 | 0.00154003 |
| Cd300lg | 0.473105 | 0.00154003 |
| Echs1 | 0.472879 | 0.00154003 |
| Tjp1 | 0.472765 | 0.00154003 |
| Ndufb3 | 0.464772 | 0.00154003 |
| Lace1 | 0.462244 | 0.00154003 |
| Nme1 | 0.461506 | 0.00154003 |
| Ppif | 0.459359 | 0.00154003 |
| Slc25a3 | 0.458558 | 0.00154003 |
| Aqp4 | 0.45546 | 0.00154003 |
| Stard10 | 0.454288 | 0.00154003 |
| Hadha | 0.45418 | 0.00154003 |
| Ppara | 0.452888 | 0.00154003 |
| Slc16a1 | 0.44965 | 0.00154003 |
| S100a1 | 0.446048 | 0.00154003 |
| Pecam1 | 0.443207 | 0.00154003 |
| Gm5512 | 0.442853 | 0.00154003 |
| Cox5b | 0.441591 | 0.00154003 |
| Rtn4ip1 | 0.440739 | 0.00154003 |
| Slc25a11 | 0.438894 | 0.00154003 |
| Dhrs11 | 0.438624 | 0.00154003 |
| Acot13 | 0.437207 | 0.00154003 |
| Uqcrfs1 | 0.431014 | 0.00154003 |
| Got2 | 0.429977 | 0.00154003 |
| Cyc1 | 0.428854 | 0.00154003 |
| Cox7b | 0.428438 | 0.00154003 |
| Prdx2 | 0.428254 | 0.00154003 |
| Iscu | 0.423898 | 0.00154003 |
| Cox6c | 0.422185 | 0.00154003 |
| Retsat | 0.41405 | 0.00154003 |
| Ndufaf1 | 0.412772 | 0.00154003 |
| Cdh5 | 0.412207 | 0.00154003 |
| Atp5h | 0.401729 | 0.00154003 |
| Gbe1 | 0.401685 | 0.00154003 |
| Cox4i1 | 0.399712 | 0.00154003 |
| Jam2 | 0.398859 | 0.00154003 |
| Mrpl12 | 0.395978 | 0.00154003 |
| Cox5a | 0.392184 | 0.00154003 |
| Mgll | 0.391295 | 0.00154003 |
| Cav1 | 0.37179 | 0.00154003 |
| Uqcrc2 | 0.367325 | 0.00154003 |
| Ndufv1 | 0.360074 | 0.00154003 |
| Mtss1l | -0.365636 | 0.00154003 |
| Ky | -0.374052 | 0.00154003 |
| St3gal6 | -0.380363 | 0.00154003 |
| Fzd7 | -0.381746 | 0.00154003 |
| Tmem233 | -0.389458 | 0.00154003 |
| Pla2g7 | -0.390142 | 0.00154003 |
| Filip1 | -0.393077 | 0.00154003 |
| Ppp3ca | -0.395445 | 0.00154003 |
| Adamts1 | -0.401933 | 0.00154003 |
| Maf | -0.407044 | 0.00154003 |
| Timp2 | -0.410457 | 0.00154003 |
| Cacng1 | -0.410634 | 0.00154003 |
| Nkain1 | -0.413306 | 0.00154003 |
| Zcchc24 | -0.414946 | 0.00154003 |
| Camk2a | -0.418263 | 0.00154003 |
| Dopey2 | -0.418923 | 0.00154003 |
| Arhgap20 | -0.422603 | 0.00154003 |
| Rsrp1 | -0.423332 | 0.00154003 |
| Ago2 | -0.428181 | 0.00154003 |
| Eda2r | -0.429071 | 0.00154003 |
| Pdlim3 | -0.430501 | 0.00154003 |
| Fbp2 | -0.43205 | 0.00154003 |
| Tob2 | -0.433722 | 0.00154003 |
| Hivep2 | -0.435751 | 0.00154003 |
| Cdk19 | -0.438713 | 0.00154003 |
| Tle4 | -0.439632 | 0.00154003 |
| Vwa7 | -0.455295 | 0.00154003 |
| Stab2 | -0.45872 | 0.00154003 |
| Tmcc1 | -0.460341 | 0.00154003 |
| Setd7 | -0.463317 | 0.00154003 |
| Mast4 | -0.463684 | 0.00154003 |
| Golm1 | -0.465791 | 0.00154003 |
| Tbc1d1 | -0.466045 | 0.00154003 |
| H60b | -0.469386 | 0.00154003 |
| Mettl21c | -0.479534 | 0.00154003 |
| Mllt11 | -0.48246 | 0.00154003 |
| Col22a1 | -0.484867 | 0.00154003 |
| Cpeb1 | -0.487714 | 0.00154003 |
| Nek6 | -0.487822 | 0.00154003 |
| Acvr1b | -0.487881 | 0.00154003 |
| Mylk2 | -0.489361 | 0.00154003 |
| Atp1b2 | -0.489429 | 0.00154003 |
| Map1b | -0.489671 | 0.00154003 |
| Prkag3 | -0.492453 | 0.00154003 |
| Sfxn3 | -0.495974 | 0.00154003 |
| Ddi2 | -0.502468 | 0.00154003 |
| Sorbs2 | -0.504338 | 0.00154003 |
| Crebbp | -0.504805 | 0.00154003 |
| Synm | -0.504848 | 0.00154003 |
| Odf3l2 | -0.506008 | 0.00154003 |
| Hipk2 | -0.508541 | 0.00154003 |
| Prkab2 | -0.516423 | 0.00154003 |
| Ttll7 | -0.519231 | 0.00154003 |
| Myh8 | -0.519775 | 0.00154003 |
| Srebf1 | -0.524354 | 0.00154003 |
| Nrip1 | -0.525698 | 0.00154003 |
| Hoxd8 | -0.533136 | 0.00154003 |
| Osgin1 | -0.535205 | 0.00154003 |
| Ces1d | -0.538897 | 0.00154003 |
| Phka1 | -0.541297 | 0.00154003 |
| Zfp516 | -0.546835 | 0.00154003 |
| Hlf | -0.547732 | 0.00154003 |
| Smyd2 | -0.55304 | 0.00154003 |
| Aebp1 | -0.556014 | 0.00154003 |
| Rasd2 | -0.559099 | 0.00154003 |
| Map3k7cl | -0.559417 | 0.00154003 |
| Klf10 | -0.561837 | 0.00154003 |
| Mybph | -0.563626 | 0.00154003 |
| Trdn | -0.566488 | 0.00154003 |
| Atp8a1 | -0.570274 | 0.00154003 |
| Arrb1 | -0.570845 | 0.00154003 |
| Per1 | -0.571328 | 0.00154003 |
| Hsd17b7 | -0.583758 | 0.00154003 |
| 9530091C08Rik | -0.586562 | 0.00154003 |
| Pfkfb3 | -0.586607 | 0.00154003 |
| Gdf11 | -0.599473 | 0.00154003 |
| Slc2a3 | -0.604728 | 0.00154003 |
| Nt5c1a | -0.626593 | 0.00154003 |
| Gm17296 | -0.629862 | 0.00154003 |
| Gm7120 | -0.650142 | 0.00154003 |
| Cpeb4 | -0.678306 | 0.00154003 |
| Arrdc3 | -0.67888 | 0.00154003 |
| Spon1 | -0.68647 | 0.00154003 |
| Tceal7 | -0.691522 | 0.00154003 |
| F830016B08Rik | -0.708127 | 0.00154003 |
| Kcnab1 | -0.709757 | 0.00154003 |
| Casr | -0.7113 | 0.00154003 |
| Irs2 | -0.711662 | 0.00154003 |
| Lrtm2 | -0.719267 | 0.00154003 |
| Mib1 | -0.721024 | 0.00154003 |
| Mss51 | -0.74542 | 0.00154003 |
| Katnal2 | -0.74856 | 0.00154003 |
| Mylk4 | -0.754098 | 0.00154003 |
| Nhs | -0.78199 | 0.00154003 |
| Tpx2 | -0.859577 | 0.00154003 |
| Ddit4 | -0.865455 | 0.00154003 |
| Tiam1 | -0.867222 | 0.00154003 |
| Cntnap2 | -0.903025 | 0.00154003 |
| Col24a1 | -0.957743 | 0.00154003 |
| Per2 | -0.963405 | 0.00154003 |
| Gck | -1.0384 | 0.00154003 |
| Egr1 | -1.07007 | 0.00154003 |
| Slc25a25 | -1.12004 | 0.00154003 |
| Igfn1 | -1.13402 | 0.00154003 |
| Plod3 | -1.13739 | 0.00154003 |
| Cdh4 | -2.18058 | 0.00154003 |
| Wdyhv1 | 0.956721 | 0.00285251 |
| Paqr4 | 0.789661 | 0.00285251 |
| Frzb | 0.649493 | 0.00285251 |
| Il15 | 0.61618 | 0.00285251 |
| Sp100 | 0.59667 | 0.00285251 |
| Ung | 0.539813 | 0.00285251 |
| Pxmp2 | 0.454652 | 0.00285251 |
| Bgn | 0.450091 | 0.00285251 |
| Qrsl1 | 0.447605 | 0.00285251 |
| 1110058L19Rik | 0.444684 | 0.00285251 |
| Sh3kbp1 | 0.438572 | 0.00285251 |
| 9430020K01Rik | 0.398684 | 0.00285251 |
| Rcan2 | 0.38329 | 0.00285251 |
| Ndufa5 | 0.378496 | 0.00285251 |
| Ndufs6 | 0.367594 | 0.00285251 |
| Wfs1 | 0.363597 | 0.00285251 |
| Sparcl1 | 0.358883 | 0.00285251 |
| Rbfox1 | -0.35037 | 0.00285251 |
| Eef2k | -0.370737 | 0.00285251 |
| Gstm1 | -0.371545 | 0.00285251 |
| Itpr1 | -0.375203 | 0.00285251 |
| Gpd2 | -0.378063 | 0.00285251 |
| Fbxo40 | -0.37865 | 0.00285251 |
| Slc16a10 | -0.390259 | 0.00285251 |
| Epg5 | -0.40187 | 0.00285251 |
| Nqo1 | -0.412587 | 0.00285251 |
| Otub2 | -0.444505 | 0.00285251 |
| Gclc | -0.456087 | 0.00285251 |
| Abat | 0.863978 | 0.0040745 |
| Ecscr | 0.70483 | 0.0040745 |
| Cox6a1 | 0.510958 | 0.0040745 |
| Car8 | 0.508041 | 0.0040745 |
| Cnnm4 | 0.4782 | 0.0040745 |
| Ndufs8 | 0.419862 | 0.0040745 |
| Acat1 | 0.366815 | 0.0040745 |
| Ndufa9 | 0.366716 | 0.0040745 |
| Mrpl45 | 0.366086 | 0.0040745 |
| Osbpl6 | -0.342972 | 0.0040745 |
| Tsc22d3 | -0.358399 | 0.0040745 |
| Arhgef17 | -0.362188 | 0.0040745 |
| Dicer1 | -0.364505 | 0.0040745 |
| Rnf150 | -0.367183 | 0.0040745 |
| Rnf144b | -0.392312 | 0.0040745 |
| Bhlhe40 | -0.39495 | 0.0040745 |
| Bhlhe41 | -0.396438 | 0.0040745 |
| Unc5a | -0.443577 | 0.0040745 |
| 0610011F06Rik | -0.500183 | 0.0040745 |
| Fam179a | 0.724436 | 0.0051237 |
| Cds1 | 0.590548 | 0.0051237 |
| Ralb | 0.575576 | 0.0051237 |
| Arhgap18 | 0.545795 | 0.0051237 |
| Rasgrf2 | 0.539572 | 0.0051237 |
| Plk2 | 0.519085 | 0.0051237 |
| Fam210a | 0.376421 | 0.0051237 |
| Bola3 | 0.375642 | 0.0051237 |
| Ndufa4 | 0.368796 | 0.0051237 |
| Coq10a | 0.360195 | 0.0051237 |
| Sdhd | 0.359523 | 0.0051237 |
| Pnpla2 | 0.358919 | 0.0051237 |
| Atp5j2 | 0.352259 | 0.0051237 |
| Cox7a2 | 0.349972 | 0.0051237 |
| Decr1 | 0.337149 | 0.0051237 |
| Kcnj12 | -0.355699 | 0.0051237 |
| Kif3c | -0.361285 | 0.0051237 |
| Sox6 | -0.38473 | 0.0051237 |
| Cpeb2 | -0.384795 | 0.0051237 |
| Ampd1 | -0.394245 | 0.0051237 |
| Slc43a1 | -0.423996 | 0.0051237 |
| A830082N09Rik | -0.515515 | 0.0051237 |
| Slc25a30 | -0.527803 | 0.0051237 |
| AI506816 | -0.654356 | 0.0051237 |
| Masp1 | 1.13211 | 0.0060736 |
| Stmn2 | 0.865408 | 0.0060736 |
| Gmnn | 0.676701 | 0.0060736 |
| Flt4 | 0.605208 | 0.0060736 |
| Fzd6 | 0.597932 | 0.0060736 |
| Fli1 | 0.545586 | 0.0060736 |
| F11r | 0.543361 | 0.0060736 |
| Car14 | 0.531996 | 0.0060736 |
| Mgp | 0.475908 | 0.0060736 |
| Tsfm | 0.439549 | 0.0060736 |
| Arhgap29 | 0.409994 | 0.0060736 |
| Pcp4l1 | 0.404791 | 0.0060736 |
| Ndufb8 | 0.36101 | 0.0060736 |
| Mical2 | -0.362966 | 0.0060736 |
| Scn4a | -0.365239 | 0.0060736 |
| Adcy9 | -0.390228 | 0.0060736 |
| Bach1 | -0.398533 | 0.0060736 |
| Met | -0.408598 | 0.0060736 |
| Lphn1 | -0.414899 | 0.0060736 |
| Cdkn2c | -0.557379 | 0.0060736 |
| Atp13a5 | -0.558265 | 0.0060736 |
| Fos | -0.562781 | 0.0060736 |
| Dbp | -0.644135 | 0.0060736 |
| Flt3l | 0.853627 | 0.00691535 |
| Idnk | 0.76749 | 0.00691535 |
| G0s2 | 0.678426 | 0.00691535 |
| Unc13b | 0.656822 | 0.00691535 |
| Papss2 | 0.616392 | 0.00691535 |
| Akap17b | 0.588928 | 0.00691535 |
| Efnb2 | 0.501354 | 0.00691535 |
| Dgke | 0.471091 | 0.00691535 |
| Tmem25 | 0.451638 | 0.00691535 |
| Dlst | 0.379448 | 0.00691535 |
| Cpt2 | 0.363751 | 0.00691535 |
| Sptbn1 | 0.355796 | 0.00691535 |
| Acadm | 0.347925 | 0.00691535 |
| 9630033F20Rik | -0.326761 | 0.00691535 |
| Nup153 | -0.346581 | 0.00691535 |
| Amd1 | -0.364579 | 0.00691535 |
| Kank1 | -0.370523 | 0.00691535 |
| Cbr2 | -0.417179 | 0.00691535 |
| Tenm3 | -0.438562 | 0.00691535 |
| D1Ertd622e | -0.457043 | 0.00691535 |
| Cyr61 | -0.480396 | 0.00691535 |
| Bcl2 | -0.484024 | 0.00691535 |
| Fmnl2 | -0.494639 | 0.00691535 |
| Snhg7 | -1.47416 | 0.00691535 |
| Pparg | 0.821226 | 0.00772214 |
| Me2 | 0.665942 | 0.00772214 |
| Pcdh1 | 0.573469 | 0.00772214 |
| Sema6a | 0.556499 | 0.00772214 |
| Mrm1 | 0.477242 | 0.00772214 |
| Dock6 | 0.443232 | 0.00772214 |
| Eid1 | 0.429181 | 0.00772214 |
| Ets1 | 0.420514 | 0.00772214 |
| Foxred1 | 0.414246 | 0.00772214 |
| Stom | 0.35838 | 0.00772214 |
| Atp5d | 0.352745 | 0.00772214 |
| Slc27a1 | 0.35007 | 0.00772214 |
| Rbm20 | -0.352604 | 0.00772214 |
| Dusp1 | -0.36066 | 0.00772214 |
| Utp14b | -0.394872 | 0.00772214 |
| Hdac4 | -0.427016 | 0.00772214 |
| Mmd | -0.433888 | 0.00772214 |
| Gadl1 | -0.487062 | 0.00772214 |
| Meg3 | -0.6548 | 0.00772214 |
| Mmp9 | -0.878654 | 0.00772214 |
| Socs1 | 1.26167 | 0.00863154 |
| Cd274 | 0.595977 | 0.00863154 |
| Iigp1 | 0.513469 | 0.00863154 |
| 41886 | 0.48744 | 0.00863154 |
| Mtx2 | 0.399497 | 0.00863154 |
| Tmem246 | 0.384254 | 0.00863154 |
| Oxnad1 | 0.367047 | 0.00863154 |
| Sdpr | 0.364906 | 0.00863154 |
| Mef2a | -0.348645 | 0.00863154 |
| Cand2 | -0.405777 | 0.00863154 |
| Aldh1l2 | -0.495516 | 0.00863154 |
| Hsbp1l1 | 1.41024 | 0.00950186 |
| Itpripl1 | 0.612148 | 0.00950186 |
| Anp32a | 0.389284 | 0.00950186 |
| Txn2 | 0.329835 | 0.00950186 |
| Rragd | -0.346339 | 0.00950186 |
| Mlxip | -0.348026 | 0.00950186 |
| Pik3ip1 | -0.37842 | 0.00950186 |
| Lrtm1 | -0.379634 | 0.00950186 |
| Ift122 | -0.42331 | 0.00950186 |
| Aurka | -0.477588 | 0.00950186 |
| Hist2h3c1 | -0.509021 | 0.00950186 |

**Table J. Enriched (enrichment score > 2.0) clusters of Gene Ontology (GO) biological process (BP), molecular function (MF) Functional Annotation Tool (FAT) categories, and KEGG pathways among differentially expressed genes (FDR-adjusted P-value < 0.005) between skeletal muscle of active and sedentary mice.**

| **Category** | **Term** | **Number of Genes** | **P-Value** | **FDR-adjusted P-value** |
| --- | --- | --- | --- | --- |
| Score = 8.01 |  |  |  |  |
| KEGG PATHWAY | mmu04260:Cardiac muscle contraction | 20 | 3.78E-14 | 4.42E-11 |
| KEGG PATHWAY | mmu05010:Alzheimer's disease | 26 | 3.13E-12 | 3.67E-09 |
| KEGG PATHWAY | mmu00190:Oxidative phosphorylation | 22 | 8.56E-12 | 1.00E-08 |
| KEGG PATHWAY | mmu05012:Parkinson's disease | 22 | 1.35E-11 | 1.58E-08 |
| GOTERM BP FAT | GO:0006091~generation of precursor metabolites and energy | 26 | 8.07E-11 | 1.34E-07 |
| KEGG PATHWAY | mmu05016:Huntington's disease | 24 | 1.62E-10 | 1.90E-07 |
| GOTERM BP FAT | GO:0022900~electron transport chain | 15 | 4.73E-08 | 7.84E-05 |
| GOTERM MF FAT | GO:0015078~hydrogen ion transmembrane transporter activity | 12 | 7.77E-07 | 0.001127 |
| GOTERM MF FAT | GO:0015077~monovalent inorganic cation transmembrane transporter activity | 12 | 1.42E-06 | 0.00206 |
| GOTERM MF FAT | GO:0015002~heme-copper terminal oxidase activity | 7 | 5.03E-06 | 0.00729 |
| GOTERM MF FAT | GO:0016675~oxidoreductase activity, acting on heme group of donors | 7 | 5.03E-06 | 0.00729 |
| GOTERM MF FAT | GO:0016676~oxidoreductase activity, acting on heme group of donors, oxygen as acceptor | 7 | 5.03E-06 | 0.00729 |
| GOTERM MF FAT | GO:0004129~cytochrome-c oxidase activity | 7 | 5.03E-06 | 0.00729 |
| GOTERM MF FAT | GO:0022890~inorganic cation transmembrane transporter activity | 13 | 1.05E-05 | 0.015201 |

| Score = 4.19 |  |  |  |  |
| --- | --- | --- | --- | --- |
| GOTERM MF FAT | GO:0048037~cofactor binding | 18 | 3.69E-06 | 0.005354 |
| GOTERM MF FAT | GO:0050662~coenzyme binding | 14 | 2.32E-05 | 0.033585 |
| GOTERM MF FAT | GO:0050660~FAD binding | 7 | 0.003159 | 4.486779 |

| Score = 4.11 |  |  |  |  |
| --- | --- | --- | --- | --- |
| GOTERM MF FAT | GO:0051536~iron-sulfur cluster binding | 9 | 6.71E-06 | 0.009739 |
| GOTERM MF FAT | GO:0051540~metal cluster binding | 9 | 6.71E-06 | 0.009739 |
| GOTERM MF FAT | GO:0005506~iron ion binding | 20 | 7.59E-05 | 0.110095 |

| Score = 3.22 |  |  |  |  |
| --- | --- | --- | --- | --- |
| KEGG PATHWAY | mmu00071:Fatty acid metabolism | 9 | 1.56E-05 | 0.018222 |
| KEGG PATHWAY | mmu00280:Valine, leucine and isoleucine degradation | 9 | 1.84E-05 | 0.021581 |
| KEGG PATHWAY | mmu00062:Fatty acid elongation in mitochondria | 5 | 2.75E-05 | 0.032138 |
| KEGG PATHWAY | mmu00650:Butanoate metabolism | 8 | 3.54E-05 | 0.04149 |
| KEGG PATHWAY | mmu00640:Propanoate metabolism | 7 | 9.47E-05 | 0.11081 |
| KEGG PATHWAY | mmu00310:Lysine degradation | 7 | 5.68E-04 | 0.662498 |
